# Distinct host cell proteins incorporated by SIV replicating in CD4^+ ^T Cells from natural disease resistant versus non-natural disease susceptible hosts

**DOI:** 10.1186/1742-4690-7-107

**Published:** 2010-12-16

**Authors:** Susan T Stephenson, Pavel Bostik, Byeongwoon Song, Devi Rajan, Samrath Bhimani, Pavel Rehulka, Ann E Mayne, Aftab A Ansari

**Affiliations:** 1 Department of Pathology & Laboratory Medicine, Emory University School of Medicine, Atlanta, GA 30322, USA; 2 Faculty of Military Health Sciences, University of Defence, Charles University School of Medicine, Hradec Kralove, Czech Republic; 3 Department of Infectious Diseases, Charles University School of Medicine, Hradec Kralove, Czech Republic; 4 Department of Pediatrics, Emory University School of Medicine, Atlanta, GA 30322, USA

## Abstract

**Background:**

Enveloped viruses including the simian immunodeficiency virus (SIV) replicating within host cells acquire host proteins upon egress from the host cells. A number of studies have catalogued such host proteins, and a few have documented the potential positive and negative biological functions of such host proteins. The studies conducted herein utilized proteomic analysis to identify differences in the spectrum of host proteins acquired by a single source of SIV replicating within CD4^+ ^T cells from disease resistant sooty mangabeys and disease susceptible rhesus macaques.

**Results:**

While a total of 202 host derived proteins were present in viral preparations from CD4^+ ^T cells from both species, there were 4 host-derived proteins that consistently and uniquely associated with SIV replicating within CD4^+ ^T cells from rhesus macaques but not sooty mangabeys; and, similarly, 28 host-derived proteins that uniquely associated with SIV replicating within CD4^+ ^T cells from sooty mangabeys, but not rhesus macaques. Of interest was the finding that of the 4 proteins uniquely present in SIV preparations from rhesus macaques was a 26 S protease subunit 7 (MSS1) that was shown to enhance HIV-1 'tat" mediated transactivation. Among the 28 proteins found in SIV preparations from sooty mangabeys included several molecules associated with immune function such as CD2, CD3ε, TLR4, TLR9 and TNFR and a bioactive form of IL-13.

**Conclusions:**

The finding of 4 host proteins that are uniquely associated with SIV replicating within CD4^+ ^T cells from disease susceptible rhesus macaques and 28 host proteins that are uniquely associated with SIV replicating within CD4^+ ^T cells from disease resistant sooty mangabeys provide the foundation for determining the potential role of each of these unique host-derived proteins in contributing to the polarized clinical outcome in these 2 species of nonhuman primates.

## Background

The mechanisms by which non-human primate (NHP) natural hosts of the simian immunodeficiency virus (SIV) remain disease resistant, despite plasma viral loads that in some cases far exceed the levels that lead to a spectrum of disease and death (similar to untreated HIV-1 infection of humans leading to AIDS) in non-natural hosts, remain ill defined [1,2]. Thus while SIV infected sooty mangabeys (SM) and > 40 other African NHP species naturally infected with SIV to a large extent remain disease resistant [3], select isolates from the natural African hosts, when used to experimentally infect non-natural Asian NHP such as rhesus macaques (RM), invariably lead to disease and death [4]. It has been known for some time that enveloped viruses including HIV-1 and SIV interact with and incorporate a variety of host molecules during the various phases of the life cycle of these viruses within the host cell [5]. Thus, as these virions bud and pinch off the plasma membrane of the host cells, they have been shown to carry with them parts of the plasma membrane containing host proteins some of which remain stably associated with the virions. The role these host proteins play while associated with the virions on the infectivity of the virus and/or on the host immune system remains incompletely understood. These findings prompted us to hypothesize that perhaps differences in the nature of the host proteins that interact with and are incorporated by SIV during its life cycle within cell lineages of the disease resistant SM as compared with disease susceptible RM could contribute to the distinct clinical outcome of SIV infection of these two species.

The pioneering studies aimed at the characterization of host proteins incorporated by lentiviruses were performed by the laboratories of Dr. M Tremblay and highlighted the potentially important role such host proteins can play in the pathogenesis of human HIV-1 infection [6]. The initial studies were focused on identifying the mere physical presence of host proteins that had previously been identified as playing a role in immune function [7,8]. These studies were soon followed by reports showing that several of these host proteins, such as the MHC class II proteins, ICAM-1, CD28 and CD40L, indeed could enhance the infectivity of the virions, for some as much as 20 to 100-fold with target cells that expressed the cognate receptors for such molecules [9-13]. In addition, the finding of select host encoded cell adhesion molecules (CAMs) within HIV-1 virions further supported the view that the presence of the previously mentioned immunological receptors along with CAMs could facilitate enhanced cell-cell interaction and thus enhance infectivity of the viruses for target cells that expressed receptors for such CAMs [14,15]. In addition to enhancing viral infectivity, there were also reports of the ability of some of the HIV associated host molecules such as MHC-class II and B7-2 present on both infectious and non-infectious virions to transduce signals that would promote apoptosis of cells bearing receptors for such host proteins [16,17]. The fact that only 0.01 to 0.00001% of the virions in any given virus preparation are in fact infectious suggests that the biological role of such host proteins within inactive virions may play an important role in inducing immune dysfunction characteristic of lentivirus infections [18]. The first detailed study aimed at cataloging the types of host proteins that become associated with HIV-1 was performed by Chertova *et al*. [19] who utilized LC/MS/MS analysis of HIV-1 preparations isolated from infection of enriched populations of human monocyte derived macrophages. A rather substantial list of > 250 host proteins were identified along with 26 of the 37 host proteins previously found to be associated with exosomes.

These findings prompted further studies aimed at defining a) the pathways and the energy barrier being utilized by HIV to bud and egress from cell lineages with the identification of lipid rafts and the virological synapse as being preferentially utilized by HIV-1 [20-22], b) the contribution of microvesicles present in the virus preparations that were being utilized for the analysis of host proteins [23], c) whether the host proteins non-specifically adhere onto the virus or are incorporated within the virus [8,24], and d) the use of more sophisticated and ultrasensitive techniques such as LC-MS/MS to detect the presence of such host proteins [25]. A number of other non-proteomic genome-wide association screening (GWAS) assays utilizing RNA silencing techniques have also been utilized to identify the nature of the host proteins that play critical roles in the life cycle of HIV infection, integration, replication and budding [26-28]. These transient RNA silencing techniques using HeLa/293T cell lines has led to the identification of approximately 272, 278 and 304, to a large extent non-overlapping candidate human genes, that play varying roles in the HIV-1 life cycle. The fact that while these cell lines are relatively easy to perform siRNA transfection studies, but are not the most optimal to study HIV-1 infection that primarily targets T cells prompted Yeung *et al*. [29] to utilize the JURKAT cell line. These authors capitalized on the availability of a shRNA library that targets 54,509 human targets and prepared a large series of JURKAT cloned T cell lines each containing a discrete shRNA and infected these with HIV-1. Such studies led to the identification of 252 host proteins that were critical for HIV-1 replication [29]. In addition, similar series of cataloging studies led to the establishment of a HIV-1 'tat' human nuclear interactome [30], and a HIV-1 Human Protein Interaction Database (HHPID) that is readily available at the NCBI website http://www.ncbi.nlm.nih.gov/RefSeq/HIVInteractions and lists a total of 1435 human genes and 2589 unique HIV-1 protein to host cell protein interactions [31].

The purpose of the studies reported herein was to take advantage of the above findings but focus the studies at the identification of differences in the nature of host proteins incorporated by SIV virions generated by replication within primary CD4^+ ^T cells from disease susceptible RM and disease resistant SM. Data presented herein document the identity of host proteins that are uniquely associated with virions from the 2 species of NHP. The potential role of the proteins identified in contributing to the polarized clinical outcome of SIV infection in the 2 species is discussed.

## Results

In efforts to ensure that the identification of the host proteins incorporated by the virions reflected the physiologically "normal" complement of host proteins, we utilized primary cultures of virus infected cells instead of transformed cell lines. Thus, a series of primary day 3 Con-A blasts from 3 individual rhesus macaques (RM) and 3 individual sooty mangabeys (SM) were utilized for infection with a SIV_del_Table 670 sub-stock, which replicated well in cells from both species. A single pool of the virus preparation from individual monkeys was purified as outlined in the methods section, and aliquots were subjected to studies detailed below.

### Characterization of the virus preparation

As previously documented, the virus preparations when examined by electron microscopy were shown to contain 1-5% virions and large amounts of vesicles and cell debris. A virion purification procedure was therefore utilized using a commercial Fast trap column kit, which led to highly enriched preparation of virus with no detectable vesicles and minimal cell debris. Aliquots of virus preparations from each of the 3 SM and 3 RM were subjected to protein determination, analysis of the levels of p27, relative levels of infectivity (TCID50), and number of viral copies using quantitative PCR prior to the proteomic analysis. Table 1 summarizes the results from these analyses. As seen, while there was considerable amount of variation in the values obtained with each of the assays performed, overall there does appear to be similar distribution of values in the virus preparation from the 2 species of monkeys when comparing p27 levels, TCID50 or number of viral copies. It is important to keep in mind that the same amount of total protein from each of the 3 RM and each of the 3 SM was subjected to analysis and, in addition, the data obtained by proteomic analysis from each sample was analyzed in context with the differences in the values.

**Table 1:** Characterization of the pools of virus prepared from primary cultures of CD4+ T cells from rhesus macaques (RM) and sooty mangabeys (SM)

Monkey Species and ID	Protein (ng/ml)	Levels of p27 (ng/ml)	TCID50 I.U./ml	Viral copy # x 10^7 ^per ml
SM-FYy	784.8	119.41	2.2355	3.13

SM-FMy	474.6	63.03	1.1771	2.76

SM-FJt	384.8	48.7	4.1347	3.83

				

RM-RDd3	1786.9	351.18	4.9960	2.75

RM-RVe7	1059.4	151.30	2.5674	2.79

RM-RLg10	557.6	69.30	1.2364	2.55

### Gel analysis

**Figure 1:**
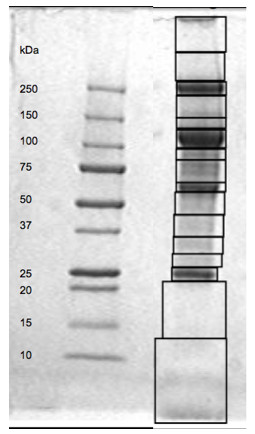
**Representative SDS-PAGE profile of SIV preparations**. A representative SDS-PAGE profile of a virus preparation from CD4^+ ^T cells from a rhesus macaque. The resulting gel was sliced into fragments as indicated by the boxes and subjected to proteomic analysis for the identification of host proteins.

Aliquots (30 μg) of the virus preparation from each of the SM and RM were subjected to 4 to 20% SDS-PAGE analysis in efforts to initially resolve the heterogenous group of proteins. A representative gel profile from the virus pools from a representative RM monkey is depicted in Figure 1. Each of the virus pool from each of the RM and SM gave the same general profile. As seen, there were consistently 4 major bands at approximately 25-30, 60, 100-120 and 250 kDa and a total of 12 additional low and variable intensity bands. The gels were then sliced into 16 similar slices containing these regions (see Figure 1) and each slice subjected to proteomic analysis.

### Proteomic analyses

Data obtained on the spectrum of both viral and host peptides from each gel region of each virus preparation were first entered into a database which could facilitate identifying a list of the different types and the total number of putative proteins that were present in any of the virus preparation from the 2 species of monkeys as described in the methods section. Use of part of the database ftp://ftp.ncbi.nih.gov/refseq/release/vertebrate_mammalian/ for analysis led to the identification of each of the viral proteins (gag-pol polyprotein, env, gag, pol, vif, vpx, vpr, rev, tat and nef). Please note that p27 was not detected in the database search, which we reason is due to the fact that trypsin was used to fragment the proteins, and p27 does not contain a trypsin excision site. However, the presence of p27 in each of the virus preparations was verified by ELISA. Analysis of the databases generated from our study led to the identification of a total of 1979 host proteins (see additional file 1) that were present in viral preparations from any of the 3 RM or any of the 3 SM. These results were then subjected to further analysis to identify those host proteins that were present in viral preparations from both of the 3 SM and 3 RM (common to virus preparations from both the monkey species) (additional file 2) and those that were uniquely present consistently in the virus preparation from each of the 3 RM but none of the SM (Table 2), and those that were uniquely present in virus preparations from each of the 3 SM but none of the RM (Table 3).

**Table 2:** List of host proteins uniquely found in virus from Rhesus macaques (RM) not Sooty Mangabeys (SM).

Host Proteins in only RM-derived Virus	Reference Number	Category
26 S protease regulatory subunit 7 (MSS1 protein)	XP_001118305.1	Ubiquitination, HIV transcription
APG7 autophagy 7-like isoform 4	XP_001088170.1	Ubiquitination
Mitogen-activated protein kinase kinase kinase kinase 1	XP_001082963.1	Intracellular signaling
Tripartite motif-containing 45 isoform 3	XP_001113153.1	Intracellular signaling

**Table 3:** List of host proteins uniquely found in virus from Sooty mangabeys but not Rhesus macaques (RM).

Host Proteins in only SM-derived SIV	Reference Number	Category
2,4-dienoyl CoA reductase 1	XP_001085155.1	metabolism
40 S ribosomal protein S7 (S8)	XP_001095908.1	ribosome
Aldo-keto reductase family 1 member C1	tr|Q0R409|Q0R409_MACFA	metabolism
CD3e molecule, epsilon (CD3-TCR complex)	XP_001097204.1	immune function associated
Chloride intracellular channel 4 isoform 3	XP_001106485.1	membrane/cytoskeleton
Clathrin, heavy polypeptide-like 1	XP_001112729.1	membrane/cytoskeleton
Cluster of differentiation 2 (CD2)	tr|Q6SZ59|Q6SZ59_CERTO	immune function associated
Collagen, type X, alpha 1 precursor isoform 1	XP_001112083.1	extracellular matrix
Complement factor I precursor (C3B/C4B inactivator)	XP_001087512.1	immune function associated
Cryptochrome 2 (photolyase-like)	XP_001113162.1	nuclear protein
Disulfide-isomerase A3-like protein	tr|A6ML76|A6ML76_CALJA	immune function associated
Fc receptor-like and mucin-like 2 isoform 3	XP_001118137.1	immune function associated
Filamin B, beta (actin binding protein 278) isoform 3	XP_001097922.1	membrane/cytoskeleton
Gamma-aminobutyric acid (GABA) A receptor, beta 2 isoform 1 isoform 2	XP_001085738.1	neurotransmission
Glutamate receptor 1	sp|Q38PU8|GRIA1_MACFA	neurotransmission
Guanine nucleotide binding protein-like 3 (nucleolar)-like isoform 2	XP_001090251.1	nuclear protein
HBS1-like isoform 3	XP_001100221.1	immune function associated
Integrin alpha-V	XP_001104012.1	immune function associated
Interleukin-13	tr|Q0ZB84|Q0ZB84_CERTO	immune function associated
MAWD binding protein	XP_001086075.1	immune function associated
Programmed cell death protein 6	XP_001119112.1	immune function associated
Quiescin Q6 isoform a	XP_001111489.1	immune function associated
Ribosomal protein L35a	XP_001082551.1	ribosome
Toll-like receptor 4	tr|B6CJZ3|B6CJZ3_CERTO	immune function associated
Toll-like receptor 9	tr|B6CK02|B6CK02_CERTO	immune function associated
Transcription elongation factor A (SII)-like 4	XP_001085077.1	nuclear protein
Tropomyosin 4 isoform 2	XP_001092183.1	membrane/cytoskeleton
Tumor necrosis factor receptor superfamily, member 17 isoform 1	XP_001106826.1	immune function associated

**Figure 2:**
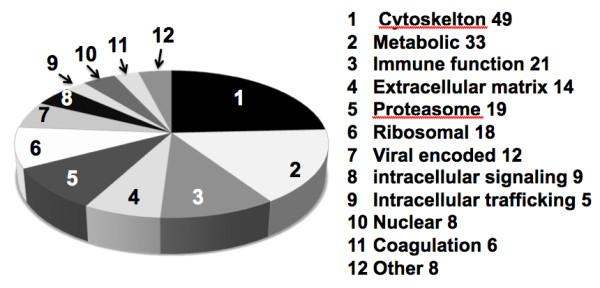
**Categories of host proteins associated with SIV preparations**. The general characterization of the function of the spectrum of host proteins expressed as a percentage of the total (n = 202) that were identified to be present in viral preparations from CD4^+ ^T cells from each of the three rhesus macaques and sooty mangabeys.

This analysis led to the identification of 202 viral and host encoded proteins that were identified in virus preparations from both SM and RM (additional file 2), which is in contrast to the total of 328 proteins identified by Chertova *et al*. in preparations of HIV-1 [19], although these latter studies employed macrophages for their preparation of the HIV-1. As stated above, each of the virus-encoded proteins was identified. In efforts to facilitate an understanding of the potential roles of the host proteins, these data were divided into proteins which represent a) the cytoskeleton (n = 49), b) extracellular matrix (n = 14), c) ribosomal proteins (n = 18), d) proteasome associated (n = 19), e) those involved in intracellular signaling (n = 9), f) those involved in cell metabolism (n = 33), g) those involved in intracellular trafficking (n = 5), h) those associated with coagulation (n = 6), i) those proteins found in the nucleus (n = 8), and j) those with direct or indirect immunological function (n = 21). Thus, as seen in Figure 2 the most abundant group of host proteins present in virus preparations as expected were the cytoskeletal proteins (24.3%), followed by those involved in cell metabolism (16.3%), and of interest a high frequency of proteins involved in immune function (10.4%) which included the cell surface proteins and high levels of the MHC-class I and II molecules, CD44 and CD109 molecules.

As stated above, our major goal in the analyses of the host proteins in the viral preparations was to identify those that are consistently differentially expressed by virus preparations from each of the SM but not RM, and each of the RM but not SM. The database was thus analyzed to identify those proteins that were uniquely present in virus preparations from each of the 3 RM, but not SM and vice versa. Surprisingly, such analyses led to the identification of only 4 host proteins uniquely present in virus preparation from each of the 3 RM, but not in any of the virus preparations from the SM. On the other hand 28 proteins were found to be uniquely present in virus preparations from each of the 3 SM but not any of the RM (Tables 2 and 3). The 4 proteins uniquely identified in viral preparations from each of the 3 RM included a 26 S protease regulatory subunit 7 protein. This 26 S protease is involved in the ATP-dependent degradation of ubiquitinated proteins [32]. The regulatory (or ATPase) complex confers ATP dependency and substrate specificity to the 26 S complex. It has been demonstrated that the 26 S protease regulatory subunit 7 (MSS1 protein) enhances the HIV-1 'tat'-mediated transactivation [33] and associates with basal transcription factors [34,35] suggesting its role in transcriptional regulation. There is also evidence that the 19 S regulatory complex or its subunits functions as mediators of transcriptional systems through their association with promoters, facilitating the clearance of paused elongation complexes, and recruitment of co-activators [36-40]. A recent study also suggested that the proteasome regulates HIV-1 transcription by both proteolytic and nonproteolytic mechanisms [40]. The second protein identified was APG7, which is an E1 like protein involved in autophagy by facilitating the networking of 2 ubiquitin like proteins APg12 and APg8 to associate with E2 enzymes. The third protein identified was the mitogen protein kinase kinase kinase kinase 1 protein involved in cell signaling. The fourth protein identified was TRIM45, which is part of the Tripartitie motif-containing proteins and is thought to serve as a repressor of mitogen activated protein kinase signaling pathway [41]. Thus, these proteins appear to be either involved in ubiquitination or intracellular signaling, with one of them shown to play an important role in HIV-1 transactivation.

As indicated above, the viral preparations from each of the 3 disease resistant SM contained a 7-times higher number of host proteins that were not identified in viral preparations from the disease susceptible RM. This list of proteins was analyzed for their respective function with a bias to define those that have the potential to be involved directly/indirectly with some aspect of immune function. The analysis led to the identification of 14/28 (50%) proteins being directly and/or indirectly involved in immune function, followed by 5 that were classified as being structural and/or membrane associated proteins, 3 that were nuclear proteins, and 2 each involved in cell metabolism, ribosomal proteins, and neurotransmitter proteins. The "immune function related" virus-associated host cell proteins contained important mediators of T cell signaling. Thus, the CD3ε is part of the T cell receptor (TCR) complex and is the main chain that interacts with the TCR [42] and the level of its expression shown to be influenced by disease status in HIV-1 infected individuals [43] resulting in T cell receptor signaling [44]. It is possible that its presence is somehow associated with interference of TCR signaling and thus needs further study. The TNFR superfamily members control diverse aspects of immune function including those mediated by OX40/OX40L interactions. Such interactions regulate CD4^+ ^and CD8^+ ^T cell, NK-T cells, and NK cell function as well as mediating cross talk with antigen presenting cells [45]. The CD2 molecule belongs to the immunoglobulin superfamily of molecules and has been shown to serve as a cell adhesion molecule with LFA-3 (CD58) serving as its ligand [46]. It is expressed by T cells and NK cells and has also been shown to serve a co-stimulatory function [47]. The finding of C3b/C4b inactivator protein is clearly of importance since it is a potent inhibitor of the complement cascade and thus could play a major role in inhibiting the lysis of anti-SIV reactive antibodies. The FcR like and mucin-like protein identified is reminiscent of FcRY, an FcR related gene, which is differentially expressed during B lymphocyte development and activation [48]. The integrin α5 has been shown to be involved in the differentiation of osteoblasts from human bone marrow derived mesenchymal stem cells [49] and hypothesized to similarly induce the differentiation of the monocytoid lineage of cells. Presumably, its presence within the virus particle may be responsible for the accelerated differentiation of these lineages of hematopoietic cells. Of all the immunologically related proteins identified, the presence of CD2, CD3-ε, IL-13, TLR4, TLR9 and the TNFR proteins were thought to be of great interest. Thus, IL-13 is an immuno-regulatory cytokine, which is secreted primarily by Th2 type of helper T cells, and its major role has been shown to involve allergic diseases and immune responses against a number of parasites [50,51]. In addition, IL-13 has been shown to play an important role in the biology of intestinal epithelial cells [52], which are the primary target tissue for both HIV and SIV. Thus, IL-13 has been shown to modulate mucosal epithelial cells by increasing the expression of the pore forming tight junction molecule termed claudin-2 [53]. These findings are of interest in light of the findings of HIV-1 induced dysregulation of claudin-2 in human epithelial cells [54] and its potential role in promoting bacterial translocation [55]. Thus, the IL-13 present in virus replicating in disease resistant SM could be inducing increased claudin-2 synthesis to rapidly repair the damage induced by SIV in the gut mucosa. The presence of TLR-4 in this regard is also of interest since TLR-4 has been shown to be involved in host defense including its role within the gut tissue by responding to LPS and LPS-like ligands and preventing bacterial translocation [56], which has been implicated as playing a major role in inducing chronic immune activation characteristic of pathogenic but not apathogenic HIV/SIV infection [57]. TLR-9, like TLR-7, is a receptor that is activated by nucleic acids or CpG containing immuno-stimulatory motifs. Thus, bacterial and viral infections can induce TLR activation with a number of immunological and hematopoietic consequences. These include the release of a number of cytokines and chemokines but also result in protection from apoptosis of plasmacytoid dendritic cells (pDC's). This issue is important since both bacterial and viral infections not only activate TLR's but also result in the synthesis of glucocorticiod hormones (GC), which are immunosuppressive and lead to apoptosis of pDC's. However, ligation of the TLR7/9 by the CpG like motifs results in the upregulation of the anti-apoptotic genes Bcl-2, Bcl-xl, BIRC3 and CFLAR [58] resulting in survival of the pDC's and preserving the pro-inflammatory pathway leading to protective immune responses. With regards to apoptosis, it is of interest to find the presence of Quiescin 6 in the virions, which is a protein involved in the protection from apoptosis secondary to oxidative stress [59]. The other proteins identified include those that are involved with the gastro-intestinal (GI) tract and include a) disulphide isomerase A3 which has been shown to be a catalytic enzyme that rearranges disulphide bonds in proteins and contribute to immune responses within the GI tract [60]. It has also been shown to be upregulated during alloimmune responses [61], b) MAWBP which is one of the gastric proteins involved in gastric cancer and its ligand MAWD [62] which is known to interact with both the TGF-β receptor and Smd 7 resulting in the inhibition of TGF-β signaling [63]. Finally, it is of interest to note the presence of HBS1, which is an intracellular protein involved in mRNA degradation but is also related to translation factors through direct contact with ribosomes and related to Ski7, which is an accessory molecule to exosomes [64]. Exosomes have been implicated as Trojan horses for the pathogenesis of HIV-1 infection [65,66].

### Bioassays

**Figure 3:**
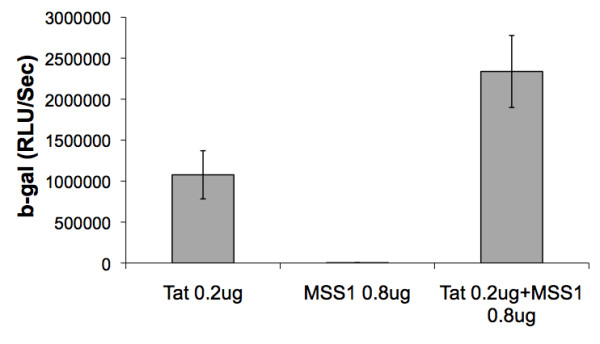
**Enhancement of HIV-1 'tat' mediated transactivation by rhesus MSS-1**. Aliquots of the TZM-bl cell line were transfected with either 0.2 μg of HIV-1 'tat' expression plasmid alone, 0.8 μg of MSS-1 expression plasmid alone, or both and dispensed into individual wells of a 96-well microtiter plate (5000 cells/well) in media for 48 hr. Each assay was performed in triplicate. B-galactosidase activity was then determined using the Tropix Gal-screen assay kit and the results expressed as mean RLU/sec. The data shown are representative of 3 separate experiments. The S.D. of the 3 cultures was all < 10%.

**Figure 4:**
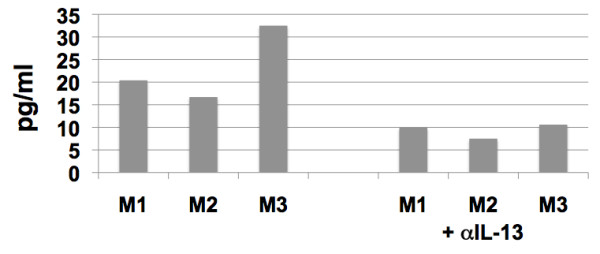
**Presence of functional IL-13 in the SIV preparations from sooty mangabeys**. Assay for IL-13 bioactivity in virus preparations from CD4^+ ^T cells from each of the three sooty mangabeys (SM). The assay was performed as described in the methods section. The values M1, M2, and M3 to the left reflect levels of IL-13 in virus preparations from the 3 SM, and the values to the right are those with the addition of 1/50 dilution of a monoclonal anti-IL-13 antibody. The S.D. of each value shown was < 10%. The lower limits of this assay were determined to be 5 pg/ml using recombinant human IL-13.

The identification of the presence of proteins such as MSS-1 in SIV from each of the 3 RM but none of the 3 SM and IL-13, TLR 4, TLR9 and TNFR in SIV from each of the SM but none of the 3 RM prompted us to confirm their presence using either bioassays and/or Western Blot assays. Unfortunately, none of these molecules could be detected in the purified virus preparations by standard Western Blot assays, which was not due to technical issues since positive controls (recombinant proteins) utilized in parallel showed readily detectable bands. We reason that such failure was likely a result of either denaturation of these proteins secondary to the techniques utilized to prepare highly purified preparations of the virus or due to the limits of the detection by the Western Blot assay. However, we were able to demonstrate that indeed MSS-1 derived from RM does enhance HIV-1 'tat' mediated transactivation (Figure 3). In addition, preliminary studies appear to indicate that CD4^+ ^T cells from RM appear to contain 10-20 times more MSS-1 as compared with similar number of CD4^+ ^T cells from SM which we submit could account for its differential incorporation in SIV from RM. Another assay that appeared to provide meaningful results was the assay for IL-13, as described in the methods section. The results as shown in Figure 4 show that I μg of virus preparation from each of the 3 virus preparations from the CD4^+ ^T cells of SM contained variable amounts of bioactive IL-13. The fact that a monoclonal anti-IL-13 antibody (1/50 dilution) neutralized the bioactivity indicated an element of specificity for the detection of the IL-13 in the virus preparation. No detectable IL-13 bioactivity was noted in the virus preparations from each of the 3 RM (< 5 pg/ml) even when 5 μg of the virus preparation from these monkeys was used in the same assay run in parallel.

Taken together, the above data indicate that SIV replicating in primary CD4^+ ^T cells from SM appears to incorporate a wide array of host proteins as compared with the same virus replicating in primary CD4^+ ^T cells from RM. Of importance was the finding that while a large number of these host proteins uniquely associated with SIV generated from CD4^+ ^T cells from SM appear to be directly and/or indirectly related to immune function, those few that are uniquely associated with SIV from RM are involved with promoting 'tat' mediated transactivation of HIV-1, autophagy and intracellular signaling. How such proteins contribute to the polarized clinical outcome of infection in these 2 species remains to be defined and is a subject of future studies.

## Discussion

The incorporation of host proteins by enveloped virions while they are being packaged within a cell and as they exit from the cell are reasoned to be acquired by the virions as a result of intra-cellular interactions between the various viral proteins and the host proteins [67]. These interactions facilitate the life cycle of the virus and in some cases play an integral role in the escape of the virus from normal host defenses. There are several other proteins incorporated by the virions in fact that have been shown to play an active role in viral entry, integration, transcription, assembly and budding [5,68]. It is important to distinguish host proteins that "interact" with viral proteins and are required and/or facilitate specific stages of the viral life cycle from those host proteins that are "incorporated" by the virions during the various stage of its life cycle. An example of the former is the recent characterization of 19 host proteins that appear to specifically interact with the pre-integration complex (PIC) of HIV-1 [69]. A large number of host proteins that "interact" with select HIV-1 proteins such as HIV-1 'tat' [30] and others that are required for viral entry, reverse transcription, integration, transcription, packaging and exit from the cell are exemplified by the findings of a series of studies that utilized siRNA and shRNA technologies. The study utilizing siRNA has led to the identification of 273 host proteins that have been termed Host Dependency Factors (HDF) that are required for HIV to infect, replicate and package within a permissible host cell [26]. The study utilizing shRNA capitalized on the availability of a library of 54,509 shRNA led as described above to the identification of 252 human candidate genes that play a role in HIV-1 infection [29]. Of interest is the finding of a relative lack of similarity in the spectrum of host proteins that have been catalogued by such approaches. It is reasoned that while there are clear benefits with using such cell lines, the transformed nature and non-physiological relevance of these cell lines as targets of HIV-1 infection and replication may be the basis for the results obtained. When we analyzed the data reported herein (additional file 1) with the databases compiled by the other studies, we found 79/273 described by Chertova *et al*. [19], 36/273 described by Brass *et al*. [26], 54/183 described by Gautier *et al*. [30] and 40/252 described by Yeung *et al*. [29] as summarized under Table 4. A list of the host proteins found common between the studies described herein and those by Chertova *et al*. [19], Brass *et al*. [26], Gautier *et al*. [30] and Yeung *et al*. [29] is provided under the additional files 3, 4, 5 and 6.

**Table 4:** Analysis of host proteins identified in SIV replicating in primary CD4+ T cells from rhesus macaques and sooty mangabeys that have been previously found also to interact with and/or be present in HIV-1 preparations

Type of Analysis	Host cell utilized	Number of host proteins present in SIV/referenced study	Reference
Host proteins in HIV-1 preparation	Human primary macrophages	79/253	[19]

			

GWAS type of study using shRNA/HIV-1	HeLa	37/273	[26]

			

Host proteins interacting with HIV-1 tat	Jurkat	54/183	[30]

			

Host proteins contributing to productive HIV-1 replication	Jurkat	40/252	[29]

It is beginning to become clear that a number of these host cellular proteins that become "associated" or "incorporated" by the virus as they exit from the cell can not only influence the biology of the virus (by increasing or decreasing its level of infectivity) but may also function to enhance or suppress immune responses *in vivo *[70]. While a number of elegant studies have been published on the characterization of host proteins that are associated with HIV-1, the primary purpose of the studies reported herein were focused on determining whether an aliquot of the same virus stock that replicates well and quite similarly within CD4^+ ^T cells from both species included in the present study would differentially acquire host proteins during replication, assembly and egress from cells from disease resistant sooty mangabeys (SM) as compared with the same cell lineage from the disease susceptible rhesus macaques (RM). It should be emphasized that we utilized cultures of primary CD4^+ ^T cells thus eliminating the potential artifacts introduced with the use of transformed cell lines, although the cell lines do provide a larger source of virus and are relatively easy to prepare. As stated above, our studies were designed as such to primarily identify those proteins that are exclusively associated with either pathogenic or apathogenic course of SIV infection, which could lead to an elucidation of some of the pathogenic mechanism underlying SIV disease.

There are several issues that need to be addressed with regards to the studies reported herein, including a) the validation that indeed the proteins identified are truly associated with the virus preparation and not a contaminant, b) whether any of the proteins identified demonstrate function, c) reasons for the marked increases in the number of proteins identified in the SIV prepared from SM versus RM, d) the biological relevance of the proteins identified, and e) the relative sensitivity and specificity of the findings of our studies. These are each addressed below.

**Figure 5:**
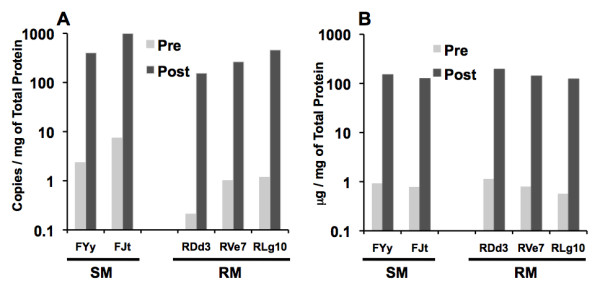
**Analysis of viral copies and levels of p27 in the virus preparations prior to (Pre) and following enrichment (Post)**. An aliquot of the pooled virus from 2 SM (FYy and FJt) and 3 RM (RDd3, RVe7 and RLg10) was analyzed for the number of viral copies and levels of p27 prior to and post enrichment. Values shown reflect (A) number of viral copies per mg of total protein and (B) μg of p27 per mg of total protein.

One of the most important issues with studies related to the identification of host proteins in viral preparations is to distinguish those host proteins that are mere contaminants that co-purify with the virus as compared with proteins that are truly part of the virions. To address this issue, our laboratory conducted a series of studies designed to isolate as pure a virus preparation as technically possible. This required removal of cell debris and other contaminants as outlined in the methods section. Electron micrographic analysis of the virus preparations prior to and post virus purification (see additional file 7) shows the degree of purity achieved using the strategy outlined. Studies on the level of p27 and number of viral copies per mg protein showed a > 100-fold increase per mg total protein in the levels of p27 and SIV viral copies recovered following purification (Figure 5A and 5B). The fact that this purification protocol resulted in the almost complete removal of cell debris provides some degree of assurance that indeed the host proteins identified are highly likely to be associated with the virions and not mere contaminants. Secondly, it is to be noted that the fact that the host proteins identified uniquely associate with virus preparations from each of the 3 SM but NONE of the virus preparations from all 3 RM and vice versa strongly suggests an element of specificity. It is clear that additional studies of the role these host proteins play in viral host interactions may provide added confidence that indeed their presence is not an artifact. As far as function is concerned, we were successful in demonstrating that the MSS-1 protein from RM did show marked enhancement of HIV-1 'tat' mediated transactivation (Figure 3) which is likely due to the finding of the presence of significantly higher levels of MSS-1 within CD4^+ ^T cells from RM as compared with SM. In addition, we were also able to document the presence of IL-13 bioactivity (Figure 4) in virus preparation from SM but not RM, which supports the view that at least some of the host proteins identified could be contributing to differences in the clinical outcome of SIV infection in these 2 species. With regards to the reasons for the increased numbers of host proteins that were identified in the virus preparation from SM as compared with RM, it is important to note that our laboratory has previously shown that CD4^+ ^T cells from SM are resistant to undergo anergy [71] and requires a minimal or no second signal for T cell responses. It is our hypothesis based on these findings that perhaps, CD4^+ ^T cells from SM remain in culture longer than CD4^+ ^T cells from RM, which allows a longer time period for virus to replicate within this cell lineage. On the other hand it should also be noted that SM have a markedly lower frequency of CCR5 expressing CD4^+ ^T cell subset [72] and a skew in the predominance of the TH2 subset [1]. Thus, such differences may promote the replication of SIV within different subsets of CD4^+ ^T cells from SM and RM resulting in the differences in the complement of host proteins that become associated with the virus. This line of reasoning implies that CD4^+ ^T cells from SM include the CD4^+ ^T cell subset present in RM in which the virus replicates and thus cancels out the long list of proteins that were found to be associated with virus preparations from RM. It is important to point out that there was no shortage in the number of host proteins that were found to be associated with virus preparations from RM but the studies herein were focused on identifying only those that were uniquely associated with the species. In this regard, it is also important to keep in mind that while the list of proteins identified is large, it is clear that it is impossible for each virion to include all of these host proteins. In addition, the virus preparations contain a heterogenous selection of viruses, which may contain variable amounts of each of these proteins. As such, we are identifying what is more or less an average group of host proteins that get associated with the virus from each of the 2 species.

One of the most important issues concerning these studies is the biological significance of the findings. Clearly as has been previously described the presence of virus-associated host proteins are of significant consequence as they can serve to a) promote cell to cell transmission of the virus [14], b) induce NF-κB and NFAT activation [16], c) the virions can act as antigen presenting cells since they contain both intact MHC class II and CD86 [73] and d) contain a long list of molecules involved in the induction and regulation of immune responses including HLA-Dr, ICAM-1, CD40, CD40L and CD86 [13]. In addition, select molecules present within these viruses also have been implicated in inducing immunosuppression and contributing to innocent bystander apoptosis highlighting the potential important role such host proteins can play in the pathogenesis of HIV/SIV infection. Germane to the present studies, it is important to identify a specific biological role for proteins uniquely found in virus preparations from the RM and the SM in efforts to determine their role in disease susceptibility/resistance. In this regard, it is important to highlight the role of the host protein MSS1 that was found to be uniquely associated with virus preparations from RM, but not SM (Table 2). Thus, the subunit unit 7 of the 26 S proteosome was identified as MSS1 [74], which was shown to be one of the 'tat' binding proteins (TBP-1) [34] that regulates HIV-1 transcription by both by a proteolytic and a non-proteolytic mechanism [40]. Interestingly, MSS1 has also been shown to play a critical role in regulating CIITA activity and MHC class II transcription [36]. While preliminary data indicate that the differential incorporation of MSS-1 by virus replicating within CD4^+ ^T cells from RM but not SM could be due to quantitative differences in the constitutive level of MSS-1 present in RM as compared with SM, the reasons for such differential intracellular levels is currently under study. The normal physiological role of the other host proteins identified has been outlined above in the results section but their role in promoting disease resistance requires study. We are cognizant that the disease resistance may not in fact be related to these differentially identified host proteins, but could be due to differences in the response of the host to the proteins that are present in virus preparations from both species and/or due to issues distinct from the presence of host proteins. However, it is a reasonable hypothesis to pursue.

Finally, it is important to address the role of the sensitivity and specificity of the list of proteins that were identified. It should be noted that while there were just 3 viral preparations from each of the 2 species, we chose to utilize highly stringent criteria for inclusion of these proteins with a high scoring threshold and confidence levels of > 97%. Thus, the inclusion of proteins being uniquely present in one species and not the other required for a signature sequence be present in preparations from all 3 monkeys and that there were a minimum of 3 hits for each protein. We submit that these are extremely labor intensive studies and require considerable resources for performing such analyses.

## Conclusions

Highly sensitive differential proteomic analysis of SIV preparations from primary CD4^+ ^T cells from 3 sooty mangabeys (the natural disease resistant hosts of SIV) and 3 rhesus macaques (the non-natural disease susceptible hosts) were carried out. These studies led to the identification of 202 host proteins that were found in virus preparations from both rhesus macaques and sooty mangabeys, a total of 28 host proteins that uniquely associated with SIV from each of the 3 sooty mangabeys but none of the rhesus macaques, and 4 host proteins that were uniquely associated with SIV from each of the 3 rhesus macaques but none of the sooty mangabeys. The finding of host proteins such as MSS1 that enhances HIV-1 'tat" mediated transactivation unique to SIV from rhesus macaques and a series of molecules associated with immune function such as IL-13 found in SIV from sooty mangabeys provides a reasonable foundation to initiate studies on the potential link of these unique proteins to the polarized clinical outcome of SIV infection in these 2 nonhuman primate species.

## Methods

### Animals

Peripheral blood samples were obtained from 3 normal healthy adult rhesus macaques (Macaca mulatta) denoted as RM and 3 SIV naïve sooty mangabeys (Cercocebus atys) denoted as SM. All monkeys were maintained at the Yerkes National Primate Research Center of Emory University according to the guidelines of the Committee on the Care and Use of Laboratory Animals of the Institute of Laboratory Animal Resources, National Research Council and the Health and Human Services guidelines "Guide for the Care and Use of Laboratory Animals."

### Isolation and in vitro infection of PBMCs

PBMCs were isolated from freshly obtained heparinized peripheral blood by standard gradient centrifugation using Lymphoprep lymphocyte separation medium (AXIS-SHIELD PoC AS, Oslo, Norway). The cells were first depleted of CD8^+ ^T cells using Dynabeads-CD8 (Invitrogen, Carlsbad, CA) and maintained in vitro in RPMI 1640 media supplemented with 10% FBS, 2 mM L-glutamine, 50 μg/ml gentamicin and 50 U/ml IL-2 at a density of 2 × 10^6 ^cells/ml.

These in vitro cultured cells were activated by co-culture with 2 μg/ml Con-A for 72 hours, then washed and transferred to 50 ml conical tubes and spin inoculated with SIV_del_Table 670 at 1200 × g for 2 hours at room temperature. The pelleted cells were washed 2× in PBS to remove residual virus and then cultured at of 2 × 10^6 ^cells/ml in RPMI 1640 media supplemented as above. Culture media was removed every other day after 3 days of culture, and supernatants cleared by centrifugation at 3220 × g for 20 minutes. A small aliquot of the cultured cells was monitored daily by trypan blue staining to determine the extent of cell death and cultures were terminated when the level of dead cells exceeded 30%. Levels of p27 were quantified for each supernatant sample using SIV p27 Antigen ELISA (ZeptoMetrix Corporation, Buffalo, New York). Control cultures of CD8^+ ^depleted PBMCs from each monkey were maintained in parallel with the SIV infected samples under identical conditions.

### Purification of Virus from Supernatants of Infected Cells

Clarified samples from each of the 3 RM and 3 SM monkeys with the highest p27 levels were ultra-centrifuged and the pelleted virus preparations further purified using the Fast Trap Lentivirus Purification and Concentration Kit (Millipore, Billerica, MA) per manufacturer's instructions. Briefly, clarified supernatants were further filter purified and the sample subsequently bound to an anion exchange membrane. The membrane was washed and the virus eluted using a high salt buffer. The virus was then concentrated and stored in PBS pH 7.4 at -80°C until further use.

### Quantitation of p27, the levels of infectivity (TCID50), number of viral copies and protein concentration

Aliquots of each virus preparation were subjected to a) determination of the levels of p27 using the commercial p27 ELISA kit which included a standard that was utilized to calculate the levels of p27 in the specimens, b) analysis of the relative levels of infectious particles using the CEM cell line. The TCID50 of each of the virus preparation was calculated by standard end point titration whereby 2-fold dilutions of the viral preparation were dispensed in triplicates into individual wells containing 10^5 ^of the cell line and allowed to incubate for 4 hrs, washed and then cultured in media and the supernatant monitored for levels of p27, c) number of viral copies per ml of the virus preparation using quantitative PCR and a set of gag primer pairs as described elsewhere [75,76], and d) quantitation of levels of protein using the BioRad protein determination kit (BioRad, Hercules, CA) utilizing the manufacturers protocol. These procedures led to > 100-fold increase in the levels of p27 and number of viral copies adjusted to 1 mg of protein in preparations of the virus prior to and post purification as illustrated in Figure 5A and 5B. Electron microscopic analysis of the virus preparation similarly demonstrated a marked enrichment of viral particles and the depletion of cellular debris (additional file 7).

### Protein Identification by Mass Spectrometric Analysis

Protein samples were resolved on a SDS gel and stained by Coomassie blue. Selected gel regions were excised (Figure 1) and subjected to in-gel digestion. The resulting peptides were analyzed by reverse-phase liquid chromatography coupled with tandem mass spectrometry [77] using an LTQ-Orbitrap mass spectrometer (Thermo Finnigan, San Jose, CA). The databases searched were derived from ftp://ftp.ncbi.nih.gov/refseq/release/vertebrate_mammalian/of the NCBI Refseq project and utilized available primate, rhesus macaque, sooty mangabey and SIV viral protein sequences. A strategy of reverse database analysis was used to evaluate false discovery rate; the matched peptides were filtered according to matching scores to remove all false matches from the reverse database [78]. This is a highly stringent filter system employed to make sure that the proteins being identified were in fact present in the viral preparation. Thus only proteins that were matched by at least a set of peptides unique to the specific protein in a single virus preparation were selected for addition to the resulting virus-host protein database. Results were analyzed using Microsoft Access.

### Immunoblotting

Samples (30 μg of protein per sample) were separated on SDS-PAGE using a 4-20% gradient ReadyGel (Bio-Rad), transferred to nitrocellulose membrane (BioRad) and the membrane blocked with 5% non-fat milk in T-TBS prior to incubation with the indicated antibody overnight. After being washed 3 times with T-TBS, the membrane was incubated with the appropriate secondary antibody conjugated to HRP for 1 hour at room temperature. All bands were visualized using the ECL detection system (Amersham Biosciences). Equal loading of the samples was determined using anti-β actin antibodies with each analysis (Sigma). The series of IL-13 monoclonal antibodies were a generous gift from Dr. N. Ahlborg of the MabTech corporation (Nacka Strand, Sweden) and the clone showing the highest reactivity (clone IL-13-3) against recombinant rhesus IL-13 was used at a concentration of 1:1000 in T-TBS containing 5% BSA. Bands for IL-13 were visualized using anti-rat IgG conjugated to HRP (Southern Biotech, Birmingham, AL). Anti-TLR4 and TLR9 antibodies were purchased from Abcam (Cambridge, MA) and bands visualized using anti-goat and anti-mouse IgG conjugated to HRP (Southern Biotech, Birmingham, AL), respectively.

### Bioassays

The IL-4 and IL-13 dependent B9 cell line was utilized to determine the presence of biologically active IL-13 in aliquots of the same viral preparations as were used for proteomic analyses. The B9 cell line was starved off cytokines overnight, washed and 100,000 cells in a volume of 50 μl of media were then dispensed into individual wells of a 96-well microtiter plate. Varying concentrations of recombinant human IL-13 was added to triplicate wells in efforts to derive a standard curve to be utilized for the quantitation of the level of potential IL-13 that was present in the viral preparation. The viral preparation from RM and SM were adjusted to 5 μg/ml protein concentration and diluted 2-fold in media and each dilution added in a volume of 50 μl of media to triplicate wells in the presence/absence of 50 μl of a 1/10 dilution of neutralizing anti-IL13 monoclonal antibody (known to neutralize 100 ng/ml of recombinant IL-13). The plates were incubated for 48 hrs in a 7% CO_2 _humidified atmosphere and 16 hrs prior to harvest each well was labeled with 1 μCi of methyl-3H-thymidine and the mean uptake of ^3^H-thymidine determined. The lower limit of this assay was determined to be 5 pg/ml.

The ability of the MSS-1 protein to enhance HIV-1 'tat' mediated transactivation was utilized by our laboratory in efforts to initiate studies on the differential role of this protein in cells from RM and SM. Briefly, the MSS-1 was first cloned into an expression plasmid and used in conjunction with a HIV-1 'tat' expression plasmid to transfect the standard TZM-bl cells. The amount of HIV-1 'tat' plasmid to be used in the assay was first titrated and the level that showed low levels of β-galactosidase activity (0.2 ug of plasmid) chosen. The TZM-bl cells were seeded in 96-well plates (5000 cell per well) and transfected with 0.2 ug of the HIV-1 'tat' expression plasmid, 0.8 μg of the MSS1 expression plasmid, or both using the Lipofectamine 2000 reagent (Invitrogen). At 48 h post-transfection, β-galactosidase activity was determined using the Tropix Gal-Screen assay kit (Applied Biosystems, Carsbad, CA).

## Competing interests

The authors declare that they have no competing interests.

## Authors' contributions

STS carried out the bulk of the technical studies and was the driving force in getting the studies performed. PB was involved in guiding STS during the period of the studies, was the principal investigator of the NIH grant that supported these studies, and helped edit the manuscript. BS was instrumental in identifying the potential functional role of select host proteins that were identified and DR helped establish the functional assay for MSS-1. SB was a student in STS lab and helped in the compiling and analysis of the data presented in the manuscript. AEM helped in the analysis of the data and editing the manuscript. AAA helped in the analysis of the data, overall guidance of the studies and was the individual who wrote and edited the manuscript.

## Supplementary Material

Additional file  1 **Master list of all host proteins identified**. A list of a total of 1979 host proteins found in virus preparations from any one of the rhesus macaques and any one of the sooty mangabeys. Click here for file

Additional file  2 **A list of host proteins common to virus preparations from rhesus macaques and sooty mangabeys**. A list of 202 host proteins that were found in virus preparations of CD4^+ ^T cells from both rhesus macaques and sooty mangabeys. Click here for file

Additional file  3 **A list of proteins found in common between our database and those from Chertova *et al*. **[19]. A list of host proteins that were identified in virus preparations from rhesus macaques and sooty mangabeys and also by the studies of Chertova *et al*. [19]. Click here for file

Additional file  4 **A list of proteins found in common between our database and those from Brass *et al*. **[26]. A list of host proteins that were identified in virus preparations from rhesus macaques and sooty mangabeys and also by the studies of Brass et al [26]. Click here for file

Additional file  5 **A list of proteins found in common between our database and those from Gautier *et al*.**[30]. A list of host proteins that were identified in virus preparations from rhesus macaques and sooty mangabeys and also by the studies of Gautier et al [30]. Click here for file

Additional file  6 **A list of proteins found in common between our database and those from Yeung *et al***. A list of host proteins that were identified in virus preparations from rhesus macaques and sooty mangabeys and also by the studies of Yeung *et al*. [29]. Click here for file

Additional file  7 **Representative Electron micrographs of virus preparations prior to and post purification**. Aliquots of virus preparations prior to enrichment and following purification/enrichment were pelleted and the pellets fixed in glutaraldehyde and prepared for thin section electron micrography. Particles were visualized at 90,000 × and a representative micrograph prior to (A) and post enrichment (B) is displayed. Click here for file
