# Supplementary material for: Distinct host cell proteins incorporated by SIV replicating in CD4+ T Cells from natural disease resistant versus non-natural disease susceptible hosts
Source: Retrovirology. 2010 Dec 16;7:107. doi: 10.1186/1742-4690-7-107 (PMC3012658; doi:10.1186/1742-4690-7-107)
Supplement: Additional file 1 — Master list of all host proteins identified. A list of a total of 1979 host proteins found in virus preparations from any one of the rhesus macaques and any one of the sooty mangabeys. [file 1742-4690-7-107-S1.DOC]

**Addional file I: List of all proteins found**

|  | **Protein Name** | | | **Reference** |
| --- | --- | --- | --- | --- |
| 1 | 14-3-3 protein beta/alpha | | | sp|A4K2U9|1433B_PONAB |
| 2 | 14-3-3 protein epsilon (14-3-3E) (Mitochondrial import stimulation factor L subunit) (MSF L), partial | | | XP_001115237.1 |
| 3 | 14-3-3 protein zeta/delta (Protein kinase C inhibitor protein 1) (KCIP-1) | | | XP_001111077.1 |
| 4 | 1-acylglycerol-3-phosphate O-acyltransferase 5 | | | XP_001098129.1 |
| 5 | 1D-myo-inositol-trisphosphate 3-kinase A | | | XP_001099176.1 |
| 6 | 2,3-bisphosphoglycerate mutase isoform 3 | | | XP_001102783.1 |
| 7 | 2',3'-cyclic nucleotide 3' phosphodiesterase | | | NP_001030605.1 |
| 8 | 2,4-dienoyl CoA reductase 1 | | | XP_001085155.1 |
| 9 | 2-5-oligoadenylate synthetase 2 isoform 2 isoform 2 | | | XP_001111022.1 |
| 10 | 26S protease regulatory subunit 7 (MSS1 protein), partial | | | XP_001118305.1 |
| 11 | 26S protease regulatory subunit 7-like protein | | | tr|A6MLC0|A6MLC0_CALJA |
| 12 | 26S protease regulatory subunit S10B-like protein | | | tr|A6MK92|A6MK92_CALJA |
| 13 | 26S proteasome non-ATPase regulatory subunit 12 (26S proteasome regulatory subunit p55) | | | XP_001082324.1 |
| 14 | 26S proteasome non-ATPase regulatory subunit 12-like protein | | | tr|A6ML21|A6ML21_CALJA |
| 15 | 26S proteasome non-ATPase regulatory subunit 13 (26S proteasome regulatory subunit S11), partial | | | XP_001094583.1 |
| 16 | 26S proteasome non-ATPase regulatory subunit 13-like protein | | | tr|A6MKV9|A6MKV9_CALJA |
| 17 | 2-deoxyribose-5-phosphate aldolase homolog | | | XP_001092767.1 |
| 18 | 3,2-trans-enoyl-CoA isomerase, mitochondrial precursor (Dodecenoyl-CoA isomerase) isoform 2 | | | XP_001084889.1 |
| 19 | 3-hydroxy-3-methylglutaryl-Coenzyme A reductase isoform 5 | | | XP_001104607.1 |
| 20 | 3-hydroxy-3-methylglutaryl-Coenzyme A synthase 1 (soluble) | | | XP_001090857.1 |
| 21 | 3-hydroxyanthranilate 3,4-dioxygenase | | | XP_001111024.1 |
| 22 | 3-mercaptopyruvate sulfurtransferase | | | XP_001084731.1 |
| 23 | 40S ribosomal protein S10 | | | XP_001093979.1 |
| 24 | 40S ribosomal protein S16 | | | XP_001096607.1 |
| 25 | 40S ribosomal protein S17 isoform 1 | | | XP_001115626.1 |
| 26 | 40S ribosomal protein S2, partial | | | XP_001119640.1 |
| 27 | 40S ribosomal protein S2 | | | XP_001114889.1 |
| 28 | 40S ribosomal protein S20 isoform 2 | | | XP_001101768.1 |
| 29 | 40S ribosomal protein S20 | | | XP_001086172.1 |
| 30 | 40S ribosomal protein S25 isoform 3 | | | XP_001115490.1 |
| 31 | 40S ribosomal protein S26 | | | XP_001112565.1 |
| 32 | 40S ribosomal protein S3a (V-fos transformation effector protein), partial | | | XP_001110909.1 |
| 33 | 40S ribosomal protein S4, X isoform isoform 2 | | | XP_001083393.1 |
| 34 | 40S ribosomal protein S4, X isoform | | | XP_001094914.1 |
| 35 | 40S ribosomal protein S6 | | | XP_001111414.1 |
| 36 | 40S ribosomal protein S7 (S8) | | | XP_001095908.1 |
| 37 | 40S ribosomal protein SA (p40) (34/67 kDa laminin receptor) | | | XP_001083829.1 |
| 38 | 40S ribosomal protein SA (p40) (34/67 kDa laminin receptor) isoform 1 | | | XP_001097457.1 |
| 39 | 40S ribosomal protein SA-like protein | | | tr|A6MKK2|A6MKK2_CALJA |
| 40 | 5,10-methylenetetrahydrofolate reductase isoform 3 | | | XP_001105188.1 |
| 41 | 5-hydroxytryptamine (serotonin) receptor 2B isoform 2 | | | XP_001113095.1 |
| 42 | 5-hydroxytryptamine receptor 5A | | | XP_001105989.1 |
| 43 | 5'-nucleotidase, cytosolic II isoform 3 | | | XP_001113578.1 |
| 44 | 5-nucleotidase, cytosolic III isoform 1 isoform 4 | | | XP_001107648.1 |
| 45 | 5T4 oncofetal trophoblast glycoprotein | | | XP_001082914.1 |
| 46 | 60kD Ro/SSA autoantigen, partial | | | XP_001116151.1 |
| 47 | 60kD Ro/SSA autoantigen | | | XP_001105371.1 |
| 48 | 60S acidic ribosomal protein P0 (L10E), partial | | | XP_001115939.1 |
| 49 | 60S acidic ribosomal protein P1 isoform 2 | | | XP_001106053.1 |
| 50 | 60S acidic ribosomal protein P2 (NY-REN-44 antigen) | | | XP_001094442.1 |
| 51 | 60S ribosomal protein L10 (QM protein) (Tumor suppressor QM) (Laminin receptor homolog) | | | XP_001097130.1 |
| 52 | 60S ribosomal protein L12, partial | | | XP_001119378.1 |
| 53 | 60S ribosomal protein L12 | | | XP_001114984.1 |
| 54 | 60S ribosomal protein L14 (CAG-ISL 7), partial | | | XP_001115863.1 |
| 55 | 60S ribosomal protein L17 (L23) | | | XP_001110889.1 |
| 56 | 60S ribosomal protein L22 (Heparin-binding protein HBp15) | | | XP_001098527.1 |
| 57 | 60S ribosomal protein L22 Isoform 6 (Heparin-binding protein HBp15) | | | XP_001091945.1 |
| 58 | 60S ribosomal protein L23 isoform 1 | | | XP_001084513.1 |
| 59 | 60S ribosomal protein L26 (Silica-induced gene 20 protein) (SIG-20) | | | XP_001089113.1 |
| 60 | 60S ribosomal protein L29 (Cell surface heparin-binding protein HIP) | | | XP_001113693.1 |
| 61 | 60S ribosomal protein L32 isoform 2 | | | XP_001117021.1 |
| 62 | 60S ribosomal protein L32 | | | XP_001106882.1 |
| 63 | 60S ribosomal protein L38 | | | XP_001089804.1 |
| 64 | 60S ribosomal protein L6 (TAX-responsive enhancer element-binding protein 107) | | | XP_001110461.1 |
| 65 | 60S ribosomal protein L7 | | | XP_001089573.1 |
| 66 | 60S ribosomal protein L7a | | | XP_001118392.1 |
| 67 | 60S ribosomal protein L8 isoform 2 | | | XP_001099258.1 |
| 68 | 78 kDa glucose-regulated protein precursor (GRP 78), partial | | | XP_001117657.1 |
| 69 | A kinase (PRKA) anchor protein 2 | | | XP_001106950.1 |
| 70 | acetoacetyl-CoA synthetase | | | XP_001103579.1 |
| 71 | acetyl-Coenzyme A acetyltransferase 2 isoform 2 | | | XP_001106967.1 |
| 72 | acidic (leucine-rich) nuclear phosphoprotein 32 family, member A | | | XP_001084552.1 |
| 73 | acidic (leucine-rich) nuclear phosphoprotein 32 family, member B isoform 3 | | | XP_001113699.1 |
| 74 | acidic (leucine-rich) nuclear phosphoprotein 32 family, member E | | | XP_001098977.1 |
| 75 | acidic ribosomal phosphoprotein P0 | | | XP_001087189.1 |
| 76 | aconitase 1 isoform 2 | | | XP_001103761.1 |
| 77 | aconitase 2 precursor isoform 5 | | | XP_001105023.1 |
| 78 | Actin alpha 4-like protein | | | tr|A6ML72|A6ML72_CALJA |
| 79 | Actin beta subunit | | | tr|Q6UIS2|Q6UIS2_PANTR |
| 80 | actin related protein 2/3 complex subunit 1B | | | XP_001111652.1 |
| 81 | actin related protein 2/3 complex subunit 2 | | | XP_001088889.1 |
| 82 | actin related protein 2/3 complex, subunit 3, 21kDa isoform 1 | | | XP_001107069.1 |
| 83 | actin related protein 2/3 complex, subunit 5-like | | | XP_001082363.1 |
| 84 | Actin, alpha cardiac (Alpha-cardiac actin) isoform 3 | | | XP_001088409.1 |
| 85 | Actin, cytoplasmic 2 (Gamma-actin) isoform 1 | | | XP_001110688.1 |
| 86 | actinin alpha 4 isoform 5 | | | XP_001083825.1 |
| 87 | actin-like 6A | | | NP_001098029.1 |
| 88 | actin-related protein 2 isoform 4 | | | XP_001090768.1 |
| 89 | actin-related protein 8 | | | XP_001082127.1 |
| 90 | Activated RNA polymerase II transcriptional coactivator p15 (SUB1 homolog) (PC4) (p14) | | | XP_001089347.1 |
| 91 | activating signal cointegrator 1 complex subunit 3-like 1 isoform 2 | | | XP_001098299.1 |
| 92 | activin A receptor, type IC isoform 4 | | | XP_001088578.1 |
| 93 | Acylamino acid-releasing enzyme-like protein1 | | | tr|A6ML46|A6ML46_CALJA |
| 94 | acyl-CoA synthetase short-chain family member 1 isoform 2 | | | XP_001099300.1 |
| 95 | acyl-CoA thioesterase 7 isoform 3 | | | XP_001092412.1 |
| 96 | ADAM metallopeptidase domain 10 isoform 5 | | | XP_001097016.1 |
| 97 | ADAM metallopeptidase domain 22 isoform 2 preproprotein | | | XP_001104527.1 |
| 98 | ADAM metallopeptidase domain 29 preproprotein | | | XP_001088304.1 |
| 99 | ADAM metallopeptidase with thrombospondin type 1 motif, 13 isoform 1 preproprotein | | | XP_001118382.1 |
| 100 | adaptor-related protein complex 1 beta 1 subunit isoform 7 | | | XP_001106103.1 |
| 101 | adaptor-related protein complex 1, beta 1 subunit isoform 6 | | | XP_001106034.1 |
| 102 | adaptor-related protein complex 2, alpha 1 subunit isoform 1 isoform 3 | | | XP_001115458.1 |
| 103 | adaptor-related protein complex 2, mu 1 subunit isoform 11 | | | XP_001102601.1 |
| 104 | adaptor-related protein complex 2, mu 1 subunit isoform b isoform 8 | | | XP_001102323.1 |
| 105 | adaptor-related protein complex 2, sigma 1 | | | XP_001112688.1 |
| 106 | ADE2-like protein | | | tr|A6MK48|A6MK48_CALJA |
| 107 | adenine phosphoribosyltransferase isoform 2 | | | XP_001089867.1 |
| 108 | adenomatosis polyposis coli 2 isoform 2 | | | XP_001095330.1 |
| 109 | Adenosine deaminase CG11994-PA | | | XP_001106456.1 |
| 110 | adenosine kinase isoform a isoform 2 | | | XP_001098345.1 |
| 111 | Adenosine kinase-like protein | | | tr|A6MKE5|A6MKE5_CALJA |
| 112 | adenosine monophosphate deaminase 1 (isoform M) isoform 2 | | | XP_001111615.1 |
| 113 | adenylosuccinate lyase, partial | | | XP_001117131.1 |
| 114 | adenylosuccinate synthase-like 1 isoform 1 | | | XP_001094957.1 |
| 115 | adenylyl cyclase-associated protein | | | XP_001082428.1 |
| 116 | adipose differentiation-related protein isoform 2 | | | XP_001110125.1 |
| 117 | ADP-ribosylation factor 1 isoform 2 | | | XP_001106772.1 |
| 118 | ADP-ribosylation factor 3 | | | XP_001104802.1 |
| 119 | ADP-ribosylation factor 4 isoform 1 | | | XP_001103974.1 |
| 120 | ADP-ribosylation factor domain protein 1 isoform beta | | | XP_001085950.1 |
| 121 | ADP-ribosylation factor-like 1 | | | XP_001084102.1 |
| 122 | ADP-ribosylation factor-like 6 interacting protein | | | XP_001082004.1 |
| 123 | ADP-ribosylation factor-like protein 2 | | | XP_001118213.1 |
| 124 | ADP-ribosylation-like factor 6 interacting protein 5 isoform 2 | | | XP_001087809.1 |
| 125 | ADP-ribosylhydrolase like 2 | | | XP_001110060.1 |
| 126 | AFG3 ATPase family gene 3-like 2 isoform 2 | | | XP_001094146.1 |
| 127 | agrin | | | XP_001088755.1 |
| 128 | albumin | | | XP_001103956.1 |
| 129 | Alcohol dehydrogenase-like protein | | | tr|A6ML34|A6ML34_CALJA |
| 130 | aldehyde dehydrogenase 1 family, member L1 | | | XP_001108084.1 |
| 131 | aldehyde dehydrogenase 1 family, member L2 | | | XP_001089566.1 |
| 132 | aldehyde dehydrogenase 1A1 isoform 5 | | | XP_001097512.1 |
| 133 | aldehyde dehydrogenase 6A1 precursor isoform 2 | | | XP_001093276.1 |
| 134 | aldehyde dehydrogenase 8A1 isoform 1 | | | XP_001101362.1 |
| 135 | aldehyde dehydrogenase 9A1, partial | | | XP_001112603.1 |
| 136 | Aldo-keto reductase family 1 member C1 | | | tr|Q0R409|Q0R409_MACFA |
| 137 | aldo-keto reductase family 1, member A1 isoform 2 | | | XP_001082982.1 |
| 138 | aldo-keto reductase family 1, member B1 | | | XP_001100679.1 |
| 139 | aldo-keto reductase family 1, member B10 isoform 1 | | | XP_001102426.1 |
| 140 | aldo-keto reductase family 1, member B10, partial | | | XP_001102376.1 |
| 141 | aldo-keto reductase family 1, member C3 (3-alpha hydroxysteroid dehydrogenase, type II) isoform 2 | | | XP_001104543.1 |
| 142 | aldo-keto reductase family 1, member C4, partial | | | XP_001118631.1 |
| 143 | aldo-keto reductase family 1, member D1 | | | XP_001107157.1 |
| 144 | aldo-keto reductase family 7, member A2 | | | XP_001092177.1 |
| 145 | aldolase A isoform 3 | | | XP_001108059.1 |
| 146 | Aldose reductase-like protein (Fragment) | | | tr|A6ML39|A6ML39_CALJA |
| 147 | ALEX3 protein | | | XP_001092863.1 |
| 148 | all-trans-13,14-dihydroretinol saturase | | | XP_001083549.1 |
| 149 | ALMS1 isoform 2 | | | XP_001105121.1 |
| 150 | alpha 1 type IX collagen isoform 1 precursor isoform 2 | | | XP_001111640.1 |
| 151 | alpha 1 type XVIII collagen isoform 1 precursor, partial | | | XP_001118124.1 |
| 152 | alpha 1,2-mannosidase isoform 2 | | | XP_001091256.1 |
| 153 | alpha 2 globin | | | XP_001094404.1 |
| 154 | alpha 2 type I collagen isoform 3 | | | XP_001097831.1 |
| 155 | alpha 2 type VI collagen isoform 2C2 precursor isoform 2 | | | XP_001099019.1 |
| 156 | alpha 3 type VI collagen isoform 1 precursor | | | XP_001113364.1 |
| 157 | alpha 3 type VI collagen isoform 5 precursor isoform 5 | | | XP_001084624.1 |
| 158 | alpha isoform of regulatory subunit A, protein phosphatase 2 isoform 8 | | | XP_001116592.1 |
| 159 | Alpha-2C adrenergic receptor (Alpha-2C adrenoceptor) | | | XP_001114432.1 |
| 160 | alpha-2-HS-glycoprotein isoform 2 | | | XP_001091623.1 |
| 161 | alpha-2-macroglobulin isoform 3 | | | XP_001114328.1 |
| 162 | Alpha-2-macroglobulin precursor (Alpha-2-M) | | | XP_001118453.1 |
| 163 | Alpha-actinin-1 (Alpha-actinin cytoskeletal isoform), partial | | | XP_001114663.1 |
| 164 | alpha-fetoprotein | | | XP_001103873.1 |
| 165 | alveolar soft part sarcoma chromosome region, candidate 1 | | | XP_001112862.1 |
| 166 | AMP-activated protein kinase, noncatalytic gamma-1 subunit isoform 5 | | | XP_001105687.1 |
| 167 | amyloid beta (A4) precursor-like protein 2 | | | XP_001112057.1 |
| 168 | Amyloid beta A4 | | | sp|P53601|A4_MACFA |
| 169 | amyloid beta A4 protein precursor, isoform b isoform 5 | | | XP_001104679.1 |
| 170 | amyotrophic lateral sclerosis 2 (juvenile) chromosome region, candidate 13 | | | XP_001093515.1 |
| 171 | Androgen receptor | | | sp|O97960|ANDR_PAPHA |
| 172 | angiomotin | | | XP_001101620.1 |
| 173 | angiopoietin-like 3 | | | XP_001086114.1 |
| 174 | ankyrin 1 isoform 1 | | | XP_001099591.1 |
| 175 | ankyrin repeat and FYVE domain containing 1 isoform 1 | | | XP_001093340.1 |
| 176 | ankyrin repeat domain 28 isoform 5 | | | XP_001088579.1 |
| 177 | annexin 5 | | | XP_001100224.1 |
| 178 | annexin A11 | | | XP_001096044.1 |
| 179 | annexin A2 isoform 1 | | | XP_001094593.1 |
| 180 | annexin A3 isoform 2 | | | XP_001091995.1 |
| 181 | Annexin A3-like protein | | | tr|A6MKH1|A6MKH1_CALJA |
| 182 | annexin A8 | | | XP_001083294.1 |
| 183 | annexin I isoform 4 | | | XP_001098693.1 |
| 184 | annexin IV isoform 3 | | | XP_001097807.1 |
| 185 | annexin VI | | | XP_001100437.1 |
| 186 | annexin VII isoform 1 isoform 2 | | | XP_001102445.1 |
| 187 | AP-2 complex subunit mu 1-like protein | | | tr|A6MK86|A6MK86_CALJA |
| 188 | APEX nuclease isoform 6 | | | XP_001090006.1 |
| 189 | APG16L beta isoform | | | XP_001110048.1 |
| 190 | Apg3p | | | XP_001104361.1 |
| 191 | APG7 autophagy 7-like isoform 4 | | | XP_001088170.1 |
| 192 | aplysia ras-related homolog A2, partial | | | XP_001115018.1 |
| 193 | APOBEC3H | | | tr|Q1WBT3|Q1WBT3_CERTO |
| 194 | Apolipoprotein A-I precursor (Apo-AI) (ApoA-I) | | | XP_001090535.1 |
| 195 | Apolipoprotein B | | | tr|Q866M8|Q866M8_CALMO |
| 196 | apolipoprotein B isoform 2 | | | XP_001097500.1 |
| 197 | Apolipoprotein D | | | sp|Q8SPI0|APOD_MACFA |
| 198 | Apolipoprotein D precursor (Apo-D) (ApoD) | | | XP_001098104.1 |
| 199 | apolipoprotein E | | | XP_001104482.1 |
| 200 | apolipoprotein L2 | | | XP_001083469.1 |
| 201 | apoptotic chromatin condensation inducer 1 isoform 6 | | | XP_001106207.1 |
| 202 | apoptotic peptidase activating factor isoform 5 | | | XP_001087188.1 |
| 203 | arachidonate 15-lipoxygenase | | | XP_001094627.1 |
| 204 | archaemetzincin-1 | | | XP_001087225.1 |
| 205 | archain isoform 2 | | | XP_001098493.1 |
| 206 | ARG99 homolog | | | XP_001101625.1 |
| 207 | arginase, type I | | | XP_001103609.1 |
| 208 | Arginine N-methyltransferase 1-like protein | | | tr|A6MJY8|A6MJY8_CALJA |
| 209 | argininosuccinate lyase isoform 2 isoform 3 | | | XP_001087106.1 |
| 210 | argininosuccinate synthetase isoform 3 | | | XP_001094196.1 |
| 211 | argininosuccinate synthetase | | | XP_001106949.1 |
| 212 | arginyl-tRNA synthetase | | | XP_001091294.1 |
| 213 | ARHGAP15 | | | XP_001092308.1 |
| 214 | ARP1 actin-related protein 1 homolog B, centractin beta isoform 3 | | | XP_001101028.1 |
| 215 | arrestin domain containing 1, partial | | | XP_001116997.1 |
| 216 | aryl hydrocarbon receptor nuclear translocator-like isoform 2 | | | XP_001095089.1 |
| 217 | arylsulfatase D isoform a precursor, partial | | | XP_001092405.1 |
| 218 | asparagine synthetase isoform 4 | | | XP_001089182.1 |
| 219 | asparagine-linked glycosylation 12, partial | | | XP_001117738.1 |
| 220 | aspartyl-tRNA synthetase | | | XP_001095858.1 |
| 221 | Asporin precursor (Periodontal ligament-associated protein 1) (PLAP-1) | | | XP_001107422.1 |
| 222 | ataxin 2 related protein isoform F | | | XP_001104562.1 |
| 223 | ATP citrate lyase isoform 1 isoform 2 | | | XP_001108114.1 |
| 224 | ATP synthase, H+ transporting, mitochondrial F1 complex, beta subunit | | | XP_001091520.1 |
| 225 | ATPase family, AAA domain containing 3A | | | XP_001103241.1 |
| 226 | ATPase type 13A5 | | | XP_001095224.1 |
| 227 | ATPase, Class I, type 8B, member 2 isoform 2 | | | XP_001114383.1 |
| 228 | ATPase, H+ transporting, lysosomal 42kDa, V1 subunit C1 isoform 2 | | | XP_001083973.1 |
| 229 | ATPase, H+ transporting, lysosomal 70kD, V1 subunit A, isoform 1 | | | XP_001106889.1 |
| 230 | ATPase, H+ transporting, lysosomal, V0 subunit d1 | | | XP_001091627.1 |
| 231 | ATP-binding cassette, sub-family A, member 6 isoform 2 | | | XP_001083245.1 |
| 232 | ATP-binding cassette, sub-family C, member 11 isoform a isoform 4 | | | XP_001114239.1 |
| 233 | ATP-binding cassette, sub-family F, member 1 | | | NP_001098632.1 |
| 234 | ATP-dependent DNA helicase 2 subunit 1-like protein | | | tr|A6MJV9|A6MJV9_CALJA |
| 235 | ATP-dependent DNA helicase II, 70 kDa subunit | | | XP_001105684.1 |
| 236 | ATP-dependent DNA helicase II | | | XP_001084586.1 |
| 237 | ATP-dependent RNA helicase DDX1-like protein | | | tr|A6MKX1|A6MKX1_CALJA |
| 238 | Augmenter of liver regeneration (hERV1 protein) isoform 1 | | | XP_001082384.1 |
| 239 | B5 receptor | | | XP_001084341.1 |
| 240 | barrier to autointegration factor 1 | | | XP_001111924.1 |
| 241 | basic helix-loop-helix domain containing, class B, 3 | | | XP_001098355.1 |
| 242 | basic leucine zipper and W2 domains 2 | | | XP_001104484.1 |
| 243 | basonuclin 1 | | | XP_001111612.1 |
| 244 | B-cell receptor-associated protein 31 | | | XP_001085171.1 |
| 245 | BCL2-interacting killer (apoptosis-inducing) | | | XP_001108040.1 |
| 246 | BCL2-related ovarian killer | | | XP_001093221.1 |
| 247 | BDNF | | | tr|Q71BP2|Q71BP2_9EUTH |
| 248 | Beclin 1 | | | tr|Q3YAP4|Q3YAP4_MACMU |
| 249 | Beta 2-microglobulin | | | tr|Q9TS09|Q9TS09_PAPHA |
| 250 | beta globin | | | XP_001111709.1 |
| 251 | Beta tryptase 2 | | | tr|A8CXJ5|A8CXJ5_PONAB |
| 252 | beta tubulin 1, class VI | | | XP_001082345.1 |
| 253 | beta(1,6)-N-acetylglucosaminyltransferase V isoform 1 isoform 3 | | | XP_001106688.1 |
| 254 | Beta-galactoside-binding lectin | | | tr|B6CPA0|B6CPA0_CERNS |
| 255 | betaine-homocysteine methyltransferase | | | XP_001104072.1 |
| 256 | biglycan | | | XP_001086112.1 |
| 257 | biliverdin reductase A | | | XP_001095668.1 |
| 258 | bin3, bicoid-interacting 3 | | | XP_001104039.1 |
| 259 | blood vessel epicardial substance | | | XP_001087113.1 |
| 260 | bone morphogenetic protein receptor type II isoform 1 | | | XP_001101569.1 |
| 261 | bone morphogenetic protein receptor, type IB isoform 1 | | | XP_001103531.1 |
| 262 | brain and reproductive organ-expressed (TNFRSF1A modulator) isoform 2 isoform 4 | | | XP_001100190.1 |
| 263 | Brain and reproductive organ-expressed protein | | | tr|Q8WN70|Q8WN70_SAGOE |
| 264 | brain creatine kinase | | | XP_001112108.1 |
| 265 | brain glycogen phosphorylase isoform 4 | | | XP_001100555.1 |
| 266 | brain link protein 2 isoform 2 | | | XP_001115151.1 |
| 267 | branched chain aminotransferase 1, cytosolic isoform 2 | | | XP_001101113.1 |
| 268 | BRCA1 associated RING domain 1 isoform 5 | | | XP_001084740.1 |
| 269 | BRCA2 and CDKN1A-interacting protein isoform BCCIPbeta | | | XP_001087888.1 |
| 270 | breast cancer and salivary gland expression | | | NP_001035505.1 |
| 271 | bromodomain adjacent to zinc finger domain, 1B isoform 2 | | | XP_001111145.1 |
| 272 | bromodomain and WD repeat domain containing 1 isoform 1 | | | XP_001108655.1 |
| 273 | bromodomain containing 9 isoform 2 isoform 4 | | | XP_001096110.1 |
| 274 | BTB (POZ) domain containing 11 isoform 3 | | | XP_001093296.1 |
| 275 | BTB/POZ KELCH domain protein | | | XP_001086777.1 |
| 276 | BTG3 associated nuclear protein isoform a isoform 6 | | | XP_001088291.1 |
| 277 | BUB3 budding uninhibited by benzimidazoles 3 isoform a isoform 2 | | | XP_001105443.1 |
| 278 | Bullous pemphigoid antigen 1 isoforms 1/2/3/4/5/8 (Dystonin) | | | XP_001105386.1 |
| 279 | C1q and tumor necrosis factor related protein 2 | | | XP_001084312.1 |
| 280 | C1q and tumor necrosis factor related protein 3 isoform b | | | XP_001090621.1 |
| 281 | C27H2.3 | | | XP_001105528.1 |
| 282 | C32A3.3a | | | XP_001101022.1 |
| 283 | Ca2+-dependent activator protein for secretion 2 isoform b isoform 7 | | | XP_001084413.1 |
| 284 | calcium activated chloride channel 4 | | | XP_001109489.1 |
| 285 | calcium binding protein 39-like isoform 3 | | | XP_001102224.1 |
| 286 | calcium channel, voltage-dependent, alpha 1E subunit | | | XP_001110352.1 |
| 287 | calcium channel, voltage-dependent, alpha 1F subunit | | | XP_001106163.1 |
| 288 | calmegin isoform 4 | | | XP_001089882.1 |
| 289 | calmodulin regulated spectrin-associated protein 1 | | | XP_001118018.1 |
| 290 | calpain 1, large subunit isoform 3 | | | XP_001114109.1 |
| 291 | calpain 2, large subunit | | | XP_001098172.1 |
| 292 | calponin 2 isoform a | | | XP_001117226.1 |
| 293 | calreticulin isoform 2 | | | XP_001110217.1 |
| 294 | calsyntenin 1 | | | XP_001118466.1 |
| 295 | cAMP-dependent protein kinase catalytic subunit alpha | | | XP_001111571.1 |
| 296 | cAMP-dependent protein kinase catalytic subunit beta isoform 11 | | | XP_001106164.1 |
| 297 | cAMP-dependent protein kinase type I-alpha regulatory subunit | | | XP_001112547.1 |
| 298 | cAMP-dependent protein kinase, regulatory subunit beta 2 | | | XP_001089109.1 |
| 299 | cancer susceptibility candidate 4 isoform a | | | XP_001110185.1 |
| 300 | cancer susceptibility candidate 5 isoform 2 | | | XP_001096728.1 |
| 301 | carbohydrate (chondroitin 6) sulfotransferase 3 | | | XP_001106464.1 |
| 302 | carbohydrate (N-acetylglucosamine 6-O) sulfotransferase 5 isoform 2 | | | XP_001110508.1 |
| 303 | carbonyl reductase 1 | | | XP_001088120.1 |
| 304 | carbonyl reductase 3 | | | XP_001084893.1 |
| 305 | carboxypeptidase N, polypeptide 1, 50kD | | | XP_001106976.1 |
| 306 | carboxypeptidase N, polypeptide 2, 83kD | | | XP_001095878.1 |
| 307 | carnitine deficiency-associated, expressed in ventricle 1 isoform 1, partial | | | XP_001116913.1 |
| 308 | cartilage associated protein isoform 4 | | | XP_001099429.1 |
| 309 | cartilage linking protein 1 isoform 1 | | | XP_001112341.1 |
| 310 | cartilage oligomeric matrix protein precursor | | | XP_001115565.1 |
| 311 | Cas-Br-M (murine) ecotropic retroviral transforming sequence | | | XP_001104812.1 |
| 312 | casein kinase 2, alpha prime polypeptide | | | XP_001101458.1 |
| 313 | casein kinase 2, beta subunit isoform 2 | | | XP_001112540.1 |
| 314 | casein kinase II alpha 1 subunit isoform a isoform 3 | | | XP_001112324.1 |
| 315 | CASP3 | | | tr|Q005V2|Q005V2_FELCA |
| 316 | caspase 14, apoptosis-related cysteine peptidase | | | XP_001111598.1 |
| 317 | caspase 3 preproprotein isoform 1 | | | XP_001082922.1 |
| 318 | caspase recruitment domain family, member 4 | | | XP_001085719.1 |
| 319 | catalase isoform 2 | | | XP_001115625.1 |
| 320 | catenin, alpha 1 | | | XP_001113555.1 |
| 321 | cathepsin C isoform 2 | | | XP_001104734.1 |
| 322 | cathepsin D isoform 3 | | | XP_001091601.1 |
| 323 | cathepsin L isoform 1 | | | XP_001085340.1 |
| 324 | caytaxin | | | XP_001117988.1 |
| 325 | C-C chemokine receptor type 5 | | | tr|Q9TQR8|Q9TQR8_9PRIM |
| 326 | CCAAT/enhancer binding protein (C/EBP), gamma | | | XP_001088494.1 |
| 327 | CCR4-NOT transcription complex, subunit 4 | | | XP_001105713.1 |
| 328 | CD109 isoform 2 | | | XP_001112930.1 |
| 329 | CD14 antigen | | | XP_001087242.1 |
| 330 | CD152 | | | tr|Q7JHJ2|Q7JHJ2_CERTO |
| 331 | CD2 antigen (p50), sheep red blood cell receptor | | | XP_001112881.1 |
| 332 | CD209 antigen | | | sp|P60883|CD209_CERAE |
| 333 | CD28 | | | tr|Q9BDN5|Q9BDN5_CERTO |
| 334 | CD3 | | | tr|Q09TK4|Q09TK4_PAPAN |
| 335 | CD38 antigen | | | XP_001099851.1 |
| 336 | CD3d molecule, delta (CD3-TCR complex) | | | XP_001097302.1 |
| 337 | CD3e molecule, epsilon (CD3-TCR complex) | | | XP_001097204.1 |
| 338 | CD4 | | | tr|Q95NE9|Q95NE9_CERPY |
| 339 | CD40 ligand | | | sp|P63305|CD40L_CERTO |
| 340 | CD44 antigen | | | sp|P15379|CD44_MOUSE |
| 341 | CD44 molecule (Indian blood group) isoform 10 | | | XP_001115390.1 |
| 342 | CD45 | | | tr|Q6ED62|Q6ED62_AOTNI |
| 343 | CD53 antigen isoform 2 | | | XP_001102109.1 |
| 344 | CD80 | | | tr|Q9BDN6|Q9BDN6_CERTO |
| 345 | CD81 molecule | | | XP_001093228.1 |
| 346 | CD82 molecule isoform 2 | | | XP_001113618.1 |
| 347 | CD9 molecule isoform 2 | | | XP_001102751.1 |
| 348 | CDC10 cell division cycle 10-like protein | | | tr|Q4G406|Q4G406_MACMU |
| 349 | CDC2-related protein kinase 7 isoform 2 | | | XP_001088594.1 |
| 350 | CDC42-binding protein kinase alpha isoform B | | | XP_001088134.1 |
| 351 | CDC42-binding protein kinase beta, partial | | | XP_001119571.1 |
| 352 | CDC45-like | | | XP_001104872.1 |
| 353 | CDK5 regulatory subunit associated protein 3 isoform b isoform 2 | | | XP_001085224.1 |
| 354 | cDNA sequence BC048546 | | | XP_001118458.1 |
| 355 | cell division cycle 2 protein isoform 3 | | | XP_001095903.1 |
| 356 | cell division cycle 37 homolog (S. cerevisiae)-like 1 | | | XP_001085222.1 |
| 357 | cell division cycle 42 isoform 3 | | | XP_001117013.1 |
| 358 | cell division cycle 42 | | | XP_001106474.1 |
| 359 | cell recognition molecule Caspr2 isoform 2 | | | XP_001094652.1 |
| 360 | cell-cycle and apoptosis regulatory protein 1 | | | XP_001083295.1 |
| 361 | centromere protein E isoform 2 | | | XP_001110550.1 |
| 362 | centromere protein F (350/400kD) | | | XP_001105956.1 |
| 363 | centrosomal protein 290kDa isoform 2 | | | XP_001101114.1 |
| 364 | ceruloplasmin (ferroxidase) isoform 3 | | | XP_001109462.1 |
| 365 | CG10927-PA isoform 1 | | | XP_001096440.1 |
| 366 | CG1332-PA | | | XP_001094456.1 |
| 367 | CG1410-PA, isoform A | | | XP_001099650.1 |
| 368 | CG14446-PA | | | XP_001106655.1 |
| 369 | CG1463-PA | | | XP_001109136.1 |
| 370 | CG15011-PA isoform 4 | | | XP_001102704.1 |
| 371 | CG1550-PA | | | XP_001104848.1 |
| 372 | CG16989-PA | | | XP_001115127.1 |
| 373 | CG18437-PA | | | XP_001102174.1 |
| 374 | CG3104-PA, isoform A | | | XP_001099077.1 |
| 375 | CG31643-PA | | | XP_001091171.1 |
| 376 | CG40449-PA.3 | | | XP_001104993.1 |
| 377 | CG7509-PA | | | XP_001088293.1 |
| 378 | CG8067-PA | | | XP_001083688.1 |
| 379 | CG8399-PA | | | XP_001107575.1 |
| 380 | CG9164-PA, isoform A isoform 1 | | | XP_001101952.1 |
| 381 | CG9581-PA, partial | | | XP_001117185.1 |
| 382 | CG9987-PA | | | XP_001112395.1 |
| 383 | chaperonin containing TCP1, subunit 2 | | | XP_001108460.1 |
| 384 | chaperonin containing TCP1, subunit 3 isoform a isoform 4 | | | XP_001116562.1 |
| 385 | chaperonin containing TCP1, subunit 4 (delta) | | | XP_001114200.1 |
| 386 | chaperonin containing TCP1, subunit 5 (epsilon) isoform 3 | | | XP_001085610.1 |
| 387 | chaperonin containing TCP1, subunit 6A isoform a isoform 3 | | | XP_001090218.1 |
| 388 | chaperonin containing TCP1, subunit 7 isoform 4 | | | XP_001104880.1 |
| 389 | chaperonin containing TCP1, subunit 8 (theta) isoform 5 | | | XP_001101969.1 |
| 390 | chaperonin | | | XP_001086546.1 |
| 391 | chloride intracellular channel 1, partial | | | XP_001118974.1 |
| 392 | chloride intracellular channel 1 | | | XP_001105672.1 |
| 393 | chloride intracellular channel 4 isoform 3 | | | XP_001106485.1 |
| 394 | chloride intracellular channel 6 | | | XP_001103248.1 |
| 395 | chloride ion pump-associated 55 kDa protein isoform 5 | | | XP_001105919.1 |
| 396 | cholinergic receptor, nicotinic, alpha polypeptide 6 | | | XP_001099152.1 |
| 397 | chondroitin sulfate proteoglycan 2 (versican) isoform 8 | | | XP_001112269.1 |
| 398 | Chondrolectin precursor (Transmembrane protein MT75) | | | XP_001106857.1 |
| 399 | chromatin-specific transcription elongation factor large subunit | | | XP_001096507.1 |
| 400 | chromobox homolog 3 | | | XP_001095397.1 |
| 401 | Chromobox protein homolog 5 (Heterochromatin protein 1 homolog alpha) (HP1 alpha) (Antigen p25) | | | XP_001091785.1 |
| 402 | chromogranin A precursor | | | XP_001092629.1 |
| 403 | chromogranin B | | | XP_001112965.1 |
| 404 | chromosome 17 open reading frame 27 | | | XP_001110315.1 |
| 405 | chromosome 2 open reading frame 4 | | | XP_001105478.1 |
| 406 | chromosome 9 open reading frame 19 | | | XP_001082438.1 |
| 407 | citrate synthase precursor, isoform a | | | XP_001098147.1 |
| 408 | CKLF-like MARVEL transmembrane domain containing 6 | | | XP_001098103.1 |
| 409 | class III alcohol dehydrogenase 5 chi subunit isoform 5 | | | XP_001105718.1 |
| 410 | clathrin heavy chain 1 isoform 1 | | | XP_001108373.1 |
| 411 | clathrin heavy chain 1 isoform 2 | | | XP_001108492.1 |
| 412 | clathrin heavy chain 1 isoform 4 | | | XP_001108647.1 |
| 413 | clathrin heavy chain 1 isoform 5 | | | XP_001108702.1 |
| 414 | clathrin, heavy polypeptide-like 1 | | | XP_001112729.1 |
| 415 | claudin 19 isoform 2 | | | XP_001087889.1 |
| 416 | CLIP-170-related protein isoform 2 | | | XP_001101308.1 |
| 417 | CLIP-190 CG5020-PA, isoform A | | | XP_001102815.1 |
| 418 | CD2 | | | tr|Q6SZ59|Q6SZ59_CERTO |
| 419 | CNDP dipeptidase 2 (metallopeptidase M20 family) | | | XP_001084521.1 |
| 420 | coagulation factor II | | | XP_001101815.1 |
| 421 | coagulation factor V precursor | | | XP_001093072.1 |
| 422 | coagulation factor X | | | NP_001098054.1 |
| 423 | coagulation factor XIII A1 subunit isoform 2 | | | XP_001096779.1 |
| 424 | coatomer protein complex subunit alpha, partial | | | XP_001110398.1 |
| 425 | coatomer protein complex, subunit gamma 1, partial | | | XP_001116075.1 |
| 426 | coatomer protein complex, subunit gamma, partial | | | XP_001116067.1 |
| 427 | Coatomer subunit beta (Beta-coat protein) (Beta-COP), partial | | | XP_001104556.1 |
| 428 | Coatomer subunit epsilon-like protein | | | tr|A6MK98|A6MK98_CALJA |
| 429 | Cofilin-1 (Cofilin, non-muscle isoform) (18 kDa phosphoprotein) (p18) | | | XP_001118077.1 |
| 430 | coiled-coil domain containing 40 | | | XP_001109774.1 |
| 431 | coiled-coil domain containing 5 (spindle associated) | | | XP_001084276.1 |
| 432 | coiled-coil domain containing 62 isoform a | | | XP_001095611.1 |
| 433 | coiled-coil domain containing 65 | | | XP_001104499.1 |
| 434 | coiled-coil domain containing 99 isoform 2 | | | XP_001091769.1 |
| 435 | coiled-coil-helix-coiled-coil-helix domain containing 1 isoform 2 | | | XP_001100699.1 |
| 436 | Collagen alpha-1(I) chain precursor | | | XP_001096194.1 |
| 437 | Collagen alpha-1(III) chain precursor | | | XP_001105968.1 |
| 438 | Collagen alpha-1(V) chain precursor, partial | | | XP_001118209.1 |
| 439 | Collagen alpha-1(VI) chain precursor | | | XP_001118050.1 |
| 440 | Collagen alpha-1(XII) chain precursor | | | XP_001109727.1 |
| 441 | collagen and calcium binding EGF domains 1 | | | XP_001090495.1 |
| 442 | collagen, type X, alpha 1 precursor isoform 1 | | | XP_001112083.1 |
| 443 | collagen, type XXIV, alpha 1 isoform 2 | | | XP_001109249.1 |
| 444 | collectin sub-family member 10 | | | XP_001097022.1 |
| 445 | collectin sub-family member 11 isoform a isoform 5 | | | XP_001097594.1 |
| 446 | colony stimulating factor 1 isoform b precursor | | | XP_001090841.1 |
| 447 | Complement C3 precursor | | | XP_001104310.1 |
| 448 | complement component 1 inhibitor, partial | | | XP_001092271.1 |
| 449 | complement component 1, q subcomponent binding protein precursor isoform 3 | | | XP_001100940.1 |
| 450 | complement component 1, s subcomponent isoform 7 | | | XP_001111541.1 |
| 451 | complement component 3, partial | | | XP_001091921.1 |
| 452 | complement component 4A preproprotein, partial | | | XP_001117272.1 |
| 453 | complement component 4B preproprotein, partial | | | XP_001119226.1 |
| 454 | complement component 5 | | | XP_001095750.1 |
| 455 | Complement component 6 | | | XP_001087238.1 |
| 456 | complement component 8, beta polypeptide | | | XP_001114456.1 |
| 457 | complement component 9 | | | XP_001084671.1 |
| 458 | Complement component C3-like protein | | | tr|A6MK21|A6MK21_CALJA |
| 459 | complement factor B isoform 3 | | | XP_001113705.1 |
| 460 | Complement factor I precursor (C3B/C4B inactivator) | | | XP_001087512.1 |
| 461 | COP9 (constitutive photomorphogenic) homolog, subunit 2 isoform 2 | | | XP_001089276.1 |
| 462 | COP9 constitutive photomorphogenic homolog subunit 3 | | | XP_001089496.1 |
| 463 | COP9 constitutive photomorphogenic homolog subunit 7A (Arabidopsis) isoform 7 | | | XP_001108690.1 |
| 464 | COP9 constitutive photomorphogenic homolog subunit 7B isoform 11 | | | XP_001113954.1 |
| 465 | COP9 signalosome complex subunit 1-like protein | | | tr|A6MLC5|A6MLC5_CALJA |
| 466 | COP9 signalosome complex subunit 4 | | | NP_001098750.1 |
| 467 | COP9 signalosome complex subunit 8 (Signalosome subunit 8) | | | XP_001090380.1 |
| 468 | COP9 signalosome subunit 5 isoform 4 | | | XP_001097759.1 |
| 469 | COP9 signalosome subunit 6 | | | XP_001100125.1 |
| 470 | Copine 6-like protein | | | tr|A6MJY0|A6MJY0_CALJA |
| 471 | Copine-7 (Copine VII), partial | | | XP_001117923.1 |
| 472 | coproporphyrinogen oxidase | | | XP_001088605.1 |
| 473 | cordon-bleu homolog | | | XP_001082572.1 |
| 474 | corneodesmosin | | | NP_001098639.1 |
| 475 | coronin 7 | | | XP_001097164.1 |
| 476 | coronin, actin binding protein, 1A | | | XP_001099485.1 |
| 477 | coronin, actin binding protein, 1B | | | XP_001117930.1 |
| 478 | Coronin-7 (70 kDa WD repeat tumor rejection antigen homolog), partial | | | XP_001117248.1 |
| 479 | cryptochrome 2 (photolyase-like) | | | XP_001113162.1 |
| 480 | crystallin, alpha B | | | XP_001106498.1 |
| 481 | crystallin, zeta isoform 3 | | | XP_001100174.1 |
| 482 | CSE1 chromosome segregation 1-like protein isoform 3 | | | XP_001100553.1 |
| 483 | c-src tyrosine kinase isoform 2 | | | XP_001101521.1 |
| 484 | CTP synthase | | | XP_001084608.1 |
| 485 | C-type lectin domain family 11, member A isoform 3 | | | XP_001116136.1 |
| 486 | C-type lectin domain family 4, member g, partial | | | XP_001112388.1 |
| 487 | CUB and Sushi multiple domains 3 isoform 1 isoform 3 | | | XP_001092598.1 |
| 488 | Cullin associated NEDD8-dissociated protein 1-like protein | | | tr|A6ML55|A6ML55_CALJA |
| 489 | cutA divalent cation tolerance homolog (E. coli) isoform 3 | | | XP_001116293.1 |
| 490 | cutaneous T-cell lymphoma tumor antigen se70-2 isoform 2 | | | XP_001100227.1 |
| 491 | CX3CR1 (Fragment) | | | tr|Q6Y3K2|Q6Y3K2_9PRIM |
| 492 | C-X-C chemokine receptor type 4 | | | sp|O62747|CXCR4_CERTO |
| 493 | C-X-C chemokine receptor type 6 | | | sp|Q9N0Z0|CXCR6_CERTO |
| 494 | CXXC finger 5 | | | XP_001084794.1 |
| 495 | Cyclic AMP-responsive element-binding protein 3-like protein | | | tr|A6MLF8|A6MLF8_CALJA |
| 496 | cyclin M3 isoform 1 | | | XP_001098957.1 |
| 497 | cystatin A (stefin A) | | | XP_001112115.1 |
| 498 | cystatin F | | | XP_001098076.1 |
| 499 | Cysteine and glycine-rich protein 1 (Cysteine-rich protein 1) (CRP1) (CRP) | | | XP_001098252.1 |
| 500 | cytochrome b5 reductase | | | XP_001103634.1 |
| 501 | Cytochrome P450 4A11 precursor (CYPIVA11) (Fatty acid omega-hydroxylase) | | | XP_001099576.1 |
| 502 | actin - beta isoform 2 cytoplasmic | | | XP_001110809.1 |
| 503 | actin - beta cytoplasmic | | | XP_001105320.1 |
| 504 | FMR1 interacting protein 1 isoform a - cytoplasmic | | | XP_001113740.1 |
| 505 | acyl coenzyme A thioester hydrolase-like protein - cytosolic | | | tr|A6MJZ1|A6MJZ1_CALJA |
| 506 | damage-specific DNA binding protein 1 | | | XP_001082958.1 |
| 507 | DEAD (Asp-Glu-Ala-Asp) box polypeptide 1 | | | XP_001090643.1 |
| 508 | DEAD (Asp-Glu-Ala-Asp) box polypeptide 39 | | | XP_001112060.1 |
| 509 | DEAD (Asp-Glu-Ala-Asp) box polypeptide 53 | | | XP_001087519.1 |
| 510 | DEAD (Asp-Glu-Ala-Asp) box polypeptide 55 | | | XP_001098453.1 |
| 511 | DEAD box polypeptide 17 isoform p82 | | | XP_001092491.1 |
| 512 | DEAD box polypeptide 42 protein isoform 6 | | | XP_001116390.1 |
| 513 | DEAD/H (Asp-Glu-Ala-Asp/His) box polypeptide 3 | | | XP_001095294.1 |
| 514 | DEAH (Asp-Glu-Ala-His) box polypeptide 15 isoform 5 | | | XP_001106067.1 |
| 515 | DEAH (Asp-Glu-Ala-His) box polypeptide 36 isoform 2 | | | XP_001106315.1 |
| 516 | DEAH (Asp-Glu-Ala-His) box polypeptide 9 isoform 1 | | | XP_001114384.1 |
| 517 | death effector filament-forming Ced-4-like apoptosis protein isoform 1, partial | | | XP_001085766.1 |
| 518 | dehydrogenase/reductase (SDR family) member 7 isoform 3 | | | XP_001095790.1 |
| 519 | deoxycytidine kinase | | | XP_001093411.1 |
| 520 | deoxyhypusine synthase isoform 4 | | | XP_001108116.1 |
| 521 | DEP domain containing 1B | | | XP_001103700.1 |
| 522 | dermcidin preproprotein | | | XP_001092492.1 |
| 523 | desmin isoform 2 | | | XP_001105401.1 |
| 524 | desmoglein 1 | | | XP_001098203.1 |
| 525 | desmoplakin isoform I isoform 2 | | | XP_001085012.1 |
| 526 | Destrin | | | tr|Q3YAN6|Q3YAN6_MACMU |
| 527 | deubiquitinating enzyme 3, partial | | | XP_001119441.1 |
| 528 | developmental pluripotency associated 5 | | | XP_001112340.1 |
| 529 | developmentally regulated GTP binding protein 2 isoform 2 | | | XP_001093568.1 |
| 530 | diacylglycerol kinase, gamma 90kDa isoform 3 | | | XP_001093029.1 |
| 531 | diaphanous 1 | | | XP_001089232.1 |
| 532 | dicarbonyl/L-xylulose reductase | | | XP_001112983.1 |
| 533 | dickkopf homolog 3 isoform 5 | | | XP_001097909.1 |
| 534 | digestive-organ expansion factor | | | XP_001109922.1 |
| 535 | Dihydropteridine reductase (HDHPR) (Quinoid dihydropteridine reductase) isoform 3 | | | XP_001102244.1 |
| 536 | Dihydropteridine reductase-like protein | | | tr|A6MKX9|A6MKX9_CALJA |
| 537 | dihydropyrimidinase | | | XP_001086073.1 |
| 538 | DISC1 | | | tr|A9UK91|A9UK91_MACMU |
| 539 | disintegrin and metalloproteinase | | | XP_001111189.1 |
| 540 | disrupted in schizophrenia 1 isoform L | | | XP_001112728.1 |
| 541 | Disulfide-isomerase A3-like protein | | | tr|A6ML76|A6ML76_CALJA |
| 542 | DNA (cytosine-5-)-methyltransferase 1 | | | XP_001104704.1 |
| 543 | DNA fragmentation factor, 40 kD, beta polypeptide isoform 1 | | | XP_001087149.1 |
| 544 | DNA ligase IV | | | XP_001084107.1 |
| 545 | DNA polymerase | | | tr|O91128|O91128_9HIV2 |
| 546 | DNA topoisomerase I | | | XP_001088021.1 |
| 547 | DNA topoisomerase II, beta isozyme | | | XP_001092092.1 |
| 548 | DNA-crosslink repair gene SNM1 | | | XP_001090942.1 |
| 549 | DNA-directed RNA polymerase II largest subunit (RPB1) | | | XP_001118060.1 |
| 550 | DnaJ (Hsp40) homolog, subfamily A, member 1 isoform 2 | | | XP_001101853.1 |
| 551 | DnaJ (Hsp40) homolog, subfamily C, member 11 | | | XP_001094602.1 |
| 552 | DnaJ (Hsp40) homolog, subfamily C, member 6 isoform 4 | | | XP_001090170.1 |
| 553 | DOT1-like, histone H3 methyltransferase (S. cerevisiae) isoform 2 | | | XP_001097312.1 |
| 554 | dual specificity phosphatase 26 | | | XP_001087847.1 |
| 555 | dynactin 1 isoform 1 isoform 10 | | | XP_001108541.1 |
| 556 | dynactin 2 isoform 6 | | | XP_001116148.1 |
| 557 | dynactin 3 isoform 1 isoform 2 | | | XP_001096432.1 |
| 558 | dynamin 1-like protein isoform 5 | | | XP_001086230.1 |
| 559 | dynamin 2 isoform 10 | | | XP_001103597.1 |
| 560 | Dynamin-1 (D100) (Dynamin, brain) (B-dynamin) | | | XP_001110748.1 |
| 561 | dynein, axonemal, heavy polypeptide 1 | | | XP_001085984.1 |
| 562 | dynein, axonemal, heavy polypeptide 9 | | | XP_001114113.1 |
| 563 | dynein, cytoplasmic, heavy polypeptide 1 | | | XP_001112455.1 |
| 564 | dynein, cytoplasmic, intermediate polypeptide 2 | | | XP_001084340.1 |
| 565 | E2F transcription factor 6 isoform 1 isoform 2 | | | XP_001088811.1 |
| 566 | early growth response 1 | | | XP_001107731.1 |
| 567 | echinoderm microtubule associated protein like 1 isoform b isoform 3 | | | XP_001103968.1 |
| 568 | echinoderm microtubule associated protein like 2 isoform 5 | | | XP_001110965.1 |
| 569 | ectonucleoside triphosphate diphosphohydrolase 6 isoform 3 | | | XP_001100005.1 |
| 570 | ectonucleotide pyrophosphatase/phosphodiesterase 1 | | | XP_001103359.1 |
| 571 | EF hand domain family, member B isoform 4 | | | XP_001088383.1 |
| 572 | EGF-containing fibulin-like extracellular matrix protein 2 | | | XP_001118071.1 |
| 573 | EGF-like domain-containing protein 9-like protein | | | tr|A6MJW0|A6MJW0_CALJA |
| 574 | EGF-like-domain, multiple 9 isoform 2 | | | XP_001094533.1 |
| 575 | Egg-derived tyrosine phosphatase CG6542-PA, isoform A | | | XP_001092278.1 |
| 576 | egl nine homolog 1 isoform 2 | | | XP_001104870.1 |
| 577 | EH-domain containing 1 | | | XP_001118235.1 |
| 578 | EH-domain containing 3 | | | XP_001105262.1 |
| 579 | EH-domain containing 4 | | | XP_001102348.1 |
| 580 | elastin microfibril interfacer 3 | | | XP_001086225.1 |
| 581 | Elongation factor 1 gamma-like protein | | | tr|A6MJV4|A6MJV4_CALJA |
| 582 | elongation factor, RNA polymerase II, 2 isoform 2 | | | XP_001094059.1 |
| 583 | elongin A isoform 1 | | | XP_001103322.1 |
| 584 | EMSY protein | | | XP_001087550.1 |
| 585 | endomembrane protein emp70 precursor isolog isoform 1 | | | XP_001101248.1 |
| 586 | Endoplasmic reticulum lumenal protein ERP28 | | | tr|Q3YAI4|Q3YAI4_MACMU |
| 587 | endothelial PAS domain protein 1 isoform 5 | | | XP_001113007.1 |
| 588 | enolase 1 isoform 8 | | | XP_001098883.1 |
| 589 | enolase 3, partial | | | XP_001083318.1 |
| 590 | Envelope glycoprotein | | | tr|Q6J3P9|Q6J3P9_SIVCZ |
| 591 | Envelope glycoprotein gp105 | | | tr|Q74116|Q74116_9HIV2 |
| 592 | Envelope glycoprotein gp120 | | | tr|Q72951|Q72951_9HIV1 |
| 593 | Envelope glycoprotein gp160 | | | tr|B3GV32|B3GV32_9HIV1 |
| 594 | Envelope glycoprotein gp41 | | | tr|Q9YYY3|Q9YYY3_9HIV1 |
| 595 | envelope glycoprotein, Truncated | | | tr|Q8JDL4|Q8JDL4_9HIV1 |
| 596 | envoplakin | | | XP_001101955.1 |
| 597 | eosinophil chemotactic cytokine | | | XP_001104487.1 |
| 598 | ephrin B2 | | | XP_001100069.1 |
| 599 | ephrin receptor EphB1 isoform 2 | | | XP_001115223.1 |
| 600 | epidermal growth factor receptor pathway substrate 8-like protein 1 isoform a | | | XP_001086805.1 |
| 601 | epiplakin 1 | | | XP_001088100.1 |
| 602 | epoxide hydrolase 2, cytoplasmic | | | XP_001109474.1 |
| 603 | epsilon subunit of coatomer protein complex isoform a isoform 3 | | | XP_001114807.1 |
| 604 | erythrocyte membrane protein band 4.1 like 5 | | | XP_001088764.1 |
| 605 | esterase D/formylglutathione hydrolase | | | XP_001097371.1 |
| 606 | Eukaryotic translation elongation factor 1 | | | tr|Q3YAP9|Q3YAP9_MACMU |
| 607 | eukaryotic translation elongation factor 1 alpha 1 isoform 1 | | | XP_001112479.1 |
| 608 | eukaryotic translation elongation factor 1 alpha 1 isoform 3 | | | XP_001112598.1 |
| 609 | eukaryotic translation elongation factor 1 alpha 1 isoform 4 | | | XP_001112629.1 |
| 610 | eukaryotic translation elongation factor 1 alpha 1 isoform 7 | | | XP_001112724.1 |
| 611 | eukaryotic translation elongation factor 1 alpha 1, partial | | | XP_001118614.1 |
| 612 | eukaryotic translation elongation factor 1 beta 2 isoform 1 | | | XP_001106040.1 |
| 613 | eukaryotic translation elongation factor 1 delta (guanine nucleotide exchange protein) | | | XP_001097290.1 |
| 614 | Eukaryotic translation elongation factor 1 epsilon-1 (Elongation factor p18) | | | XP_001086399.1 |
| 615 | eukaryotic translation elongation factor 1 gamma, partial | | | XP_001119776.1 |
| 616 | eukaryotic translation elongation factor 1 gamma | | | XP_001118440.1 |
| 617 | eukaryotic translation elongation factor 2 | | | XP_001118006.1 |
| 618 | eukaryotic translation initiation factor 2, subunit 1 alpha, 35kDa isoform 2 | | | XP_001105924.1 |
| 619 | eukaryotic translation initiation factor 2, subunit 3 gamma, 52kDa | | | XP_001089902.1 |
| 620 | eukaryotic translation initiation factor 2, subunit 3, structural gene X-linked | | | XP_001093771.1 |
| 621 | eukaryotic translation initiation factor 2B, subunit 5 epsilon, 82kDa isoform 5 | | | XP_001104122.1 |
| 622 | eukaryotic translation initiation factor 2C, 2 | | | XP_001100725.1 |
| 623 | eukaryotic translation initiation factor 3 subunit 6 interacting protein isoform 2 | | | XP_001090065.1 |
| 624 | Eukaryotic translation initiation factor 3 subunit 6-interacting protein-like protein | | | tr|A6MKW4|A6MKW4_CALJA |
| 625 | eukaryotic translation initiation factor 3 subunit 7 isoform 1 | | | XP_001083790.1 |
| 626 | eukaryotic translation initiation factor 3 subunit 7 isoform 2 | | | XP_001084017.1 |
| 627 | eukaryotic translation initiation factor 3, subunit 10 theta, 150/170kDa, partial | | | XP_001118685.1 |
| 628 | eukaryotic translation initiation factor 3, subunit 10 theta, 150/170kDa | | | XP_001102472.1 |
| 629 | eukaryotic translation initiation factor 3, subunit 12 isoform 3 | | | XP_001082632.1 |
| 630 | eukaryotic translation initiation factor 3, subunit 3 gamma, 40kDa isoform 2 | | | XP_001093736.1 |
| 631 | eukaryotic translation initiation factor 3, subunit 5 epsilon, 47kDa isoform 3 | | | XP_001105893.1 |
| 632 | eukaryotic translation initiation factor 3, subunit 6 interacting protein, partial | | | XP_001116978.1 |
| 633 | eukaryotic translation initiation factor 3, subunit 9 eta, 116kDa | | | XP_001086876.1 |
| 634 | eukaryotic translation initiation factor 3 | | | XP_001090244.1 |
| 635 | eukaryotic translation initiation factor 4A, isoform 1 isoform 3 | | | XP_001109752.1 |
| 636 | eukaryotic translation initiation factor 4A2 isoform 4 | | | XP_001102692.1 |
| 637 | Eukaryotic translation initiation factor 4E (eIF4E) (eIF-4E) | | | XP_001104976.1 |
| 638 | eukaryotic translation initiation factor 5 | | | XP_001086107.1 |
| 639 | eukaryotic translation initiation factor 5A, partial | | | XP_001118011.1 |
| 640 | Eukaryotic translation initiation factor 6 (eIF-6) (B4 integrin interactor), partial | | | XP_001098145.1 |
| 641 | eukaryotic translation termination factor 1, partial | | | XP_001114680.1 |
| 642 | eukaryotic translation termination factor 1 isoform 4 | | | XP_001112990.1 |
| 643 | exonuclease 1 isoform b isoform 3 | | | XP_001093542.1 |
| 644 | exosome component 9 isoform 2 | | | XP_001101063.1 |
| 645 | exportin 1 isoform 3 | | | XP_001115623.1 |
| 646 | exportin 4 | | | XP_001085699.1 |
| 647 | extra spindle poles like 1, partial | | | XP_001119353.1 |
| 648 | Extra-membrane envelope protein | | | tr|A0JH49|A0JH49_SIVCZ |
| 649 | F35D11.11b | | | XP_001110829.1 |
| 650 | F-actin capping protein alpha-1 subunit | | | XP_001107677.1 |
| 651 | F-actin capping protein beta subunit, partial | | | XP_001119308.1 |
| 652 | family with sequence similarity 76, member A isoform 4 | | | XP_001111954.1 |
| 653 | fatty acid binding protein 5 (psoriasis-associated) | | | XP_001091773.1 |
| 654 | fatty acid binding protein 7, brain isoform 2 | | | XP_001108559.1 |
| 655 | fatty acid synthase | | | XP_001113076.1 |
| 656 | F-box and leucine-rich repeat protein 11 | | | XP_001107511.1 |
| 657 | F-box and leucine-rich repeat protein 18 | | | XP_001107854.1 |
| 658 | F-box and leucine-rich repeat protein 21 | | | XP_001110935.1 |
| 659 | F-box and leucine-rich repeat protein 9 | | | XP_001087391.1 |
| 660 | F-box and WD-40 domain protein 10 isoform 2 | | | XP_001084872.1 |
| 661 | F-box protein 10 | | | XP_001111119.1 |
| 662 | F-box protein 40 | | | XP_001111331.1 |
| 663 | F-box protein 41 | | | XP_001103806.1 |
| 664 | Fc receptor-like and mucin-like 2 isoform 3 | | | XP_001118137.1 |
| 665 | Ferritin heavy chain (Ferritin H subunit) (Proliferation-inducing gene 15 protein) | | | XP_001104405.1 |
| 666 | Fertilin alpha-II | | | tr|O46652|O46652_PAPAN |
| 667 | fetuin B isoform 3 | | | XP_001091143.1 |
| 668 | fibrillarin | | | XP_001088664.1 |
| 669 | fibrinogen alpha chain isoform 4 | | | XP_001089192.1 |
| 670 | fibrinogen, beta chain isoform 4 | | | XP_001091998.1 |
| 671 | fibroblast growth factor 13 isoform 2 | | | XP_001084216.1 |
| 672 | fibroblast growth factor 14 isoform 2 | | | XP_001093573.1 |
| 673 | fibroblast growth factor receptor 1 isoform 4 precursor | | | XP_001090823.1 |
| 674 | fibromodulin precursor | | | XP_001102658.1 |
| 675 | fibronectin 1 isoform 1 preproprotein | | | XP_001083548.1 |
| 676 | fibulin 1 isoform 2 | | | XP_001109966.1 |
| 677 | fibulin 1 isoform 3 | | | XP_001110011.1 |
| 678 | filaggrin 2 | | | XP_001109011.1 |
| 679 | filaggrin, partial | | | XP_001101725.1 |
| 680 | filamin 1 (actin-binding protein-280) isoform 5 | | | XP_001091203.1 |
| 681 | filamin A interacting protein 1 isoform 4 | | | XP_001113175.1 |
| 682 | filamin B, beta (actin binding protein 278) isoform 3 | | | XP_001097922.1 |
| 683 | Flavin reductase (FR) (NADPH-dependent diaphorase) (NADPH-flavin reductase), partial | | | XP_001091330.1 |
| 684 | flightless I homolog isoform 5 | | | XP_001094751.1 |
| 685 | Flotillin-2 (Reggie-1) (REG-1) | | | XP_001107301.1 |
| 686 | Folate transporter 1 (Solute carrier family 19 member 1) | | | XP_001118114.1 |
| 687 | forkhead box C1 | | | XP_001119000.1 |
| 688 | forkhead box O6 | | | XP_001086081.1 |
| 689 | formin 2 isoform 2 | | | XP_001095731.1 |
| 690 | formin 3 CG33556-PA | | | XP_001094595.1 |
| 691 | Formin-1 isoform IV (Limb deformity protein) | | | XP_001084432.1 |
| 692 | fructosamine-3-kinase-related protein | | | XP_001113598.1 |
| 693 | fumarate hydratase isoform 2 | | | XP_001094968.1 |
| 694 | fumarylacetoacetate hydrolase domain containing 2A | | | XP_001095387.1 |
| 695 | Fzr1 protein | | | XP_001117911.1 |
| 696 | G protein pathway suppressor 1 isoform 1 | | | XP_001113420.1 |
| 697 | G protein-coupled receptor 156 | | | XP_001110586.1 |
| 698 | G protein-coupled receptor 158 | | | XP_001101165.1 |
| 699 | G protein-coupled receptor 37 | | | XP_001087229.1 |
| 700 | G protein-coupled receptor 7 | | | XP_001102797.1 |
| 701 | Gag polyprotein | | | tr|Q5IK81|Q5IK81_9HIV1 |
| 702 | Gag-Pol polyprotein | | | sp|P17283|POL_SIVCZ |
| 703 | galectin 3 binding protein | | | XP_001109047.1 |
| 704 | Galectin-5 (RL-18) | | | XP_001085876.1 |
| 705 | Gamma2-globulin | | | tr|Q9GJY8|Q9GJY8_CALMO |
| 706 | gamma-aminobutyric acid (GABA) A receptor, alpha 5 isoform 1 | | | XP_001109173.1 |
| 707 | gamma-aminobutyric acid (GABA) A receptor, beta 2 isoform 1 isoform 2 | | | XP_001085738.1 |
| 708 | gamma-aminobutyric acid (GABA) receptor, theta precursor | | | XP_001093973.1 |
| 709 | Gamma-aminobutyric-acid receptor beta-1 subunit precursor (GABA(A) receptor) | | | XP_001091167.1 |
| 710 | gamma-glutamyl hydrolase precursor | | | XP_001091897.1 |
| 711 | GAPDH (fragment) | | | tr|Q6IUG3|Q6IUG3_MACMU |
| 712 | GAPDH, spermatogenic | | | XP_001095332.1 |
| 713 | GAPDH | | | XP_001105471.1 |
| 714 | gastric inhibitory polypeptide | | | XP_001091383.1 |
| 715 | GDNF family receptor alpha 3 preproprotein isoform 2 | | | XP_001112630.1 |
| 716 | GDP dissociation inhibitor 2 isoform 5 | | | XP_001105854.1 |
| 717 | GDP-mannose 4,6-dehydratase | | | XP_001089757.1 |
| 718 | GDP-mannose pyrophosphorylase B isoform 2 | | | XP_001106890.1 |
| 719 | gelsolin isoform 19 | | | XP_001093567.1 |
| 720 | gelsolin-like capping protein | | | XP_001084974.1 |
| 721 | general transcription factor IIH, polypeptide 1, 62kDa isoform 1 | | | XP_001085333.1 |
| 722 | gephyrin isoform 2 | | | XP_001105293.1 |
| 723 | G-gamma globulin | | | NP_001040611.1 |
| 724 | GLE1-like, RNA export mediator isoform 1 isoform 2 | | | XP_001110844.1 |
| 725 | GLI-Kruppel family member HKR2 | | | XP_001101733.1 |
| 726 | glucosamine-fructose-6-phosphate aminotransferase | | | XP_001096250.1 |
| 727 | glucose-6-phosphate dehydrogenase isoform 2 | | | XP_001095382.1 |
| 728 | Glucose-6-phosphate isomerase | | | tr|A6MKT0|A6MKT0_CALJA |
| 729 | Glucose-6-phosphate isomerase (GPI) (Phosphoglucose isomerase)(SA-36) | | | XP_001110104.1 |
| 730 | glutamate decarboxylase-like 1 | | | XP_001094833.1 |
| 731 | Glutamate dehydrogenase 1, mitochondrial precursor (GDH) (Memory-related protein 2) (MRG-2) | | | XP_001087050.1 |
| 732 | Glutamate receptor 1 | | | sp|Q38PU8|GRIA1_MACFA |
| 733 | Glutamate receptor, ionotropic, AMPA 1 isoform 9 | | | XP_001111339.1 |
| 734 | glutamate-rich WD repeat containing 1 | | | XP_001113650.1 |
| 735 | glutamine-fructose-6-phosphate transaminase 2 | | | XP_001106226.1 |
| 736 | Glutathione peroxidase 3 | | | sp|Q5RFG3|GPX3_PONPY |
| 737 | Glutathione reductase, mitochondrial precursor (GR) (GRase), partial | | | XP_001114581.1 |
| 738 | Glutathione S-transferase M | | | sp|Q9TSM5|GSTM1_MACFA |
| 739 | glutathione S-transferase M1 isoform 5 | | | XP_001096163.1 |
| 740 | glutathione S-transferase M3 (brain) | | | XP_001096283.1 |
| 741 | glutathione S-transferase P | | | NP_001036141.1 |
| 742 | glutathione S-transferase T | | | XP_001089367.1 |
| 743 | glycine-, glutamate-, thienylcyclohexylpiperidine-binding protein | | | XP_001084851.1 |
| 744 | glycine-N-acyltransferase isoform a isoform 2 | | | XP_001092889.1 |
| 745 | glycogen phosphorylase isoform 2 | | | XP_001114805.1 |
| 746 | glycogen phosphorylase, liver | | | XP_001102253.1 |
| 747 | Glycoprotein H | | | tr|Q806A7|Q806A7_CHV1 |
| 748 | Glycylpeptide N-tetradecanoyltransferase | | | tr|A6MJX2|A6MJX2_CALJA |
| 749 | glycyl-tRNA synthetase | | | XP_001086099.1 |
| 750 | glyoxalase I | | | XP_001117098.1 |
| 751 | glypican 4 | | | XP_001097252.1 |
| 752 | golgi autoantigen, golgin subfamily a, 4 | | | XP_001090210.1 |
| 753 | Golgi coiled-coil protein 1 | | | XP_001089873.1 |
| 754 | golgi phosphoprotein 2 | | | XP_001082060.1 |
| 755 | Golgi SNAP receptor complex member 1 (28 kDa Golgi SNARE protein) | | | XP_001108522.1 |
| 756 | Golgin subfamily A member 8A/B (Golgi autoantigen golgin-67), partial | | | XP_001106862.1 |
| 757 | granule cell antiserum positive 14 isoform 1, partial | | | XP_001095828.1 |
| 758 | granzyme A (granzyme 1, cytotoxic T-lymphocyte-associated serine esterase 3) | | | XP_001097639.1 |
| 759 | granzyme B (granzyme 2, cytotoxic T-lymphocyte-associated serine esterase 1) isoform 3 | | | XP_001114420.1 |
| 760 | Growth/differentiation factor 11 precursor (GDF-11) (Bone morphogenetic protein 11) | | | XP_001096135.1 |
| 761 | G-substrate | | | XP_001083835.1 |
| 762 | GTPase, IMAP family member 4 isoform 2 | | | XP_001098713.1 |
| 763 | GTPase, IMAP family member 6 isoform 3 isoform 1 | | | XP_001098818.1 |
| 764 | GTPase, IMAP family member 7 | | | XP_001098518.1 |
| 765 | GTPase, IMAP family member 8 | | | XP_001098321.1 |
| 766 | GTP-binding protein 9-like protein | | | tr|A6ML78|A6ML78_CALJA |
| 767 | GTP-binding protein PTD004 isoform 6 | | | XP_001088356.1 |
| 768 | guanine monophosphate synthetase | | | XP_001105046.1 |
| 769 | guanine nucleotide binding protein (G protein), alpha 13 | | | XP_001110448.1 |
| 770 | guanine nucleotide binding protein (G protein), alpha inhibiting activity polypeptide 2 isoform 1 | | | XP_001103040.1 |
| 771 | guanine nucleotide binding protein, alpha transducing activity polypeptide 1 | | | XP_001103202.1 |
| 772 | guanine nucleotide binding protein-like 3 (nucleolar)-like isoform 2 | | | XP_001090251.1 |
| 773 | guanine nucleotide binding protein-like 3 isoform 1 | | | XP_001086259.1 |
| 774 | Guanine nucleotide-binding protein | | | tr|Q6UIQ2|Q6UIQ2_PANTR |
| 775 | Guanine nucleotide-binding protein beta subunit 2-like 1 (RACK1) isoform 4 | | | XP_001105066.1 |
| 776 | guanine nucleotide-binding protein, beta 2 | | | XP_001113183.1 |
| 777 | guanylate binding protein family, member 6 | | | XP_001086706.1 |
| 778 | haloacid dehalogenase-like hydrolase domain containing 2 | | | XP_001088800.1 |
| 779 | Haptoglobin | | | tr|Q5VAN2|Q5VAN2_CERTO |
| 780 | haptoglobin isoform 2 | | | XP_001103863.1 |
| 781 | HBS1-like isoform 3 | | | XP_001100221.1 |
| 782 | heat shock 10kDa protein 1 (chaperonin 10) | | | XP_001091673.1 |
| 783 | heat shock 70 protein 1B | | | XP_001115060.1 |
| 784 | heat shock 70kDa protein 1-like isoform 2 | | | XP_001113329.1 |
| 785 | heat shock 70kDa protein 4 isoform a isoform 3 | | | XP_001106968.1 |
| 786 | heat shock 70kDa protein 5 (glucose-regulated protein, 78kDa) isoform 1 | | | XP_001098999.1 |
| 787 | heat shock protein 12A | | | XP_001095628.1 |
| 788 | heat shock protein 60 (mitochondrial)-like protein | | | tr|A6MK13|A6MK13_CALJA |
| 789 | heat shock protein 70 protein binding protein | | | XP_001102391.1 |
| 790 | heat shock protein 70kDa protein 1-like | | | tr|A6MKF9|A6MKF9_CALJA |
| 791 | Heat shock protein 90 protein 1, alpha (Fragment) | | | tr|Q3YAP6|Q3YAP6_MACMU |
| 792 | Heat shock protein 90 protein 1, beta | | | tr|Q3YAN8|Q3YAN8_MACMU |
| 793 | heat shock protein 90 protein 1, beta isoform 10 | | | XP_001099439.1 |
| 794 | Heat shock transcription factor, Y-linked (Heat shock transcription factor 2-like protein) (HSF2-like) | | | XP_001089561.1 |
| 795 | hect domain and RLD 2 | | | XP_001109429.1 |
| 796 | hect domain and RLD 3 | | | XP_001100859.1 |
| 797 | HECT, UBA and WWE domain containing 1 | | | XP_001088987.1 |
| 798 | hedgehog acyltransferase isoform 1 | | | XP_001109407.1 |
| 799 | hedgehog-interacting protein | | | XP_001093950.1 |
| 800 | Hemoglobin | | | sp|P01933|HBA_CERTO |
| 801 | hemojuvelin isoform b isoform 3 | | | XP_001093203.1 |
| 802 | hemopexin | | | XP_001109797.1 |
| 803 | heparan sulfate proteoglycan 2 | | | XP_001099299.1 |
| 804 | heparin cofactor II isoform 2 | | | XP_001086464.1 |
| 805 | HERV-K_12q14.1 provirus ancestral Env polyprotein | | | sp|P61565|ENK1_HUMAN |
| 806 | heterogeneous nuclear ribonucleoprotein A1 | | | XP_001109413.1 |
| 807 | heterogeneous nuclear ribonucleoprotein C (C1/C2) isoform 8 | | | XP_001096688.1 |
| 808 | heterogeneous nuclear ribonucleoprotein C isoform b | | | XP_001109022.1 |
| 809 | Heterogeneous nuclear ribonucleoprotein C-like 1 (hnRNP core protein C-like 1) isoform 2 | | | XP_001106484.1 |
| 810 | Heterogeneous nuclear ribonucleoprotein D0 (hnRNP D0) isoform 1 | | | XP_001088727.1 |
| 811 | heterogeneous nuclear ribonucleoprotein H1 isoform 8 | | | XP_001100049.1 |
| 812 | heterogeneous nuclear ribonucleoprotein K isoform a isoform 13 | | | XP_001105897.1 |
| 813 | heterogeneous nuclear ribonucleoprotein K isoform a isoform 9 | | | XP_001105628.1 |
| 814 | heterogeneous nuclear ribonucleoprotein L isoform a isoform 3 | | | XP_001085120.1 |
| 815 | heterogeneous nuclear ribonucleoprotein R | | | XP_001111802.1 |
| 816 | heterogeneous nuclear ribonucleoprotein U isoform a | | | XP_001102264.1 |
| 817 | heterogeneous nuclear ribonucleoprotein U | | | XP_001116183.1 |
| 818 | heterogeneous nuclear ribonucleoproteins methyltransferase-like 2 | | | XP_001113609.1 |
| 819 | hexosaminidase (glycosyl hydrolase family 20, catalytic domain) containing | | | XP_001113324.1 |
| 820 | HGF activator preproprotein | | | XP_001114822.1 |
| 821 | High mobility group protein B1 (High mobility group protein 1) | | | XP_001102554.1 |
| 822 | high-mobility group box 2 | | | XP_001085665.1 |
| 823 | histamine N-methyltransferase | | | XP_001094746.1 |
| 824 | histidine ammonia-lyase | | | XP_001107685.1 |
| 825 | Histone binding protein RBBP7-like protein | | | tr|A6ML29|A6ML29_CALJA |
| 826 | histone deacetylase 1 | | | XP_001096929.1 |
| 827 | histone family H2A, member Y isoform 3 | | | XP_001110552.1 |
| 828 | histone family H2A, member Z isoform 2 | | | XP_001108128.1 |
| 829 | histone family H2B, member E | | | XP_001096102.1 |
| 830 | histone family H2B, member F | | | XP_001090500.1 |
| 831 | Histone H1.2 (H1d) | | | XP_001087823.1 |
| 832 | Histone H1t | | | sp|P40286|H1T_MACMU |
| 833 | Histone H2A type 1 isoform 1 | | | XP_001097115.1 |
| 834 | Histone H2A type 1-D (H2A.3) isoform 1 | | | XP_001086885.1 |
| 835 | Histone H2B 291B | | | XP_001104697.1 |
| 836 | histone H3, family 3B | | | XP_001104869.1 |
| 837 | HLA class II histocompatibility antigen, DR alpha chain precursor (MHC class II antigen DRA) | | | XP_001119594.1 |
| 838 | Homeobox protein Nkx-2.4 (Homeobox protein NKX2.4) (Homeobox protein NK-2 homolog D) | | | XP_001093634.1 |
| 839 | HP1-BP74 isoform 3 | | | XP_001098287.1 |
| 840 | hyaluronan binding protein 2 | | | XP_001090138.1 |
| 841 | hydroxyacylglutathione hydrolase-like isoform 2 | | | XP_001087099.1 |
| 842 | hypermethylated in cancer 2 | | | XP_001086914.1 |
| 843 | hypothetical protein isoform 2 | | | XP_001090547.1 |
| 844 | hypothetical protein isoform 3 | | | XP_001091507.1 |
| 845 | hypothetical protein isoform 4 | | | XP_001087439.1 |
| 846 | hypothetical protein isoform 8 | | | XP_001113568.1 |
| 847 | hypothetical protein LOC23076 | | | XP_001104749.1 |
| 848 | hypothetical protein LOC23306 | | | XP_001099369.1 |
| 849 | hypothetical protein LOC23331, partial | | | XP_001108189.1 |
| 850 | hypothetical protein LOC51098 isoform 2 | | | XP_001085430.1 |
| 851 | hypothetical protein LOC55102 | | | XP_001101467.1 |
| 852 | hypothetical protein LOC60686 isoform 1 | | | XP_001104382.1 |
| 853 | hypothetical protein LOC695578 [Macaca mulatta] | | | NP_001103153.1 |
| 854 | hypothetical protein LOC700391 [Macaca mulatta] | | | NP_001041710.1 |
| 855 | hypothetical protein LOC711832 [Macaca mulatta] | | | NP_001041711.1 |
| 856 | hypothetical protein, partial | | | XP_001119223.1 |
| 857 | hypothetical protein | | | XP_001107765.1 |
| 858 | Hypoxanthine-guanine phosphoribosyltransferase (HGPRT) (HGPRTase) | | | XP_001097691.1 |
| 859 | hypoxia-inducible factor prolyl 4-hydroxylase isoform a | | | XP_001096609.1 |
| 860 | Ig heavy constant gamma 1 | | | XP_001100439.1 |
| 861 | Ig gamma-1 chain C region | | | XP_001100082.1 |
| 862 | Ig heavy chain V-II region SESS precursor, partial | | | XP_001117187.1 |
| 863 | Ig kappa chain V-II region RPMI 6410 precursor, partial | | | XP_001119706.1 |
| 864 | Ig lambda-like polypeptide 1 precursor (Immunoglobulin-related protein 14.1) (CD179b antigen) | | | XP_001097034.1 |
| 865 | Ig omega chain precursor (VpreB2 protein) | | | XP_001092612.1 |
| 866 | Ig superfamily, member 4A isoform b | | | XP_001117142.1 |
| 867 | Ig superfamily, member 4D isoform 6 | | | XP_001089728.1 |
| 868 | importin 8 | | | XP_001082473.1 |
| 869 | Indoleamine 2,3-dioxygenase (IDO) (Indoleamine-pyrrole 2,3-dioxygenase), partial | | | XP_001119699.1 |
| 870 | Inhibitor of growth family, member 1-like | | | tr|Q3YAR5|Q3YAR5_MACMU |
| 871 | inosine monophosphate dehydrogenase 2 isoform 6 | | | XP_001110855.1 |
| 872 | insulin-like growth factor 2 mRNA binding protein 2 isoform a isoform 5 | | | XP_001095336.1 |
| 873 | Integrase | | | tr|A5YN64|A5YN64_SIVCZ |
| 874 | integrin alpha 2 | | | XP_001095246.1 |
| 875 | integrin alpha 2b | | | XP_001114526.1 |
| 876 | integrin alpha L | | | XP_001100800.1 |
| 877 | integrin alpha-V | | | XP_001104012.1 |
| 878 | Integrin beta 2-like protein | | | tr|A6MJV5|A6MJV5_CALJA |
| 879 | integrin beta chain, beta 3 precursor isoform 2 | | | XP_001116013.1 |
| 880 | integrin, beta 8 isoform 2 | | | XP_001103130.1 |
| 881 | Integrin-linked kinase | | | tr|Q3YAQ5|Q3YAQ5_MACMU |
| 882 | integrin-linked kinase isoform 1 | | | XP_001108844.1 |
| 883 | integrin-linked kinase isoform 2 | | | XP_001108986.1 |
| 884 | integrin-linked kinase-associated protein phosphatase 2C isoform 1 | | | XP_001094705.1 |
| 885 | Integrin-linked protein kinase-like protein | | | tr|A6ML88|A6ML88_CALJA |
| 886 | inter-alpha (globulin) inhibitor H1 | | | XP_001084712.1 |
| 887 | inter-alpha (globulin) inhibitor H3 | | | XP_001085463.1 |
| 888 | inter-alpha globulin inhibitor H2 polypeptide | | | XP_001107718.1 |
| 889 | Interferon alpha 1 | | | tr|B6CK06|B6CK06_CERTO |
| 890 | Interferon alpha 2 | | | tr|B6CK12|B6CK12_CERTO |
| 891 | Interferon beta 1 | | | tr|B6CK13|B6CK13_CERTO |
| 892 | Interferon gamma | | | sp|P42162|IFNG_CERTO |
| 893 | interferon induced transmembrane protein 1 (9-27) | | | XP_001085444.1 |
| 894 | interferon induced transmembrane protein 3 (1-8U) isoform 1 | | | XP_001112533.1 |
| 895 | Interferon regulatory factor 2 | | | tr|B6CJX7|B6CJX7_CERTO |
| 896 | Interferon regulatory factor 3 | | | tr|B6CJX5|B6CJX5_CERTO |
| 897 | interferon regulatory factor 4 | | | XP_001118967.1 |
| 898 | Interferon regulatory factor 7 | | | tr|B6CJX3|B6CJX3_CERTO |
| 899 | interferon, alpha-inducible protein (clone IFI-15K) | | | XP_001088541.1 |
| 900 | interferon, gamma-inducible protein 16 isoform 2 | | | XP_001117168.1 |
| 901 | interferon, omega 1 | | | XP_001108113.1 |
| 902 | interferon-induced protein with tetratricopeptide repeats 2 | | | XP_001086302.1 |
| 903 | interferon-induced protein with tetratricopeptide repeats 3 | | | XP_001086192.1 |
| 904 | interleukin enhancer binding factor 3 isoform a | | | XP_001102411.1 |
| 905 | Interleukin-1 alpha | | | sp|P46647|IL1A_CERTO |
| 906 | Interleukin-1 beta | | | sp|P46648|IL1B_CERTO |
| 907 | interleukin-1 receptor, type I | | | XP_001107510.1 |
| 908 | Interleukin-1 receptor-associated kinase 1 | | | tr|B6CJY2|B6CJY2_CERTO |
| 909 | Interleukin-1 receptor-associated kinase 4 | | | tr|B6CJY0|B6CJY0_CERTO |
| 910 | Interleukin-10 | | | sp|P46651|IL10_CERTO |
| 911 | Interleukin-12 | | | sp|P46661|IL12A_CERTO |
| 912 | interleukin-12A | | | NP_001038199.1 |
| 913 | Interleukin-13 | | | tr|Q0ZB84|Q0ZB84_CERTO |
| 914 | Interleukin-2 | | | sp|P46649|IL2_CERTO |
| 915 | interleukin-28 receptor, alpha isoform 1 isoform 2 | | | XP_001105189.1 |
| 916 | Interleukin-6 | | | sp|P46650|IL6_CERTO |
| 917 | Involucrin, partial | | | XP_001119791.1 |
| 918 | IQ motif containing G | | | XP_001103600.1 |
| 919 | iron-responsive element binding protein 2 | | | XP_001107837.1 |
| 920 | Isocitrate dehydrogenase [NAD] subunit gamma, mitochondrial precursor (Isocitric dehydrogenase) | | | XP_001087052.1 |
| 921 | isocitrate dehydrogenase 1 (NADP+), soluble isoform 1 | | | XP_001107627.1 |
| 922 | isocitrate dehydrogenase 2 (NADP+), mitochondrial | | | XP_001095686.1 |
| 923 | isoleucine-tRNA synthetase | | | XP_001107299.1 |
| 924 | Jade1 protein long isoform isoform 6 | | | XP_001083740.1 |
| 925 | Jak and microtubule interacting protein 2 | | | XP_001102707.1 |
| 926 | jumonji domain containing 1C isoform 8 | | | XP_001091903.1 |
| 927 | junction plakoglobin | | | XP_001107394.1 |
| 928 | kallikrein 14, partial | | | XP_001119583.1 |
| 929 | kallikrein 15 isoform 6 | | | XP_001116199.1 |
| 930 | karyopherin alpha 4 | | | XP_001097222.1 |
| 931 | karyopherin alpha 6 isoform 4 | | | XP_001102113.1 |
| 932 | karyopherin beta 1 | | | XP_001082833.1 |
| 933 | katanin p60 subunit A 1 | | | XP_001085922.1 |
| 934 | Kazal-type serine protease inhibitor domain 1 | | | XP_001109857.1 |
| 935 | kelch domain containing 4 | | | XP_001086874.1 |
| 936 | kelch repeat and BTB (POZ) domain containing 10 | | | XP_001104263.1 |
| 937 | keratin 1 isoform 7 | | | XP_001098292.1 |
| 938 | keratin 10 isoform 3 | | | XP_001100664.1 |
| 939 | keratin 15 isoform 2 | | | XP_001106818.1 |
| 940 | keratin 19 | | | XP_001107201.1 |
| 941 | keratin 1B, partial | | | XP_001112449.1 |
| 942 | keratin 23 | | | XP_001101954.1 |
| 943 | keratin 24, partial | | | XP_001110974.1 |
| 944 | keratin 2a | | | XP_001098486.1 |
| 945 | keratin 4 isoform 2 | | | XP_001099304.1 |
| 946 | keratin, hair, basic, 3 | | | XP_001093322.1 |
| 947 | Keratin, type II cytoskeletal 6A (Cytokeratin-6A) (CK 6A) (K6a keratin) | | | XP_001112347.1 |
| 948 | KH domain-containing, RNA-binding, signal transduction-associated protein 2 | | | XP_001111106.1 |
| 949 | KIAA0020 isoform 1 | | | XP_001084706.1 |
| 950 | kinesin family member 11 | | | XP_001087644.1 |
| 951 | kinesin family member 14 | | | XP_001109736.1 |
| 952 | kinesin family member 3C isoform 8 | | | XP_001085440.1 |
| 953 | kinesin family member 4 | | | XP_001084213.1 |
| 954 | kinesin family member C3 | | | XP_001100393.1 |
| 955 | kinesin-like motor protein C20orf23, partial | | | XP_001101003.1 |
| 956 | kininogen 1 isoform 4 | | | XP_001102055.1 |
| 957 | klotho beta like | | | XP_001091413.1 |
| 958 | Kruppel-like factor 7 (ubiquitous) isoform 2 | | | XP_001107009.1 |
| 959 | Ku autoantigen | | | tr|Q6UIL6|Q6UIL6_PANTR |
| 960 | lactate dehydrogenase A isoform 6 | | | XP_001084747.1 |
| 961 | lactate dehydrogenase A | | | XP_001086967.1 |
| 962 | lactate dehydrogenase B | | | XP_001117178.1 |
| 963 | Ladybird homeobox corepressor 1 | | | XP_001091453.1 |
| 964 | laminin alpha 2 subunit precursor | | | XP_001105600.1 |
| 965 | laminin alpha 3 subunit isoform 1 isoform 3 | | | XP_001095558.1 |
| 966 | laminin receptor 1 (ribosomal protein SA) | | | XP_001116516.1 |
| 967 | laminin, beta 1 | | | XP_001090393.1 |
| 968 | latent transforming growth factor beta binding protein 3 | | | XP_001118134.1 |
| 969 | LEA_4 domain containing protein RGD1359600 | | | XP_001111295.1 |
| 970 | lectin, galactoside-binding, soluble, 1 (galectin 1) isoform 2 | | | XP_001083427.1 |
| 971 | lectin, galactoside-binding, soluble, 14 isoform 2 | | | XP_001088117.1 |
| 972 | lectin, galactoside-binding, soluble, 7 (galectin 7) | | | XP_001083444.1 |
| 973 | leucine aminopeptidase 3 isoform 1 | | | XP_001102528.1 |
| 974 | leucine aminopeptidase isoform 2 | | | XP_001102787.1 |
| 975 | leucine rich repeat (in FLII) interacting protein 1 | | | XP_001092734.1 |
| 976 | leucine rich repeat containing 14 isoform 4 | | | XP_001094683.1 |
| 977 | leucine rich repeat containing 19 | | | XP_001105486.1 |
| 978 | leucine rich repeat containing 7 isoform 2 | | | XP_001097185.1 |
| 979 | leucine rich repeat containing 8 family, member E | | | XP_001093244.1 |
| 980 | leucine zipper protein 1 | | | XP_001111566.1 |
| 981 | Leucine-rich acidic nuclear phosphoprotein 32 family, member E | | | tr|Q3YAN1|Q3YAN1_MACMU |
| 982 | leucine-rich repeat kinase 1 | | | XP_001093275.1 |
| 983 | leucyl-tRNA synthetase | | | XP_001095167.1 |
| 984 | leupaxin | | | XP_001093443.1 |
| 985 | LIM and senescent cell antigen-like domains 1 | | | XP_001082828.1 |
| 986 | LIM and senescent cell antigen-like domains 2 isoform 9 | | | XP_001088922.1 |
| 987 | lipopolysaccharide-binding protein | | | XP_001094451.1 |
| 988 | lipoxygenase homology domains 1, partial | | | XP_001119151.1 |
| 989 | L-lactate dehydrogenase B chain-like protein | | | tr|A6MK87|A6MK87_CALJA |
| 990 | LON peptidase N-terminal domain and ring finger 2 | | | XP_001104504.1 |
| 991 | loss of heterozygosity, 11, chromosomal region 2, gene A homolog | | | XP_001099634.1 |
| 992 | low density lipoprotein receptor adaptor protein 1 isoform 2 | | | XP_001107620.1 |
| 993 | low density lipoprotein-related protein 1, partial | | | XP_001099678.1 |
| 994 | L-plastin isoform 5 | | | XP_001098697.1 |
| 995 | LPLUNC1 protein | | | XP_001105754.1 |
| 996 | lumican isoform 1 | | | XP_001102667.1 |
| 997 | lumican isoform 2 | | | XP_001102753.1 |
| 998 | Lupus La-like protein | | | tr|A6MKG9|A6MKG9_CALJA |
| 999 | Lysosomal-associated multitransmembrane protein (Retinoic acid-inducible E3 protein) (HA1520) | | | XP_001092380.1 |
| 1000 | Lysozyme C | | | sp|P61630|LYSC_CERTO |
| 1001 | lysyl oxidase-like 4 | | | XP_001096621.1 |
| 1002 | lysyl-tRNA synthetase | | | XP_001104312.1 |
| 1003 | Lysyl-tRNA synthetase-like protein | | | tr|A6MK47|A6MK47_CALJA |
| 1004 | mab-21-like protein 2 | | | XP_001082398.1 |
| 1005 | Macrophage capping protein-like protein | | | tr|A6MKP8|A6MKP8_CALJA |
| 1006 | mago-nashi homolog isoform 1 | | | XP_001115401.1 |
| 1007 | Major capsid protein | | | tr|Q805V9|Q805V9_CHV16 |
| 1008 | Malate dehydrogenase | | | tr|A6ML54|A6ML54_CALJA |
| 1009 | mannan-binding lectin serine protease 2 isoform 1 precursor | | | XP_001118815.1 |
| 1010 | mannosyl-oligosaccharide glucosidase | | | XP_001109499.1 |
| 1011 | MAP/microtubule affinity-regulating kinase 2 isoform 9 | | | XP_001115611.1 |
| 1012 | MARVEL domain containing 2 isoform beta isoform 1 | | | XP_001094293.1 |
| 1013 | maternal embryonic leucine zipper kinase | | | XP_001115076.1 |
| 1014 | matrix metalloproteinase 3 isoform 2 | | | XP_001098492.1 |
| 1015 | MAWD binding protein | | | XP_001086075.1 |
| 1016 | mediator of RNA polymerase II transcription, subunit 13 homolog isoform 2 | | | XP_001110128.1 |
| 1017 | mediator of RNA polymerase II transcription, subunit 18 homolog | | | XP_001113062.1 |
| 1018 | mediator of RNA polymerase II transcription, subunit 8 homolog (S. cerevisiae) isoform 5 | | | XP_001094112.1 |
| 1019 | meiosis-specific nuclear structural protein 1 isoform 2 | | | XP_001091177.1 |
| 1020 | meiotic recombination protein SPO11 isoform a isoform 2 | | | XP_001088572.1 |
| 1021 | melanoma antigen family A, 10 | | | XP_001099898.1 |
| 1022 | melanoma antigen family D, 2 isoform 2 | | | XP_001091068.1 |
| 1023 | melanoma-associated chondroitin sulfate proteoglycan 4 | | | XP_001106160.1 |
| 1024 | membrane associated guanylate kinase, WW and PDZ domain containing 1 isoform c isoform 4 | | | XP_001091622.1 |
| 1025 | membrane component chromosome 11 surface marker 1 isoform 1 | | | XP_001115730.1 |
| 1026 | membrane-spanning 4-domains, subfamily A, member 1 | | | XP_001086135.1 |
| 1027 | metalloprotease 1 | | | XP_001116336.1 |
| 1028 | metastasis associated 1 family, member 3 isoform 4 | | | XP_001110956.1 |
| 1029 | metastasis-associated protein 2 | | | XP_001116490.1 |
| 1030 | methionine adenosyltransferase I, alpha | | | XP_001087977.1 |
| 1031 | methionine-tRNA synthetase 2 precursor | | | XP_001086799.1 |
| 1032 | methionine-tRNA synthetase isoform 4 | | | XP_001116063.1 |
| 1033 | Methionyl-tRNA synthetase-like protein | | | tr|A6MK77|A6MK77_CALJA |
| 1034 | methyl-CpG binding domain protein 3 | | | XP_001095979.1 |
| 1035 | methylcrotonoyl-Coenzyme A carboxylase 1 (alpha) | | | XP_001096717.1 |
| 1036 | methylenetetrahydrofolate dehydrogenase 1 isoform 2 | | | XP_001101889.1 |
| 1037 | Methylosome subunit pICln (Chloride conductance regulatory protein ICln) (I(Cln)) | | | XP_001088333.1 |
| 1038 | methyltransferase 5 domain containing 1 | | | XP_001088477.1 |
| 1039 | MHC (Fragment) | | | tr|Q30740|Q30740_MACNE |
| 1040 | MHC class 1 | | | NP_001073139.2 |
| 1041 | MHC class I antigen | | | tr|A5YWB6|A5YWB6_MACFA |
| 1042 | MHC class I antigen A-74 alpha chain precursor | | | XP_001119760.1 |
| 1043 | MHC class I antigen B-37 chain precursor | | | XP_001115190.1 |
| 1044 | MHC class I antigen B-38 alpha chain precursor | | | XP_001117018.1 |
| 1045 | MHC class I antigen Cw-8 alpha chain precursor | | | XP_001115753.1 |
| 1046 | MHC class I antigen DRB1-4 beta chain precursor isoform 6 | | | XP_001089877.1 |
| 1047 | MHC class I, E precursor | | | XP_001119007.1 |
| 1048 | MHC class II | | | tr|Q7YQ20|Q7YQ20_MACMU |
| 1049 | MHC class II antigen | | | tr|Q9TP91|Q9TP91_9PRIM |
| 1050 | MHC class II antigen DO beta chain precursor | | | XP_001115393.1 |
| 1051 | MHC class l heavy chain antigen | | | tr|Q9MXT2|Q9MXT2_MACMU |
| 1052 | MHC class ll antigen - DR alpha chain precursor | | | XP_001119594.1 |
| 1053 | MHC class ll antigen, DP(W2) beta chain precursor | | | XP_001115895.1 |
| 1054 | MHC class ll antigen, DQ(1) beta chain precursor | | | XP_001116137.1 |
| 1055 | MHC class ll antigen, DQ(2) alpha chain precursor | | | XP_001116145.1 |
| 1056 | MHC DQ-alpha 1 protein (Fragment) | | | tr|Q30994|Q30994_PANTR |
| 1057 | microcephalin | | | XP_001097449.1 |
| 1058 | microtubule associated monoxygenase, calponin and LIM domain containing 3 | | | XP_001103660.1 |
| 1059 | microtubule associated serine/threonine kinase 2 isoform 9 | | | XP_001105315.1 |
| 1060 | microtubule-associated protein 1B | | | XP_001097124.1 |
| 1061 | microtubule-associated protein, RP/EB family, member 2 isoform 8 | | | XP_001103596.1 |
| 1062 | microtubule-associated proteins 1A/1B light chain 3 | | | XP_001100898.1 |
| 1063 | Microtubule-associated serine/threonine-protein kinase 2 | | | XP_001088404.1 |
| 1064 | mindbomb homolog 1 | | | XP_001092086.1 |
| 1065 | minichromosome maintenance protein 10 isoform 2 | | | XP_001085751.1 |
| 1066 | minichromosome maintenance protein 2 | | | XP_001099580.1 |
| 1067 | minichromosome maintenance protein 3 isoform 3 | | | XP_001106966.1 |
| 1068 | minichromosome maintenance protein 6 isoform 1 | | | XP_001096068.1 |
| 1069 | minor histocompatibility antigen HA-1 | | | XP_001117237.1 |
| 1070 | Mitochondrial import inner membrane translocase subunit Tim9 B (TIMM10B) isoform 2 | | | XP_001109886.1 |
| 1071 | mitochondrial malate dehydrogenase precursor | | | XP_001114888.1 |
| 1072 | mitochondrial ribosomal protein L27 | | | XP_001097308.1 |
| 1073 | mitochondrial ribosomal protein L52 isoform c isoform 2 | | | XP_001099958.1 |
| 1074 | mitochondrial ribosomal protein S22 isoform 6 | | | XP_001113756.1 |
| 1075 | mitogen-activated protein kinase 1 | | | XP_001089600.1 |
| 1076 | Mitogen-activated protein kinase 8 (Stress-activated protein kinase JNK1) isoform 1 | | | XP_001108716.1 |
| 1077 | mitogen-activated protein kinase kinase 1 | | | XP_001110225.1 |
| 1078 | mitogen-activated protein kinase kinase 3 isoform B isoform 3 | | | XP_001104193.1 |
| 1079 | mitogen-activated protein kinase kinase kinase 11 | | | XP_001113486.1 |
| 1080 | mitogen-activated protein kinase kinase kinase 7 interacting protein 2 | | | XP_001096046.1 |
| 1081 | mitogen-activated protein kinase kinase kinase kinase 1 | | | XP_001082963.1 |
| 1082 | Mitotic checkpoint protein BUB3-like protein | | | tr|A6ML19|A6ML19_CALJA |
| 1083 | Mitotic spindle assembly checkpoint protein MAD2A (MAD2-like 1) (HsMAD2) | | | XP_001097054.1 |
| 1084 | Mob4B protein | | | XP_001107567.1 |
| 1085 | MOB-LAK, partial | | | XP_001108825.1 |
| 1086 | moesin | | | XP_001100546.1 |
| 1087 | molecule interacting with Rab13 | | | XP_001090486.1 |
| 1088 | mRNA decapping enzyme | | | XP_001109581.1 |
| 1089 | MTERF domain containing 2 | | | XP_001109015.1 |
| 1090 | mucin 5, subtype B, tracheobronchial, partial | | | XP_001116863.1 |
| 1091 | mucolipin 2 | | | XP_001107980.1 |
| 1092 | mucosa associated lymphoid tissue lymphoma translocation protein 1 | | | XP_001086151.1 |
| 1093 | multimerin 1 isoform 2 | | | XP_001101702.1 |
| 1094 | muscle-type acylphosphatase 2 | | | XP_001114543.1 |
| 1095 | Myeloid cell nuclear differentiation antigen | | | sp|Q8SPH9|MNDA_MACFA |
| 1096 | myeloid cell nuclear differentiation antigen | | | XP_001117129.1 |
| 1097 | Myeloid differentiation primary response protein 88 | | | tr|B6CJX2|B6CJX2_CERTO |
| 1098 | myeloid translocation gene-related protein 2 isoform MTG16a | | | XP_001090215.1 |
| 1099 | myosin binding protein C, slow type isoform 1 | | | XP_001091952.1 |
| 1100 | Myosin heavy chain, cardiac muscle alpha isoform (MyHC-alpha) | | | XP_001095819.1 |
| 1101 | Myosin heavy chain, fast skeletal muscle, embryonic isoform 3 | | | XP_001114028.1 |
| 1102 | myosin IC | | | XP_001117297.1 |
| 1103 | myosin IF | | | XP_001100995.1 |
| 1104 | myosin IXA | | | XP_001089813.1 |
| 1105 | myosin IXB | | | XP_001114282.1 |
| 1106 | Myosin regulatory light chain 2 , nonsarcomeric (Myosin RLC) isoform 2 | | | XP_001084519.1 |
| 1107 | myosin VA (heavy polypeptide 12, myoxin) | | | XP_001084476.1 |
| 1108 | myosin VB isoform 3 | | | XP_001090668.1 |
| 1109 | myosin, heavy polypeptide 14 | | | XP_001113969.1 |
| 1110 | myosin, heavy polypeptide 9, non-muscle, partial | | | XP_001116728.1 |
| 1111 | myosin, heavy polypeptide 9, non-muscle | | | XP_001083662.1 |
| 1112 | myosin, light polypeptide 6, alkali, smooth muscle and non-muscle isoform 7 | | | XP_001114459.1 |
| 1113 | myosin, light polypeptide kinase isoform 7 | | | XP_001113525.1 |
| 1114 | Myosin-1 (Myosin heavy chain D) (MHC D) | | | XP_001095126.1 |
| 1115 | Myosin-11 (Myosin heavy chain, smooth muscle isoform) (SMMHC) | | | XP_001118953.1 |
| 1116 | Myosin-3 (Myosin heavy chain A) (MHC A) isoform 5 | | | XP_001103275.1 |
| 1117 | Myosin-9 (Myosin heavy chain, nonmuscle IIa) (Nonmuscle myosin heavy chain IIa), partial | | | XP_001116722.1 |
| 1118 | myosin-reactive immunoglobulin light chain variable region | | | XP_001089800.1 |
| 1119 | myostatin precursor | | | NP_001073588.1 |
| 1120 | myotonic dystrophy protein kinase isoform 2 | | | XP_001111330.1 |
| 1121 | myotrophin isoform 1 | | | XP_001106584.1 |
| 1122 | myotubularin-related protein 3 isoform c isoform 5 | | | XP_001107561.1 |
| 1123 | myxovirus resistance protein 1 | | | NP_001073161.1 |
| 1124 | Na+/K+ -ATPase alpha 1 subunit isoform 3 | | | XP_001112674.1 |
| 1125 | Na+/K+ -ATPase alpha 2 subunit proprotein | | | XP_001115318.1 |
| 1126 | N-Acetylglucosamine kinase isoform 3 | | | XP_001102037.1 |
| 1127 | N-acetylneuraminate pyruvate lyase isoform 3 | | | XP_001114494.1 |
| 1128 | N-acetyltransferase 1 isoform 2 | | | XP_001098534.1 |
| 1129 | NACHT, leucine rich repeat and PYD containing 4 isoform 3 | | | XP_001090438.1 |
| 1130 | N-acylaminoacyl-peptide hydrolase | | | XP_001108167.1 |
| 1131 | NADH dehydrogenase (ubiquinone) 1 alpha subcomplex, 10, 42kDa precursor isoform 2 | | | XP_001088031.1 |
| 1132 | NADH-ubiquinone oxidoreductase 75 kDa subunit, mitochondrial | | | sp|Q0MQG2|NDUS1_PANTR |
| 1133 | nardilysin (N-arginine dibasic convertase) | | | XP_001105075.1 |
| 1134 | Nascent polypeptide-associated complex alpha subunit, muscle-specific form | | | XP_001115411.1 |
| 1135 | nasopharyngeal epithelium specific protein 1 | | | XP_001115183.1 |
| 1136 | NCK-associated protein 1 isoform 2 | | | XP_001099879.1 |
| 1137 | N-deacetylase/N-sulfotransferase (heparan glucosaminyl) 3 isoform 2 | | | XP_001098030.1 |
| 1138 | nebulin | | | XP_001084585.1 |
| 1139 | Nef protein | | | tr|A0EUQ8|A0EUQ8_9HIV1 |
| 1140 | nei endonuclease VIII-like 3 | | | XP_001090459.1 |
| 1141 | nemo like kinase | | | XP_001105594.1 |
| 1142 | neogenin homolog 1 | | | XP_001093533.1 |
| 1143 | nesca protein isoform 1 | | | XP_001115980.1 |
| 1144 | nesprin 1 | | | XP_001100631.1 |
| 1145 | N-ethylmaleimide-sensitive factor attachment protein, alpha isoform 1 | | | XP_001113044.1 |
| 1146 | netrin receptor Unc5h4 | | | XP_001088208.1 |
| 1147 | neural cell adhesion molecule 1 isoform 7 | | | XP_001083697.1 |
| 1148 | neural cell adhesion molecule 1 isoform 9 | | | XP_001083924.1 |
| 1149 | neuregulin 1 isoform HRG-beta1 isoform 4 | | | XP_001086184.1 |
| 1150 | neurexin 1 isoform beta | | | XP_001114208.1 |
| 1151 | neurofibromin 2 isoform 1 isoform 2 | | | XP_001106489.1 |
| 1152 | neurofilament light polypeptide | | | tr|Q4G3Y3|Q4G3Y3_MACMU |
| 1153 | neuroligin 3 | | | XP_001086823.1 |
| 1154 | neurolysin isoform 3 | | | XP_001087239.1 |
| 1155 | neuropilin 1 isoform 12 | | | XP_001087258.1 |
| 1156 | neuropilin 1 isoform 13 | | | XP_001087374.1 |
| 1157 | neuropilin 2 isoform 6 precursor | | | XP_001104807.1 |
| 1158 | niban protein isoform 2 isoform 2 | | | XP_001113897.1 |
| 1159 | nidogen (enactin) isoform 2 | | | XP_001100451.1 |
| 1160 | NIK and IKK(beta) binding protein, partial | | | XP_001100264.1 |
| 1161 | NIMA (never in mitosis gene a)-related kinase 7 | | | XP_001110532.1 |
| 1162 | NIMA-related kinase 3 isoform 3 | | | XP_001107019.1 |
| 1163 | NIMA-related kinase 8 | | | XP_001106986.1 |
| 1164 | nin one binding protein | | | XP_001101319.1 |
| 1165 | NMDA receptor regulated 1 | | | XP_001087829.1 |
| 1166 | NmrA-like family domain containing 1 isoform 2 | | | XP_001096324.1 |
| 1167 | N-myristoyltransferase 1 isoform 2 | | | XP_001115181.1 |
| 1168 | nodal modulator 2 isoform 2 | | | XP_001109510.1 |
| 1169 | non-imprinted in Prader-Willi/Angelman syndrome 1 | | | XP_001106326.1 |
| 1170 | non-metastatic cells 1, protein (NM23A) expressed in isoform a | | | XP_001096144.1 |
| 1171 | nonmuscle myosin heavy chain isoform 6 | | | XP_001095550.1 |
| 1172 | non-POU domain containing, octamer-binding isoform 1 | | | XP_001090372.1 |
| 1173 | NSE1 | | | XP_001090187.1 |
| 1174 | nuclear antigen Sp100 isoform 9 | | | XP_001112681.1 |
| 1175 | nuclear autoantigenic sperm protein (histone-binding) isoform 2 | | | XP_001103320.1 |
| 1176 | nuclear DNA-binding protein | | | XP_001094383.1 |
| 1177 | nuclear mitotic apparatus protein 1 isoform 13 | | | XP_001114209.1 |
| 1178 | nuclear protein UKp68 isoform 1 isoform 13 | | | XP_001085857.1 |
| 1179 | nuclear receptor binding protein isoform 5 | | | XP_001096971.1 |
| 1180 | nuclear receptor binding SET domain protein 1 isoform b, partial | | | XP_001094467.1 |
| 1181 | nuclear receptor coactivator 4 isoform 7 | | | XP_001106334.1 |
| 1182 | Nuclear receptor subfamily 0 group B member 1 | | | sp|Q9BG94|NR0B1_CALJA |
| 1183 | nuclear receptor subfamily 2, group C, member 2 | | | XP_001091445.1 |
| 1184 | nuclear receptor subfamily 3, group C, member 2 isoform 2 | | | XP_001099653.1 |
| 1185 | nuclear RNA export factor 1 isoform 5 | | | XP_001115982.1 |
| 1186 | nuclear transport factor 2 | | | XP_001108784.1 |
| 1187 | nucleobindin 1, partial | | | XP_001118735.1 |
| 1188 | nucleolar protein 11 | | | XP_001090482.1 |
| 1189 | nucleolar protein 5A | | | XP_001110561.1 |
| 1190 | nucleolar protein NOP5/NOP58 | | | XP_001101291.1 |
| 1191 | nucleolin | | | XP_001116949.1 |
| 1192 | nucleophosmin 1 isoform 1 isoform 1 | | | XP_001099639.1 |
| 1193 | nucleophosmin 1 isoform 2 | | | XP_001095288.1 |
| 1194 | nucleosome assembly protein 1-like 1 isoform 8 | | | XP_001117640.1 |
| 1195 | nucleosome assembly protein 1-like 4 isoform 4 | | | XP_001095198.1 |
| 1196 | nucleotide-binding oligomerization domains 27 | | | XP_001095341.1 |
| 1197 | nudix (nucleoside diphosphate linked moiety X)-type motif 17 | | | XP_001089939.1 |
| 1198 | nudix-type motif 11 | | | XP_001084607.1 |
| 1199 | odd Oz/ten-m homolog 3 isoform 4 | | | XP_001091761.1 |
| 1200 | olfactomedin-like 2A | | | XP_001082486.1 |
| 1201 | Olfactory receptor | | | tr|Q6SN75|Q6SN75_CERAG |
| 1202 | Olfactory receptor 11H4 (Olfactory receptor OR14-36) | | | XP_001087477.1 |
| 1203 | Olfactory receptor 2L2 (HTPCRH07) | | | XP_001095076.1 |
| 1204 | Olfactory receptor 2W3 (Olfactory receptor OR1-49) isoform 2 | | | XP_001086118.1 |
| 1205 | Olfactory receptor 8H1 | | | XP_001103843.1 |
| 1206 | olfactory receptor 958 | | | XP_001105305.1 |
| 1207 | olfactory receptor Olr383 | | | XP_001090489.1 |
| 1208 | olfactory receptor Olr95 | | | XP_001106533.1 |
| 1209 | olfactory receptor, family 2, subfamily T, member 8 | | | XP_001095606.1 |
| 1210 | olfactory receptor, family 4, subfamily K, member 15 | | | XP_001086778.1 |
| 1211 | oligophrenin 1 | | | XP_001091450.1 |
| 1212 | oncoprotein-induced transcript 3 | | | XP_001104470.1 |
| 1213 | optineurin | | | NP_001028069.1 |
| 1214 | ORF15 | | | tr|Q993J5|Q993J5_9GAMA |
| 1215 | ORF16 | | | tr|Q993J4|Q993J4_9GAMA |
| 1216 | ORF58 | | | tr|Q8BEN8|Q8BEN8_9GAMA |
| 1217 | ornithine carbamoyltransferase precursor, partial | | | XP_001105675.1 |
| 1218 | O-sialoglycoprotein endopeptidase | | | XP_001088971.1 |
| 1219 | osteoclast inhibitory lectin isoform 2 isoform 4 | | | XP_001114650.1 |
| 1220 | osteomodulin isoform 3 | | | XP_001103592.1 |
| 1221 | cyclophilin A | | | NP_001027981.1 |
| 1222 | OTU domain, ubiquitin aldehyde binding 1 | | | XP_001115486.1 |
| 1223 | outer dense fiber of sperm tails 2 isoform 1 isoform 21 | | | XP_001111784.1 |
| 1224 | ovochymase 1 | | | XP_001101446.1 |
| 1225 | p21 (CDKN1A)-activated kinase 2 isoform 2 | | | XP_001099936.1 |
| 1226 | p30 DBC protein | | | XP_001106923.1 |
| 1227 | p53-induced protein | | | XP_001105448.1 |
| 1228 | pancreatic carboxypeptidase A1 isoform 3 | | | XP_001096893.1 |
| 1229 | pannexin 3 | | | XP_001109098.1 |
| 1230 | parvin, beta isoform b, partial | | | XP_001106339.1 |
| 1231 | PDCD8 (Fragment) | | | tr|Q005U5|Q005U5_FELCA |
| 1232 | PDZ domain containing 10 | | | XP_001095042.1 |
| 1233 | pentatricopeptide repeat domain 1 | | | XP_001111767.1 |
| 1234 | pentraxin-related gene, rapidly induced by IL-1 beta | | | XP_001103515.1 |
| 1235 | peptidase (prosome, macropain) 26S subunit, ATPase 1 | | | XP_001103312.1 |
| 1236 | Peptidyl-prolyl cis-trans isomerase (Fragment) | | | tr|A6MKR8|A6MKR8_CALJA |
| 1237 | peptidylprolyl isomerase A isoform 1 | | | XP_001104554.1 |
| 1238 | peptidylprolyl isomerase-like 2 isoform a | | | XP_001088432.1 |
| 1239 | perforin 1 isoform 2 | | | XP_001107967.1 |
| 1240 | periostin, osteoblast specific factor isoform 4 | | | XP_001085920.1 |
| 1241 | Peripheral-type benzodiazepine receptor-associated protein 1 (PRAX-1) | | | XP_001087623.1 |
| 1242 | peripherin | | | XP_001108977.1 |
| 1243 | peroxin1 isoform 2 | | | XP_001101055.1 |
| 1244 | Peroxiredoxin 1 | | | tr|Q9BGI4|Q9BGI4_BOVIN |
| 1245 | peroxiredoxin 1 isoform 3 | | | XP_001102281.1 |
| 1246 | peroxiredoxin 2 isoform 4 | | | XP_001109159.1 |
| 1247 | peroxiredoxin 6 | | | XP_001101473.1 |
| 1248 | Peroxiredoxin-4 (Prx-IV) (Thioredoxin peroxidase AO372)(AOE37-2) | | | XP_001087492.1 |
| 1249 | peroxisome proliferator-activated receptor gamma 1-b | | | NP_001028032.1 |
| 1250 | phenylalanine hydroxylase | | | XP_001094859.1 |
| 1251 | Phosphatase 1 regulatory subunit 7 protein-like protein (Fragment) | | | tr|A6ML40|A6ML40_CALJA |
| 1252 | phosphatase and actin regulator 2 isoform 4 | | | XP_001090568.1 |
| 1253 | phosphate cytidylyltransferase 2, ethanolamine | | | XP_001112535.1 |
| 1254 | Phosphatidylinositol glycan anchor biosynthesis, class T | | | tr|A4K2W4|A4K2W4_PONAB |
| 1255 | phosphatidylinositol N-acetylglucosaminyltransferase subunit A isoform 1 isoform 2 | | | XP_001100879.1 |
| 1256 | phosphatidylinositol transfer protein, beta | | | XP_001101197.1 |
| 1257 | phosphatidylinositol-3-phosphatase associated protein | | | XP_001109855.1 |
| 1258 | phosphatidylinositol-3-phosphate/phosphatidylinositol 5-kinase, type III isoform 2 isoform 4 | | | XP_001108540.1 |
| 1259 | phosphatidylinositol-4-phosphate 5-kinase type II alpha | | | XP_001099389.1 |
| 1260 | phosphatidylinositol-4-phosphate 5-kinase, type I, alpha isoform 3 | | | XP_001105678.1 |
| 1261 | phosphatidylinositol-4-phosphate 5-kinase, type I, beta isoform 2 | | | XP_001088891.1 |
| 1262 | phosphatidylinositol-4-phosphate 5-kinase, type II, beta | | | XP_001083994.1 |
| 1263 | phosphodiesterase 5A isoform 1 isoform 3 | | | XP_001099652.1 |
| 1264 | phosphodiesterase 8B isoform 1 | | | XP_001101740.1 |
| 1265 | phosphofructokinase, muscle | | | XP_001096244.1 |
| 1266 | phosphofructokinase, platelet, partial | | | XP_001118490.1 |
| 1267 | phosphofurin acidic cluster sorting protein 1 | | | XP_001111390.1 |
| 1268 | phosphoglucomutase 2-like 1 | | | XP_001115689.1 |
| 1269 | phosphoglycerate dehydrogenase isoform 3 | | | XP_001114128.1 |
| 1270 | phosphoglycerate dehydrogenase like 1 | | | XP_001091791.1 |
| 1271 | Phosphoglycerate kinase 1 | | | tr|Q3YAQ9|Q3YAQ9_MACMU |
| 1272 | phosphoglycerate kinase 1 isoform 4 | | | XP_001100787.1 |
| 1273 | phosphoglycerate kinase 2 | | | XP_001105356.1 |
| 1274 | Phosphoglycerate mutase 1 | | | tr|Q3YAR2|Q3YAR2_MACMU |
| 1275 | phosphoglycerate mutase 1 (brain) isoform 3 | | | XP_001104228.1 |
| 1276 | phosphoglycerate mutase 2 (muscle) | | | XP_001116220.1 |
| 1277 | phospholipase A1 member A isoform 2 | | | XP_001109981.1 |
| 1278 | phospholipase A2, group VII | | | XP_001103358.1 |
| 1279 | phospholipase A2-activating protein isoform 1 isoform 2 | | | XP_001105698.1 |
| 1280 | phospholipase C gamma 1 isoform a | | | XP_001087295.1 |
| 1281 | phospholipase C, beta 3 (phosphatidylinositol-specific) | | | XP_001115104.1 |
| 1282 | phospholipase C, gamma 2 (phosphatidylinositol-specific) isoform 2 | | | XP_001111717.1 |
| 1283 | phospholipase C-like 2 | | | XP_001084228.1 |
| 1284 | phospholipid scramblase 3 | | | XP_001118026.1 |
| 1285 | Phospholipid transfer protein-like protein (Fragment) | | | tr|A6MKU7|A6MKU7_CALJA |
| 1286 | phosphoribosylglycinamide formyltransferase, phosphoribosylglycinamide synthetase | | | XP_001093303.1 |
| 1287 | phosphorylated CTD interacting factor 1 isoform 1 | | | XP_001105319.1 |
| 1288 | phosphoserine aminotransferase isoform 1 isoform 2 | | | XP_001101670.1 |
| 1289 | phytanoyl-CoA hydroxylase interacting protein-like | | | XP_001090864.1 |
| 1290 | piggyBac transposable element derived 5 | | | XP_001117661.1 |
| 1291 | Pigment epithelium-derived factor precursor (PEDF) (EPC-1) | | | XP_001117361.1 |
| 1292 | plasma carboxypeptidase B2 isoform a preproprotein isoform 3 | | | XP_001097608.1 |
| 1293 | plasma membrane calcium ATPase 4 | | | NP_001028098.1 |
| 1294 | plasminogen activator inhibitor type 1, member 2 isoform 5 | | | XP_001109147.1 |
| 1295 | Platelet glycoprotein Ib alpha chain precursor (Glycoprotein Ibalpha) | | | XP_001117767.1 |
| 1296 | Platelet glycoprotein Ib beta chain precursor (GP-Ib beta) | | | XP_001105321.1 |
| 1297 | platelet-activating factor acetylhydrolase beta subunit | | | XP_001087675.1 |
| 1298 | platelet-activating factor acetylhydrolase, isoform Ib, gamma subunit 29kDa isoform 3 | | | XP_001105633.1 |
| 1299 | platelet-derived growth factor receptor beta isoform 2 | | | XP_001107595.1 |
| 1300 | pleckstrin homology domain containing, family A member 1 isoform 4 | | | XP_001103375.1 |
| 1301 | pleckstrin homology-like domain, family B, member 2 | | | XP_001097875.1 |
| 1302 | pleckstrin | | | XP_001094492.1 |
| 1303 | pleiomorphic adenoma gene-like 1 | | | XP_001098027.1 |
| 1304 | plexin domain containing 2 precursor isoform 2 | | | XP_001094803.1 |
| 1305 | Pol polypeptide | | | tr|Q5TYK3|Q5TYK3_SIVCZ |
| 1306 | pol, Truncated | | | tr|B2MIN0|B2MIN0_9HIV1 |
| 1307 | poly (ADP-ribose) polymerase family, member 14 | | | XP_001105869.1 |
| 1308 | poly (ADP-ribose) polymerase family, member 4, partial | | | XP_001110102.1 |
| 1309 | poly (ADP-ribose) polymerase family, member 4 | | | XP_001117681.1 |
| 1310 | poly (ADP-ribose) polymerase family, member 8 isoform 4 | | | XP_001094058.1 |
| 1311 | Poly [ADP-ribose] polymerase 4 (PARP-4), partial | | | XP_001119652.1 |
| 1312 | poly(A) binding protein, cytoplasmic 1 isoform 2 | | | XP_001098239.1 |
| 1313 | Poly(A) polymerase alpha (Fragment) | | | tr|Q4G3Z6|Q4G3Z6_MACMU |
| 1314 | polymerase (DNA directed), delta 1, catalytic subunit 125kDa isoform 2 | | | XP_001116065.1 |
| 1315 | polymerase (DNA directed), delta 3 | | | XP_001082315.1 |
| 1316 | polymerase (DNA directed), lambda isoform 10 | | | XP_001110701.1 |
| 1317 | Polymerase (Fragment) OS=Human immunodeficiency virus 1 | | | tr|Q5U6K8|Q5U6K8_9HIV1 |
| 1318 | Polymerase (Fragment) OS=Simian immunodeficiency virus (isolate CPZ GAB1) | | | tr|O90273|O90273_SIVCZ |
| 1319 | Polymerase (Fragment) OS=Simian immunodeficiency virus Qu | | | tr|Q70SH3|Q70SH3_SIVCZ |
| 1320 | polypeptide N-acetylgalactosaminyltransferase 3 | | | XP_001096023.1 |
| 1321 | Polyprotein (Fragment) | | | tr|A1KEB3|A1KEB3_9HIV2 |
| 1322 | polypyrimidine tract binding protein 2 isoform 6 | | | XP_001105316.1 |
| 1323 | potassium channel tetramerisation domain containing 3 | | | XP_001105750.1 |
| 1324 | potassium channel, subfamily K, member 5 | | | XP_001117117.1 |
| 1325 | potassium voltage-gated channel, shaker-related subfamily | | | XP_001090286.1 |
| 1326 | potassium voltage-gated channel, subfamily H, member 8 isoform 3 | | | XP_001087569.1 |
| 1327 | PRAME family member 6 | | | XP_001118879.1 |
| 1328 | Pregnancy zone protein precursor, partial | | | XP_001110832.1 |
| 1329 | Pregnancy-specific beta-1-glycoprotein 2 precursor (PSBG-2), partial | | | XP_001119729.1 |
| 1330 | Pregnancy-specific glycoprotein 69 (Fragment) | | | tr|Q9N164|Q9N164_PAPHA |
| 1331 | premature ovarian failure, 1B | | | XP_001083070.1 |
| 1332 | prematurely terminated mRNA decay factor-like, partial | | | XP_001117079.1 |
| 1333 | pre-mRNA cleavage factor I, 59 kDa subunit | | | XP_001082435.1 |
| 1334 | pre-mRNA processing factor 31 homolog | | | XP_001116111.1 |
| 1335 | presenilin 1 (Alzheimer disease 3) isoform 4 | | | XP_001088205.1 |
| 1336 | Probable phosphoglycerate mutase 4 | | | sp|Q8MKE8|PGAM4_PANTR |
| 1337 | processing of precursor 4, ribonuclease P/MRP subunit (S. cerevisiae) | | | XP_001084400.1 |
| 1338 | procollagen, type VI, alpha 2 | | | XP_001085988.1 |
| 1339 | procollagen-proline, 2-oxoglutarate 4-dioxygenase, alpha polypeptide I isoform 4 | | | XP_001104142.1 |
| 1340 | Programmed cell death ligand 1 | | | tr|A4GW17|A4GW17_CERTO |
| 1341 | Programmed cell death protein 6 (Probable calcium-binding protein ALG-2) (PMP41) (ALG-257) | | | XP_001119112.1 |
| 1342 | proliferating cell nuclear antigen isoform 2 | | | XP_001115756.1 |
| 1343 | proliferation-associated 2G4, 38kDa isoform 3 | | | XP_001114067.1 |
| 1344 | prolyl 4-hydroxylase, beta subunit, partial | | | XP_001116992.1 |
| 1345 | prosaposin isoform 8 | | | XP_001107170.1 |
| 1346 | Prostaglandin E synthase 3 (Cytosolic prostaglandin E2 synthase) (cPGES) isoform 3 | | | XP_001115374.1 |
| 1347 | Prostaglandin E synthase 3 | | | sp|Q6PWL5|TEBP_MACFA |
| 1348 | prostate specific antigen | | | NP_001036241.1 |
| 1349 | Protease | | | tr|A8JSS8|A8JSS8_9HIV1 |
| 1350 | Protease and reverse transcriptase | | | tr|B3CJV7|B3CJV7_9HIV1 |
| 1351 | protease, serine, 1 | | | NP_001040586.1 |
| 1352 | protease, serine, 1 (trypsin 1) | | | XP_001088295.1 |
| 1353 | protease, serine, 34 | | | XP_001118582.1 |
| 1354 | proteasomal ATPase (SUG1) | | | XP_001108909.1 |
| 1355 | proteasome (prosome, macropain) 26S subunit, non-ATPase, 14 | | | XP_001096177.1 |
| 1356 | proteasome (prosome, macropain) 26S subunit, ATPase 2, partial | | | XP_001118310.1 |
| 1357 | proteasome (prosome, macropain) 26S subunit, ATPase 2 | | | XP_001084593.1 |
| 1358 | proteasome (prosome, macropain) 26S subunit, non-ATPase, 13 isoform 2 | | | XP_001084506.1 |
| 1359 | proteasome (prosome, macropain) 26S subunit, non-ATPase, 6 isoform 6 | | | XP_001093028.1 |
| 1360 | proteasome (prosome, macropain) activator subunit 1 (PA28 alpha) | | | XP_001104073.1 |
| 1361 | proteasome (prosome, macropain) activator subunit 2 (PA28 beta) | | | XP_001112445.1 |
| 1362 | proteasome (prosome, macropain) subunit, alpha type, 3 isoform 1 | | | XP_001091058.1 |
| 1363 | proteasome (prosome, macropain) subunit, alpha type, 3 isoform 3 | | | XP_001091430.1 |
| 1364 | proteasome (prosome, macropain) subunit, alpha type, 8 isoform 1, partial | | | XP_001114742.1 |
| 1365 | proteasome (prosome, macropain) subunit, alpha type, 8 isoform 2 | | | XP_001098657.1 |
| 1366 | proteasome (prosome, macropain) subunit, beta type, 1 isoform 2 | | | XP_001084784.1 |
| 1367 | proteasome (prosome, macropain) subunit, beta type, 2 isoform 1 | | | XP_001109541.1 |
| 1368 | proteasome (prosome, macropain) subunit, beta type, 3 isoform 2 | | | XP_001084269.1 |
| 1369 | proteasome (prosome, macropain) subunit, beta type, 6 isoform 2 | | | XP_001096870.1 |
| 1370 | proteasome (prosome, macropain) subunit, beta type | | | XP_001109596.1 |
| 1371 | proteasome 26S ATPase subunit 3 isoform 5 | | | XP_001106497.1 |
| 1372 | proteasome 26S ATPase subunit 4 isoform 2 | | | XP_001090548.1 |
| 1373 | proteasome 26S ATPase subunit 6 | | | XP_001082028.1 |
| 1374 | proteasome 26S non-ATPase subunit 1 | | | XP_001113035.1 |
| 1375 | proteasome 26S non-ATPase subunit 11 | | | XP_001110049.1 |
| 1376 | proteasome 26S non-ATPase subunit 2 | | | XP_001093407.1 |
| 1377 | proteasome 26S non-ATPase subunit 3 isoform 5 | | | XP_001094629.1 |
| 1378 | proteasome 26S non-ATPase subunit 7 | | | XP_001102147.1 |
| 1379 | proteasome beta 10 subunit proprotein | | | XP_001095570.1 |
| 1380 | proteasome beta 4 subunit isoform 3 | | | XP_001108305.1 |
| 1381 | proteasome beta 5 subunit | | | XP_001105158.1 |
| 1382 | proteasome beta 7 subunit | | | XP_001083368.1 |
| 1383 | proteasome beta 8 subunit isoform E2 proprotein | | | XP_001115562.1 |
| 1384 | proteasome beta 9 subunit isoform 2 proprotein | | | XP_001108087.1 |
| 1385 | Proteasome subunit alpha type 1 (Proteasome component C2) (Macropain subunit C2)(PROS-30) | | | XP_001089332.1 |
| 1386 | Proteasome subunit alpha type 1-like protein (Fragment) | | | tr|A6MJW2|A6MJW2_CALJA |
| 1387 | Proteasome subunit alpha type 4 (Proteasome component C9) isoform 3 | | | XP_001108132.1 |
| 1388 | Proteasome subunit alpha type 5 (Proteasome zeta chain) | | | XP_001090512.1 |
| 1389 | Proteasome subunit alpha type 6 (Proteasome iota chain) isoform 1 | | | XP_001095366.1 |
| 1390 | Proteasome subunit alpha type 6 (Proteasome iota chain) isoform 3 | | | XP_001085957.1 |
| 1391 | Proteasome subunit beta type 5-like protein | | | tr|A6MLB5|A6MLB5_CALJA |
| 1392 | Proteasome subunit beta type 7-like protein | | | tr|A6MLE5|A6MLE5_CALJA |
| 1393 | Proteasome subunit, beta type 6 | | | tr|Q3YAI3|Q3YAI3_MACMU |
| 1394 | protein arginine methyltransferase 5 isoform a isoform 4 | | | XP_001103964.1 |
| 1395 | Protein C14orf102 homolog isoform 4 | | | XP_001090586.1 |
| 1396 | Protein C20orf4 homolog isoform 2 | | | XP_001096526.1 |
| 1397 | protein disulfide isomerase-associated 3 precursor isoform 4 | | | XP_001109119.1 |
| 1398 | protein disulfide isomerase-associated 6 | | | XP_001095159.1 |
| 1399 | Protein disulfide-isomerase A4 precursor (Protein ERp-72) (ERp72), partial | | | XP_001118793.1 |
| 1400 | Protein FAM40A isoform 2 | | | XP_001099183.1 |
| 1401 | Protein FAM61B | | | XP_001090063.1 |
| 1402 | protein inhibitor of activated STAT X isoform beta isoform 6 | | | XP_001085703.1 |
| 1403 | Protein kinase C iota (Fragment) | | | tr|Q6UIN2|Q6UIN2_PANTR |
| 1404 | protein kinase C, iota | | | XP_001089400.1 |
| 1405 | protein kinase PKNbeta | | | XP_001110500.1 |
| 1406 | protein kinase, cAMP-dependent, catalytic, beta isoform 10 | | | XP_001106097.1 |
| 1407 | protein kinase, cAMP-dependent, regulatory, type I, alpha (tissue specific extinguisher 1) | | | XP_001112514.1 |
| 1408 | protein kinase, DNA-activated, catalytic polypeptide isoform 2 | | | XP_001100610.1 |
| 1409 | protein phosphatase 1, catalytic subunit, alpha | | | XP_001117941.1 |
| 1410 | protein phosphatase 1, catalytic subunit, gamma isoform isoform 2 | | | XP_001108364.1 |
| 1411 | protein phosphatase 1, regulatory (inhibitor) subunit 12A isoform 7 | | | XP_001086467.1 |
| 1412 | protein phosphatase 1, regulatory subunit 7 | | | XP_001090766.1 |
| 1413 | protein phosphatase 1G isoform 6 | | | XP_001096065.1 |
| 1414 | protein phosphatase 2, regulatory subunit B, delta isoform 1 isoform 7 | | | XP_001091436.1 |
| 1415 | protein phosphatase 3 (formerly 2B) (calcineurin A alpha) | | | XP_001108659.1 |
| 1416 | protein phosphatase 3 (formerly 2B), catalytic subunit, alpha isoform (calcineurin A alpha) isoform 4 | | | XP_001108612.1 |
| 1417 | protein phosphatase methylesterase-1 isoform 1 | | | XP_001115651.1 |
| 1418 | Protein SET (Phosphatase 2A inhibitor I2PP2A) (I-2PP2A) isoform 2 | | | XP_001110579.1 |
| 1419 | protein tyrosine phosphatase, non-receptor type 1 isoform 3 | | | XP_001096290.1 |
| 1420 | protein tyrosine phosphatase, non-receptor type 2 isoform 2 | | | XP_001118328.1 |
| 1421 | protein tyrosine phosphatase, non-receptor type 23 | | | XP_001100428.1 |
| 1422 | protein tyrosine phosphatase, non-receptor type 6 isoform 2 | | | XP_001110915.1 |
| 1423 | protein tyrosine phosphatase, receptor type, V | | | XP_001095914.1 |
| 1424 | protein tyrosine phosphatase, receptor-type, zeta1 | | | XP_001083149.1 |
| 1425 | proteoglycan 1, secretory granule isoform 2 | | | XP_001110781.1 |
| 1426 | proteoglycan 4 | | | XP_001107843.1 |
| 1427 | proteolipid protein 2 (colonic epithelium-enriched) isoform 1 | | | XP_001106005.1 |
| 1428 | Prothrombin precursor (Coagulation factor II), partial | | | XP_001111947.1 |
| 1429 | protocadherin 12 | | | XP_001094671.1 |
| 1430 | protocadherin 15 precursor | | | XP_001098443.1 |
| 1431 | protocadherin 18 precursor | | | XP_001086925.1 |
| 1432 | protocadherin 7 isoform 4 | | | XP_001085728.1 |
| 1433 | protocadherin beta 16 | | | XP_001091535.1 |
| 1434 | Proto-oncogene tyrosine-protein kinase LCK (p56-LCK), partial | | | XP_001109630.1 |
| 1435 | proto-oncogene tyrosine-protein kinase SRC isoform 2 | | | XP_001092301.1 |
| 1436 | PRP19/PSO4 pre-mRNA processing factor 19 homolog | | | XP_001084826.1 |
| 1437 | pseudouridylate synthase 1 isoform 1 | | | XP_001113470.1 |
| 1438 | purine nucleoside phosphorylase | | | XP_001104622.1 |
| 1439 | Purine nucleoside phosphorylase-like protein (Fragment) | | | tr|A6MJU5|A6MJU5_CALJA |
| 1440 | purinergic receptor P2X, ligand-gated ion channel, 2 isoform 7 | | | XP_001082740.1 |
| 1441 | Putative uncharacterized protein GW128 | | | tr|Q8SPH7|Q8SPH7_MACFA |
| 1442 | PWP2 periodic tryptophan protein homolog | | | XP_001118393.1 |
| 1443 | pyridoxal kinase isoform 2 | | | XP_001104678.1 |
| 1444 | pyrophosphatase 1 | | | XP_001107614.1 |
| 1445 | pyruvate dehydrogenase kinase, isozyme 1 | | | XP_001086316.1 |
| 1446 | pyruvate kinase 3 isoform 9 | | | XP_001091427.1 |
| 1447 | pyruvate kinase 3 | | | XP_001099473.1 |
| 1448 | pyruvate kinase, liver and RBC isoform 1 | | | XP_001112902.1 |
| 1449 | quiescin Q6 isoform a | | | XP_001111489.1 |
| 1450 | quiescin Q6-like 1 | | | XP_001117970.1 |
| 1451 | Rab geranylgeranyltransferase, alpha subunit | | | XP_001104317.1 |
| 1452 | RAB11a, member RAS oncogene family | | | XP_001103732.1 |
| 1453 | rab11-family interacting protein 3 | | | XP_001118464.1 |
| 1454 | RAB1B, member RAS oncogene family | | | XP_001118047.1 |
| 1455 | RAB2, member RAS oncogene family isoform 1 | | | XP_001089665.1 |
| 1456 | RAB2B protein | | | XP_001096730.1 |
| 1457 | RAB33B, member RAS oncogene family | | | XP_001088193.1 |
| 1458 | RAB3D, member RAS oncogene family | | | XP_001104198.1 |
| 1459 | RAB5B, member RAS oncogene family | | | XP_001096821.1 |
| 1460 | RAB5-interacting protein | | | XP_001098255.1 |
| 1461 | RAB6A, member RAS oncogene family isoform a | | | XP_001115437.1 |
| 1462 | RAD23 homolog B (S. cerevisiae) isoform 5 | | | XP_001109100.1 |
| 1463 | RAD9 homolog B | | | XP_001099777.1 |
| 1464 | radixin | | | XP_001104955.1 |
| 1465 | Ral-GDS related protein Rgr isoform 2 | | | XP_001097394.1 |
| 1466 | RAN binding protein 5 | | | XP_001089390.1 |
| 1467 | RAN binding protein 5 isoform 4 | | | XP_001089501.1 |
| 1468 | RAN, member RAS oncogene family | | | XP_001104422.1 |
| 1469 | RANBP2-like and GRIP domain-containing protein 4 | | | sp|Q7Z3J3|RGPD4_HUMAN |
| 1470 | RANBP2-like and GRIP domain-containing protein 7 | | | sp|Q9H0B2|RGPD7_HUMAN |
| 1471 | RAP1A, member of RAS oncogene family (Fragment) | | | tr|Q3YAJ2|Q3YAJ2_MACMU |
| 1472 | RAP1B, member of RAS oncogene family | | | XP_001082451.1 |
| 1473 | RaP2 interacting protein 8 | | | XP_001100710.1 |
| 1474 | rap2 interacting protein x | | | XP_001097881.1 |
| 1475 | ras homolog gene family, member A | | | XP_001094831.1 |
| 1476 | ras homolog gene family, member U | | | XP_001083463.1 |
| 1477 | Ras protein-specific guanine nucleotide-releasing factor 2, partial | | | XP_001118872.1 |
| 1478 | Ras-GTPase activating protein SH3 domain-binding protein 2 isoform a | | | XP_001100686.1 |
| 1479 | Ras-GTPase-activating protein SH3-domain-binding protein isoform 9 | | | XP_001110819.1 |
| 1480 | RAS-related C3 botulinum substrate 3 | | | XP_001113336.1 |
| 1481 | ras-related C3 botulinum toxin substrate 1 isoform Rac1b | | | XP_001109586.1 |
| 1482 | ras-related C3 botulinum toxin substrate 2 (rho family, small GTP binding protein Rac2) | | | XP_001086228.1 |
| 1483 | Ras-related GTP-binding protein ragA | | | XP_001101910.1 |
| 1484 | Ras-related protein Rab-12 (Rab-13) | | | XP_001118552.1 |
| 1485 | Ras-related protein Rab-7 | | | XP_001095709.1 |
| 1486 | receptor accessory protein 5 | | | XP_001082843.1 |
| 1487 | receptor for egg jelly-like protein | | | XP_001110678.1 |
| 1488 | recombining binding protein suppressor of hairless isoform 4 isoform 3 | | | XP_001084065.1 |
| 1489 | RecQ protein-like isoform 1 | | | XP_001094207.1 |
| 1490 | reelin isoform a isoform 2 | | | XP_001087945.1 |
| 1491 | regucalcin isoform 3 | | | XP_001090835.1 |
| 1492 | regulatory factor X domain containing 1 | | | XP_001110999.1 |
| 1493 | regulatory factor X, 5 isoform 1 isoform 2 | | | XP_001090581.1 |
| 1494 | Rep protein (DNA binding trs helicase) | | | tr|Q9YJC1|Q9YJC1_9VIRU |
| 1495 | replication protein A2 | | | tr|Q3YAR6|Q3YAR6_MACMU |
| 1496 | restin-like 2 isoform 3 | | | XP_001103267.1 |
| 1497 | reticulon 4 | | | XP_001112090.1 |
| 1498 | Reticulon-3 (Neuroendocrine-specific protein-like 2) (NSP-like protein II) (NSPLII) isoform 9 | | | XP_001115762.1 |
| 1499 | retinoblastoma binding protein 4 isoform 6 | | | XP_001104415.1 |
| 1500 | retinoblastoma binding protein 7 isoform 4 | | | XP_001103800.1 |
| 1501 | retinoblastoma binding protein 8 isoform a | | | XP_001093018.1 |
| 1502 | retinoic acid receptor, beta isoform 2 | | | XP_001092452.1 |
| 1503 | retinol binding protein 2, cellular | | | XP_001113494.1 |
| 1504 | retrotransposon-like 1 | | | XP_001110319.1 |
| 1505 | Rev | | | tr|Q1A254|Q1A254_SIVCZ |
| 1506 | REV3-like, catalytic subunit of DNA polymerase zeta | | | XP_001086055.1 |
| 1507 | Reverse transcriptase | | | tr|Q7SL19|Q7SL19_9HIV1 |
| 1508 | Rho GDP dissociation inhibitor (GDI) alpha isoform 2 | | | XP_001112043.1 |
| 1509 | Rho GDP dissociation inhibitor (GDI) beta | | | XP_001090606.1 |
| 1510 | Rho GTPase activating protein 1 | | | XP_001101907.1 |
| 1511 | Rho GTPase activating protein 20 | | | XP_001098860.1 |
| 1512 | Rho guanine nucleotide exchange factor (GEF) 19 | | | XP_001092728.1 |
| 1513 | ribonuclease III, nuclear | | | XP_001085611.1 |
| 1514 | ribonuclease P | | | XP_001104161.1 |
| 1515 | ribonuclease, RNase A family, 11 (non-active) | | | XP_001090818.1 |
| 1516 | ribonucleoside-diphosphate reductase M1 chain isoform 1 | | | XP_001113010.1 |
| 1517 | ribonucleoside-diphosphate reductase M1 chain isoform 2 | | | XP_001113133.1 |
| 1518 | ribophorin II precursor | | | XP_001097464.1 |
| 1519 | ribosomal protein L10 isoform 1 | | | XP_001109783.1 |
| 1520 | ribosomal protein L10 | | | XP_001086567.1 |
| 1521 | ribosomal protein L10a | | | XP_001111950.1 |
| 1522 | ribosomal protein L13 | | | XP_001092801.1 |
| 1523 | Ribosomal protein L13A | | | tr|B5MBT6|B5MBT6_MACMU |
| 1524 | ribosomal protein L13a | | | XP_001115079.1 |
| 1525 | ribosomal protein L15 | | | XP_001102774.1 |
| 1526 | ribosomal protein L18 | | | XP_001092745.1 |
| 1527 | ribosomal protein L18a | | | XP_001108577.1 |
| 1528 | ribosomal protein L19 | | | XP_001110195.1 |
| 1529 | ribosomal protein L21 | | | XP_001110717.1 |
| 1530 | ribosomal protein L24 isoform 1 | | | XP_001097920.1 |
| 1531 | ribosomal protein L24 | | | XP_001106128.1 |
| 1532 | ribosomal protein L27 isoform 3 | | | XP_001112771.1 |
| 1533 | ribosomal protein L27a | | | XP_001106319.1 |
| 1534 | ribosomal protein L28 | | | XP_001113291.1 |
| 1535 | ribosomal protein L3 isoform a | | | XP_001095608.1 |
| 1536 | ribosomal protein L3 isoform a isoform 2 | | | XP_001106805.1 |
| 1537 | ribosomal protein L30 | | | XP_001083674.1 |
| 1538 | ribosomal protein L31 | | | XP_001105162.1 |
| 1539 | ribosomal protein L35a | | | XP_001082551.1 |
| 1540 | ribosomal protein L4 isoform 2 | | | XP_001110697.1 |
| 1541 | ribosomal protein L5, partial | | | XP_001117260.1 |
| 1542 | ribosomal protein L9 isoform 2 | | | XP_001116116.1 |
| 1543 | ribosomal protein L9 | | | XP_001094980.1 |
| 1544 | Ribosomal protein P1 | | | tr|Q6V8L6|Q6V8L6_MACRA |
| 1545 | Ribosomal protein S11 | | | tr|Q3YAQ2|Q3YAQ2_MACMU |
| 1546 | ribosomal protein S11 isoform 2 | | | XP_001099340.1 |
| 1547 | ribosomal protein S13 | | | XP_001083808.1 |
| 1548 | ribosomal protein S14 | | | XP_001099589.1 |
| 1549 | ribosomal protein S15a | | | XP_001115032.1 |
| 1550 | ribosomal protein S18 isoform 3 | | | XP_001099240.1 |
| 1551 | ribosomal protein S2 | | | XP_001116842.1 |
| 1552 | ribosomal protein S23 | | | XP_001108572.1 |
| 1553 | ribosomal protein S24 isoform 3 | | | XP_001103378.1 |
| 1554 | ribosomal protein S24 | | | XP_001105354.1 |
| 1555 | ribosomal protein S27 | | | XP_001084710.1 |
| 1556 | ribosomal protein S3a | | | XP_001099771.1 |
| 1557 | ribosomal protein S8 isoform 1 | | | XP_001098975.1 |
| 1558 | Ribosomal protein SA | | | tr|Q3YAI0|Q3YAI0_MACMU |
| 1559 | Ribosome biogenesis protein BMS1 homolog, partial | | | XP_001119032.1 |
| 1560 | ring finger protein 121 isoform 1 isoform 4 | | | XP_001113850.1 |
| 1561 | ring finger protein 149 | | | XP_001106945.1 |
| 1562 | ring finger protein 151 | | | XP_001082447.1 |
| 1563 | ring finger protein 165 | | | XP_001090387.1 |
| 1564 | ring finger protein 29 isoform 2 | | | XP_001100542.1 |
| 1565 | ring finger protein 36 isoform b isoform 4 | | | XP_001110863.1 |
| 1566 | RNA binding motif protein 12 | | | NP_001028012.1 |
| 1567 | RNA binding motif protein 22 isoform 1 | | | XP_001108809.1 |
| 1568 | RNA binding motif protein 4 isoform 3 | | | XP_001109150.1 |
| 1569 | RNA polymerase I subunit | | | XP_001096103.1 |
| 1570 | RNA polymerase II subunit 5-mediating protein (RPB5-mediating protein) isoform 2 | | | XP_001085232.1 |
| 1571 | RuvB-like 2-like protein | | | tr|A6MK39|A6MK39_CALJA |
| 1572 | RYK receptor-like tyrosine kinase | | | XP_001112355.1 |
| 1573 | S100 calcium binding protein A1 isoform 2 | | | XP_001111052.1 |
| 1574 | S100 calcium binding protein A8 (calgranulin A) isoform 2 | | | XP_001110492.1 |
| 1575 | S100 calcium binding protein A9 (calgranulin B) isoform 2 | | | XP_001110408.1 |
| 1576 | S-adenosylhomocysteine hydrolase | | | XP_001104495.1 |
| 1577 | S-adenosylhomocysteine hydrolase-like 1 | | | XP_001098170.1 |
| 1578 | SAM domain- and HD domain-containing protein 1 | | | XP_001097562.1 |
| 1579 | SAPS domain family member 2 | | | XP_001116314.1 |
| 1580 | SAR1 gene homolog A (S. cerevisiae) isoform 2 | | | XP_001109714.1 |
| 1581 | SAR1 gene homolog B (S. cerevisiae) isoform 4 | | | XP_001109899.1 |
| 1582 | sarcoma antigen NY-SAR-41, partial | | | XP_001102806.1 |
| 1583 | SCY1-like 2 protein isoform 3 | | | XP_001089605.1 |
| 1584 | SEC13-like 1 isoform b isoform 8 | | | XP_001090208.1 |
| 1585 | SEC13-related protein-like protein (Fragment) | | | tr|A6MLE6|A6MLE6_CALJA |
| 1586 | secreted protein, acidic, cysteine-rich (osteonectin) | | | XP_001101364.1 |
| 1587 | secretoglobin, family 1C, member 1 | | | XP_001082613.1 |
| 1588 | selenophosphate synthetase | | | XP_001086299.1 |
| 1589 | Semenogelin I isoform a preproprotein | | | tr|A4K2R7|A4K2R7_GORGO |
| 1590 | semenogelin II | | | XP_001109304.1 |
| 1591 | septin 11 isoform 2 | | | XP_001093370.1 |
| 1592 | septin 2 | | | XP_001109436.1 |
| 1593 | septin 6 | | | XP_001082728.1 |
| 1594 | septin 8 isoform 2 | | | XP_001104760.1 |
| 1595 | Septin-9 (MLL septin-like fusion protein), partial | | | XP_001116653.1 |
| 1596 | Septin-9 (SL3-3 integration site 1 protein), partial | | | XP_001116647.1 |
| 1597 | Ser/Arg-related nuclear matrix protein | | | XP_001082460.1 |
| 1598 | serine (or cysteine) proteinase inhibitor, clade A , member 10 isoform 2 | | | XP_001097853.1 |
| 1599 | serine (or cysteine) proteinase inhibitor, clade A (alpha-1 antiproteinase, antitrypsin), member 12 | | | XP_001099453.1 |
| 1600 | serine (or cysteine) proteinase inhibitor, clade A (alpha-1 antiproteinase, antitrypsin), member 5 | | | NP_001038198.1 |
| 1601 | serine (or cysteine) proteinase inhibitor, clade A, member 7 | | | XP_001088790.1 |
| 1602 | serine (or cysteine) proteinase inhibitor, clade B (ovalbumin), member 12 isoform 2 | | | XP_001090546.1 |
| 1603 | serine (or cysteine) proteinase inhibitor, clade B (ovalbumin), member 3 isoform 4 | | | XP_001091267.1 |
| 1604 | serine (or cysteine) proteinase inhibitor, clade B (ovalbumin), member 5 | | | XP_001090087.1 |
| 1605 | serine (or cysteine) proteinase inhibitor, clade B (ovalbumin), member 9 | | | XP_001091156.1 |
| 1606 | serine (or cysteine) proteinase inhibitor, clade C (antithrombin), member 1 | | | NP_001098053.1 |
| 1607 | serine (or cysteine) proteinase inhibitor, clade H, member 1 | | | XP_001084827.1 |
| 1608 | serine hydroxymethyltransferase 1 (soluble) isoform 1 isoform 8 | | | XP_001096982.1 |
| 1609 | serine hydroxymethyltransferase 2 (mitochondrial) isoform 10 | | | XP_001115892.1 |
| 1610 | serine threonine kinase 39 (STE20/SPS1 homolog, yeast) isoform 2 | | | XP_001102299.1 |
| 1611 | serine/threonine kinase receptor associated protein isoform 2 | | | XP_001092419.1 |
| 1612 | serine/threonine protein kinase MASK | | | XP_001096161.1 |
| 1613 | Serine/threonine-protein phosphatase 2A catalytic subunit alpha isoform (PP2A-alpha) isoform 1 | | | XP_001107958.1 |
| 1614 | Serine/threonine-protein phosphatase 2A catalytic subunit beta isoform-like protein | | | tr|A6MK11|A6MK11_CALJA |
| 1615 | Serine/threonine-protein phosphatase 4 catalytic subunit (PP4C) | | | XP_001108500.1 |
| 1616 | Serine/threonine-protein phosphatase PP1-beta catalytic subunit (PP-1B) isoform 6 | | | XP_001102208.1 |
| 1617 | serpin peptidase inhibitor, clade B (ovalbumin), member 6 isoform 7 | | | XP_001093458.1 |
| 1618 | SERPINE1 mRNA binding protein 1 isoform 5 | | | XP_001095071.1 |
| 1619 | seryl-aminoacyl-tRNA synthetase 1 | | | XP_001089358.1 |
| 1620 | SET domain and mariner transposase fusion gene | | | XP_001089198.1 |
| 1621 | SET translocation | | | tr|Q3YAR7|Q3YAR7_MACMU |
| 1622 | seven in absentia homolog 1 (Drosophila) | | | XP_001083570.1 |
| 1623 | Sex-determining protein SRY | | | tr|A6QKW1|A6QKW1_CERAG |
| 1624 | SH3 and cysteine rich domain | | | XP_001101503.1 |
| 1625 | SH3 domain and tetratricopeptide repeats 1 | | | XP_001112519.1 |
| 1626 | SH3 domain protein D19 isoform 4 | | | XP_001083158.1 |
| 1627 | SH3 multiple domains 1 | | | XP_001113943.1 |
| 1628 | SH3-binding domain kinase 1 | | | XP_001095822.1 |
| 1629 | Shaw-related voltage-gated potassium channel protein 4 isoform a isoform 4 | | | XP_001099902.1 |
| 1630 | short-chain dehydrogenase/reductase isoform 4 | | | XP_001111397.1 |
| 1631 | SHROOM1-like protein | | | tr|A6ML32|A6ML32_CALJA |
| 1632 | Shwachman-Bodian-Diamond syndrome | | | XP_001085593.1 |
| 1633 | sialoadhesin precursor | | | XP_001115256.1 |
| 1634 | signal recognition particle 72kDa isoform 3 | | | XP_001085253.1 |
| 1635 | signal transducer and activator of transcription 3 | | | XP_001109505.1 |
| 1636 | signaling lymphocytic activation molecule family member 1 | | | XP_001117605.1 |
| 1637 | signal-regulatory protein beta 1 | | | XP_001113542.1 |
| 1638 | single-stranded DNA binding protein 1 | | | XP_001084179.1 |
| 1639 | Skin ASpartic Protease | | | XP_001098191.1 |
| 1640 | Small envelope protein | | | tr|Q5MNY9|Q5MNY9_9CORO |
| 1641 | small glutamine-rich tetratricopeptide | | | XP_001117755.1 |
| 1642 | small inducible cytokine subfamily E, member 1 | | | XP_001083775.1 |
| 1643 | small nuclear ribonucleoparticle-associated protein isoform 6 | | | XP_001107219.1 |
| 1644 | small nuclear ribonucleoprotein D2 | | | XP_001108236.1 |
| 1645 | small nuclear ribonucleoprotein E isoform 1 | | | XP_001099502.1 |
| 1646 | small nuclear RNA activating complex, polypeptide 1 | | | XP_001099253.1 |
| 1647 | small optic lobes | | | XP_001085587.1 |
| 1648 | SMC1 structural maintenance of chromosomes 1-like 1 | | | XP_001091228.1 |
| 1649 | SMC4 structural maintenance of chromosomes 4-like 1 isoform 14 | | | XP_001099428.1 |
| 1650 | Smith-Magenis syndrome chromosome region, candidate 8 | | | XP_001095753.1 |
| 1651 | snail homolog 1 (Drosophila) | | | XP_001102091.1 |
| 1652 | SNF2 histone linker PHD RING helicase | | | XP_001086641.1 |
| 1653 | soc-2 suppressor of clear homolog isoform 3 | | | XP_001087610.1 |
| 1654 | soluble mannose-binding lectin | | | NP_001099005.1 |
| 1655 | solute carrier family 12 (sodium/chloride transporters), member 3 isoform 2 | | | XP_001093708.1 |
| 1656 | solute carrier family 16, member 3 isoform 1 | | | XP_001113138.1 |
| 1657 | solute carrier family 2 (facilitated glucose transporter), member 1 isoform 4 | | | XP_001090748.1 |
| 1658 | solute carrier family 2 (facilitated glucose transporter), member 3 isoform 6 | | | XP_001113218.1 |
| 1659 | solute carrier family 20, member 2 isoform 3 | | | XP_001098131.1 |
| 1660 | solute carrier family 25 member 3 | | | XP_001083690.1 |
| 1661 | solute carrier family 25, member A6 | | | XP_001114519.1 |
| 1662 | solute carrier family 26, member 7 isoform b | | | XP_001085189.1 |
| 1663 | solute carrier family 5 (sodium/glucose cotransporter), member 10 | | | XP_001097568.1 |
| 1664 | solute carrier family 6 (neurotransmitter transporter, betaine/GABA), member 12 isoform 5 | | | XP_001093547.1 |
| 1665 | solute carrier family 8 member 3 isoform D precursor isoform 8 | | | XP_001110902.1 |
| 1666 | sorting nexin 1 | | | XP_001106400.1 |
| 1667 | spaghetti CG13570-PA isoform 2 | | | XP_001097288.1 |
| 1668 | sparc/osteonectin, cwcv and kazal-like domains proteoglycan (testican) 2 isoform 1 | | | XP_001105299.1 |
| 1669 | SPARC-like 1 isoform 3 | | | XP_001097743.1 |
| 1670 | spastin isoform 1, partial | | | XP_001115847.1 |
| 1671 | spectrin, alpha, erythrocytic 1 (elliptocytosis 2) isoform 2 | | | XP_001117115.1 |
| 1672 | spectrin, beta, non-erythrocytic 1 isoform 4 | | | XP_001114804.1 |
| 1673 | spermatogenesis associated 21 | | | XP_001093074.1 |
| 1674 | spermatogenesis associated 5-like 1 isoform 3 | | | XP_001111759.1 |
| 1675 | spermatogenesis associated factor SPAF isoform 3 | | | XP_001104133.1 |
| 1676 | spermidine synthase | | | XP_001102838.1 |
| 1677 | sphingosine-1-phosphate lyase 1 | | | XP_001106861.1 |
| 1678 | spindle assembly associated Sfi1 homolog isoform b isoform 6 | | | XP_001111206.1 |
| 1679 | spinster-like, partial | | | XP_001116179.1 |
| 1680 | splicing factor 3a, subunit 1, 120kDa isoform 3 | | | XP_001109090.1 |
| 1681 | splicing factor 3a, subunit 2 | | | XP_001108676.1 |
| 1682 | splicing factor 3B, 14 kDa subunit | | | XP_001098393.1 |
| 1683 | splicing factor 3b, subunit 1 isoform 1 | | | XP_001086317.1 |
| 1684 | splicing factor 3b, subunit 3 | | | XP_001107025.1 |
| 1685 | Splicing factor U2AF 35 kDa subunit (U2 auxiliary factor 35 kDa subunit) | | | XP_001118538.1 |
| 1686 | splicing factor, arginine/serine-rich 1 (ASF/SF2) | | | XP_001103473.1 |
| 1687 | splicing factor, arginine/serine-rich 14 | | | XP_001114911.1 |
| 1688 | Splicing factor, arginine/serine-rich 2 (Splicing factor SC35) (SC-35) isoform 3 | | | XP_001106051.1 |
| 1689 | Splicing factor, arginine/serine-rich 3 (Pre-mRNA-splicing factor SRP20) (X16 protein) | | | XP_001112817.1 |
| 1690 | splicing factor, arginine/serine-rich 4 isoform 4 | | | XP_001113540.1 |
| 1691 | splicing factor, arginine/serine-rich 5 isoform 4 | | | XP_001110146.1 |
| 1692 | splicing factor, arginine/serine-rich 7 | | | XP_001102729.1 |
| 1693 | SPT2, Suppressor of Ty, domain containing 1 | | | XP_001082108.1 |
| 1694 | squamous cell carcinoma antigen recognized by T cells 1, partial | | | XP_001114941.1 |
| 1695 | SRY (sex determining region Y)-box 11 | | | XP_001098988.1 |
| 1696 | | | SRY-box 17 isoform 2 | XP_001083536.1 |
| 1697 | | SSXT protein (Synovial sarcoma, translocated to X chromosome) (SYT protein) | | XP_001098462.1 |
| 1698 | | staphylococcal nuclease domain containing 1 | | XP_001088001.1 |
| 1699 | | START domain containing 8 | | XP_001082337.1 |
| 1700 | | Stathmin (Phosphoprotein p19) (pp19) (Oncoprotein 18) (Op18) (Protein Pr22) | | XP_001114361.1 |
| 1701 | | sterolin 2 | | XP_001111321.1 |
| 1702 | | stomatin isoform 1 | | XP_001090536.1 |
| 1703 | | stomatin isoform 2 | | XP_001090776.1 |
| 1704 | | stratifin isoform 2 | | XP_001110323.1 |
| 1705 | | striatin, calmodulin binding protein | | XP_001107812.1 |
| 1706 | | stromal antigen 3 | | XP_001102876.1 |
| 1707 | | stromal cell-derived factor 2-like 1 isoform 2 | | XP_001087778.1 |
| 1708 | | structure specific recognition protein 1 | | XP_001103334.1 |
| 1709 | | sulfotransferase family, cytosolic, 1C, member 1 isoform 5 | | XP_001082748.1 |
| 1710 | | sulfotransferase family, cytosolic, 1C, member 2 | | XP_001083001.1 |
| 1711 | | sulfotransferase, estrogen-preferring | | XP_001106765.1 |
| 1712 | | SUMO-1 activating enzyme subunit 2 isoform 7 | | XP_001091974.1 |
| 1713 | | Superoxide dismutase [Cu-Zn] | | sp|Q8HXQ4|SODC_PONPY |
| 1714 | | superoxide dismutase 1, soluble | | NP_001027976.1 |
| 1715 | | supervillin isoform 2 | | XP_001083894.1 |
| 1716 | | suppressor of variegation 3-9 homolog 1 isoform 1 | | XP_001099361.1 |
| 1717 | | suppressor of variegation 4-20 homolog 2 isoform 2 | | XP_001086620.1 |
| 1718 | | Surface glycoprotein (Fragment) | | tr|Q8QDI1|Q8QDI1_SIVCZ |
| 1719 | | surface glycoprotein, Ig superfamily member isoform 2 | | XP_001112218.1 |
| 1720 | | sushi-repeat-containing protein, X-linked isoform 2 | | XP_001084091.1 |
| 1721 | | SWI/SNF-related matrix-associated actin-dependent regulator of chromatin a5 isoform 2 | | XP_001093597.1 |
| 1722 | | SWI/SNF-related matrix-associated actin-dependent regulator of chromatin a-like 1 | | XP_001086594.1 |
| 1723 | | SWI/SNF-related matrix-associated actin-dependent regulator of chromatin d2 | | XP_001108960.1 |
| 1724 | | SWI/SNF-related matrix-associated actin-dependent regulator of chromatin e1 isoform 3 | | XP_001099528.1 |
| 1725 | | synaptogyrin 2 isoform 3 | | XP_001107757.1 |
| 1726 | | Synaptonemal complex protein 1 (SCP-1) isoform 2 | | XP_001086113.1 |
| 1727 | | synaptotagmin XV isoform a | | XP_001083207.1 |
| 1728 | | syntaxin binding protein 2 | | XP_001097163.1 |
| 1729 | | syntenin isoform 2 isoform 4 | | XP_001088209.1 |
| 1730 | | syntenin isoform 3 isoform 3 | | XP_001088096.1 |
| 1731 | | syntrophin, gamma 1 isoform 2 | | XP_001101712.1 |
| 1732 | | T02H6.3 | | XP_001104315.1 |
| 1733 | | T03G11.3 isoform 1 | | XP_001088418.1 |
| 1734 | | T03G11.6 | | XP_001086688.1 |
| 1735 | | talin 1 | | XP_001084941.1 |
| 1736 | | talin 2 isoform 2 | | XP_001101705.1 |
| 1737 | | Tat | | tr|Q00CE1|Q00CE1_9HIV1 |
| 1738 | | TATA element modulatory factor 1 isoform 2 | | XP_001088711.1 |
| 1739 | | TatD DNase domain containing 1 isoform 2 | | XP_001101992.1 |
| 1740 | | TBC1 domain family, member 15 isoform 4 | | XP_001117539.1 |
| 1741 | | T-cell leukemia virus enhancer factor isoform 2 | | XP_001113848.1 |
| 1742 | | T-cell receptor alpha chain C region | | XP_001098834.1 |
| 1743 | | T-cell receptor beta chain C region | | XP_001091216.1 |
| 1744 | | T-cell receptor beta chain V region 86T1 precursor | | XP_001085775.1 |
| 1745 | | T-cell receptor interacting molecule | | XP_001101777.1 |
| 1746 | | T-cell surface glycoprotein CD3 gamma chain precursor (T-cell receptor T3 gamma chain) | | XP_001093643.1 |
| 1747 | | T-complex protein 1 isoform 6 | | XP_001098115.1 |
| 1748 | | T-complex protein 1 subunit delta-like protein | | tr|A6MKY3|A6MKY3_CALJA |
| 1749 | | Temporarily Assigned Gene name family member (tag-58) | | XP_001099479.1 |
| 1750 | | tenascin C (hexabrachion) | | XP_001099317.1 |
| 1751 | | tenascin R (restrictin, janusin) | | XP_001104570.1 |
| 1752 | | TERF1 (TRF1)-interacting nuclear factor 2 | | XP_001113445.1 |
| 1753 | | testis expressed sequence 11 isoform 1 | | XP_001086111.1 |
| 1754 | | testis expressed sequence 13A | | XP_001088131.1 |
| 1755 | | testis expressed sequence 15 | | XP_001084805.1 |
| 1756 | | testis expressed sequence 2 isoform 4 | | XP_001116528.1 |
| 1757 | | tetratricopeptide repeat domain 17 isoform 5 | | XP_001114312.1 |
| 1758 | | TGF-beta induced apoptosis protein 12 | | XP_001086838.1 |
| 1759 | | TGN47 protein | | tr|Q95193|Q95193_MACFA |
| 1760 | | thioredoxin | | XP_001113950.1 |
| 1761 | | thioredoxin-like 1 isoform 2 | | XP_001083710.1 |
| 1762 | | threonyl-tRNA synthetase, partial | | XP_001118636.1 |
| 1763 | | thrombospondin 1 precursor | | XP_001093770.1 |
| 1764 | | thrombospondin 3 isoform 8 | | XP_001115770.1 |
| 1765 | | Thrombospondin 4 | | tr|Q6UIM3|Q6UIM3_MACMU |
| 1766 | | thrombospondin 4 precursor | | XP_001109898.1 |
| 1767 | | THUMP domain containing 2 | | XP_001110330.1 |
| 1768 | | Thymidylate synthase | | tr|Q77V82|Q77V82_9GAMA |
| 1769 | | thyrotrophic embryonic factor | | XP_001104007.1 |
| 1770 | | tigger transposable element derived 5 | | XP_001097689.1 |
| 1771 | | TIP120 protein | | XP_001106780.1 |
| 1772 | | Tissue alpha-L-fucosidase precursor (Alpha-L-fucosidase I) (Alpha-L-fucoside fucohydrolase) | | XP_001095277.1 |
| 1773 | | tissue specific transplantation antigen P35B | | XP_001086298.1 |
| 1774 | | TNF receptor-associated factor 3 interacting protein 1 | | XP_001087072.1 |
| 1775 | | TNF receptor-associated factor 6 | | tr|B6CJY4|B6CJY4_CERTO |
| 1776 | | TNF receptor-associated protein 1 | | XP_001094589.1 |
| 1777 | | toll interacting protein | | XP_001090075.1 |
| 1778 | | toll-like receptor 1 | | XP_001088852.1 |
| 1779 | | Toll-like receptor 2 | | tr|B6CJY8|B6CJY8_CERTO |
| 1780 | | Toll-like receptor 3 | | tr|B6CJZ1|B6CJZ1_CERTO |
| 1781 | | Toll-like receptor 4 (Fragment) | | tr|Q3BBY2|Q3BBY2_MACMU |
| 1782 | | Toll-like receptor 4 | | tr|B6CJZ3|B6CJZ3_CERTO |
| 1783 | | Toll-like receptor 5 | | tr|B6CJZ4|B6CJZ4_CERTO |
| 1784 | | Toll-like receptor 6 | | tr|B6CJZ6|B6CJZ6_CERTO |
| 1785 | | Toll-like receptor 7 | | tr|B6CJZ8|B6CJZ8_CERTO |
| 1786 | | Toll-like receptor 8 (Fragment) | | tr|B6CK01|B6CK01_CERTO |
| 1787 | | Toll-like receptor 9 | | tr|B6CK02|B6CK02_CERTO |
| 1788 | | TPR domain, ankyrin-repeat and coiled-coil-containing isoform 2 | | XP_001093218.1 |
| 1789 | | TRAF-binding protein | | XP_001086109.1 |
| 1790 | | transaldolase 1 | | XP_001116705.1 |
| 1791 | | transcription elongation factor A (SII)-like 4 | | XP_001085077.1 |
| 1792 | | transcription elongation factor B (SIII), polypeptide 1 | | XP_001087401.1 |
| 1793 | | transcription factor B1, mitochondrial isoform 2 | | XP_001094657.1 |
| 1794 | | Transcription factor COE2 (Early B-cell factor 2) (EBF-2) (Olf-1/EBF-like 3) (OE-3) (O/E-3) isoform 4 | | XP_001108135.1 |
| 1795 | | Transcription factor E2F3 (E2F-3) | | XP_001103396.1 |
| 1796 | | transcription factor ELYS | | XP_001088135.1 |
| 1797 | | transcription factor-like nuclear regulator | | XP_001093653.1 |
| 1798 | | transcription termination factor-like protein | | XP_001100560.1 |
| 1799 | | transferrin receptor isoform 3 | | XP_001101412.1 |
| 1800 | | transformation/transcription domain-associated protein isoform 2 | | XP_001093709.1 |
| 1801 | | transforming growth factor, beta-induced, 68kDa isoform 6 | | XP_001111447.1 |
| 1802 | | transglutaminase 5 | | XP_001101645.1 |
| 1803 | | transient receptor potential cation channel, subfamily M, member 3 | | XP_001091211.1 |
| 1804 | | transient receptor potential cation channel, subfamily V, member 3 | | XP_001117602.1 |
| 1805 | | translin | | XP_001085908.1 |
| 1806 | | translin-associated factor X (Tsnax) interacting protein 1 isoform 5 | | XP_001094769.1 |
| 1807 | | translin-associated factor X isoform 2 | | XP_001104647.1 |
| 1808 | | Translocase of inner mitochondrial membrane 17 A-like protein (Fragment) | | tr|Q3YAS4|Q3YAS4_MACMU |
| 1809 | | translocase of inner mitochondrial membrane 44 homolog | | XP_001098363.1 |
| 1810 | | translokin isoform 4 | | XP_001093565.1 |
| 1811 | | transmembrane protease, serine 2 isoform 1 | | XP_001107212.1 |
| 1812 | | transmembrane protein with EGF-like and two follistatin-like domains 1 isoform 3 | | XP_001111631.1 |
| 1813 | | transportin 1 | | XP_001095625.1 |
| 1814 | | transportin 2 (importin 3, karyopherin beta 2b) | | XP_001109480.1 |
| 1815 | | TRIM22 | | tr|B0F4M6|B0F4M6_CERTO |
| 1816 | | Triosephosphate isomerase | | tr|A6MLJ4|A6MLJ4_CALJA |
| 1817 | | triosephosphate isomerase 1 | | XP_001110758.1 |
| 1818 | | tripartite motif-containing 25 | | XP_001101634.1 |
| 1819 | | tripartite motif-containing 28 protein isoform 4 | | XP_001098702.1 |
| 1820 | | tripartite motif-containing 45 isoform 3 | | XP_001113153.1 |
| 1821 | | Tripartite motif-containing 5 alpha isoform (Tripartite motif-containing 5 alpha) | | tr|A1E969|A1E969_CERTO |
| 1822 | | tripeptidyl-peptidase I precursor | | XP_001108748.1 |
| 1823 | | tropomodulin 1 isoform 4 | | XP_001114190.1 |
| 1824 | | tropomodulin 2 (neuronal) isoform 3 | | XP_001084313.1 |
| 1825 | | Tropomyosin 1 alpha chain (Alpha-tropomyosin) | | XP_001103963.1 |
| 1826 | | tropomyosin 1, alpha isoform i isoform 7 | | XP_001113000.1 |
| 1827 | | tropomyosin 2 (beta) isoform 11 | | XP_001088158.1 |
| 1828 | | tropomyosin 3 isoform 2 isoform 13 | | XP_001113426.1 |
| 1829 | | tropomyosin 3, gamma isoform 16 | | XP_001113508.1 |
| 1830 | | tropomyosin 4 isoform 2 | | XP_001092183.1 |
| 1831 | | tryptophanyl-tRNA synthetase isoform 8 | | XP_001105653.1 |
| 1832 | | TSC22 domain family, member 3 isoform 4 | | XP_001096019.1 |
| 1833 | | tuberous sclerosis 2 isoform 4 | | XP_001083508.1 |
| 1834 | | tubulin | | tr|Q9UQM3|Q9UQM3_HUMAN |
| 1835 | | Tubulin alpha-3 chain (Alpha-tubulin 3) | | XP_001110230.1 |
| 1836 | | tubulin tyrosine ligase-like family, member 4 isoform 3 | | XP_001094742.1 |
| 1837 | | tubulin, alpha 1 isoform 3 | | XP_001108924.1 |
| 1838 | | tubulin, alpha 4 isoform 8 | | XP_001101025.1 |
| 1839 | | tubulin, alpha, ubiquitous isoform 19 | | XP_001108104.1 |
| 1840 | | tubulin, alpha, ubiquitous isoform 21 | | XP_001108255.1 |
| 1841 | | tubulin, alpha-like 3 | | XP_001118645.1 |
| 1842 | | tubulin, beta 2 | | XP_001119010.1 |
| 1843 | | tubulin, beta 4 | | XP_001091329.1 |
| 1844 | | tubulin, beta 8 isoform 2 | | XP_001104203.1 |
| 1845 | | tubulin, beta isoform 3 | | XP_001092690.1 |
| 1846 | | tudor domain containing 6 isoform 2 | | XP_001103211.1 |
| 1847 | | tudor domain containing 7 | | XP_001114256.1 |
| 1848 | | tumor necrosis factor (ligand) superfamily, member 18 | | NP_001035281.1 |
| 1849 | | Tumor necrosis factor receptor superfamily member 6 | | sp|Q9BDN4|TNR6_CERTO |
| 1850 | | tumor necrosis factor receptor superfamily, member 17 isoform 1 | | XP_001106826.1 |
| 1851 | | tumor protein p53 binding protein, 2 | | XP_001093747.1 |
| 1852 | | tumor protein, translationally-controlled 1 | | XP_001112392.1 |
| 1853 | | tumor rejection antigen (gp96) 1 | | XP_001095189.1 |
| 1854 | | TXK tyrosine kinase | | XP_001103052.1 |
| 1855 | | type I hair keratin KA36 | | XP_001102221.1 |
| 1856 | | Type-1 angiotensin II receptor-associated protein (AT1 receptor-associated protein) | | XP_001118851.1 |
| 1857 | | tyrosine 3/tryptophan 5 -monooxygenase activation protein, zeta polypeptide | | XP_001098275.1 |
| 1858 | | tyrosine 3-monooxygenase/tryptophan 5-monooxygenase activation protein, beta polypeptide | | XP_001109676.1 |
| 1859 | | tyrosine 3-monooxygenase/tryptophan 5-monooxygenase activation protein, epsilon polypeptide | | XP_001117283.1 |
| 1860 | | tyrosine 3-monooxygenase/tryptophan 5-monooxygenase activation protein, eta polypeptide isoform 1 | | XP_001111955.1 |
| 1861 | | tyrosine 3-monooxygenase/tryptophan 5-monooxygenase activation protein, theta polypeptide | | XP_001097635.1 |
| 1862 | | tyrosine kinase with immunoglobulin-like and EGF-like domains 1 | | XP_001090483.1 |
| 1863 | | Tyrosine protein phosphatase non-receptor type 6-like protein | | tr|A6MKG8|A6MKG8_CALJA |
| 1864 | | tyrosyl-tRNA synthetase 2 (mitochondrial), partial | | XP_001107606.1 |
| 1865 | | U1 small nuclear ribonucleoprotein 70 kDa | | XP_001112732.1 |
| 1866 | | U2 (RNU2) small nuclear RNA auxiliary factor 2 isoform b, partial | | XP_001119590.1 |
| 1867 | | U5 snRNP-specific protein, 116 kD | | XP_001114964.1 |
| 1868 | | U5 snRNP-specific protein | | XP_001117328.1 |
| 1869 | | ubiquitin A-52 residue ribosomal protein fusion product 1 | | XP_001092853.1 |
| 1870 | | Ubiquitin activating enzyme E1-like protein (Fragment) | | tr|A6MLD4|A6MLD4_CALJA |
| 1871 | | Ubiquitin carboxyl-terminal hydrolase isozyme L1 (UCH-L1) (Ubiquitin thioesterase L1) (Neuron cytoplasmic protein 9.5) (PGP 9.5) (PGP9.5) | | XP_001097839.1 |
| 1872 | | ubiquitin specific peptidase 5 (isopeptidase T) isoform 3 | | XP_001110679.1 |
| 1873 | | ubiquitin specific protease 15 isoform 3 | | XP_001116743.1 |
| 1874 | | ubiquitin specific protease 16 isoform a isoform 4 | | XP_001102423.1 |
| 1875 | | ubiquitin specific protease 28 | | XP_001085291.1 |
| 1876 | | Ubiquitin specific protease 3 (Fragment) | | tr|Q3YAP1|Q3YAP1_MACMU |
| 1877 | | ubiquitin specific protease 44 isoform 3 | | XP_001107190.1 |
| 1878 | | ubiquitin-activating enzyme E1 isoform 4 | | XP_001092484.1 |
| 1879 | | UDP glycosyltransferase 1 family, polypeptide A1 | | NP_001028041.1 |
| 1880 | | UDP-Gal betaGlcNAc beta 1,4- galactosyltransferase 1, membrane-bound form isoform 3 | | XP_001100938.1 |
| 1881 | | UDP-Gal betaGlcNAc beta 1,4- galactosyltransferase 4 isoform 1 | | XP_001108377.1 |
| 1882 | | UDP-Gal betaGlcNAc beta 1,4- galactosyltransferase 5 | | XP_001103384.1 |
| 1883 | | UDP-galactose-4-epimerase isoform 3 | | XP_001104169.1 |
| 1884 | | UDP-GlcNAc betaGal beta-1,3-N-acetylglucosaminyltransferase 4 isoform 2 | | XP_001097591.1 |
| 1885 | | UDP-glucose dehydrogenase isoform 5 | | XP_001092696.1 |
| 1886 | | UDP-N-acetyl-alpha-D-galactosamine polypeptide N-acetylgalactosaminyltransferase 12 (GalNAc-T12) | | XP_001112919.1 |
| 1887 | | UDP-N-acetyl-alpha-D-galactosamine polypeptide N-acetylgalactosaminyltransferase 5 isoform 2 | | XP_001087663.1 |
| 1888 | | UDP-N-acetylglucosamine-2-epimerase/N-acetylmannosamine kinase | | XP_001082113.1 |
| 1889 | | UDP-N-acteylglucosamine pyrophosphorylase 1 isoform 3 | | XP_001118204.1 |
| 1890 | | UL16 binding protein 3, partial | | XP_001082838.1 |
| 1891 | | UL16 protein OS=Cercopithecine herpesvirus 1 | | tr|Q806B3|Q806B3_CHV1 |
| 1892 | | UNC-112 related protein 2 short form | | XP_001118313.1 |
| 1893 | | uncharacterized hematopoietic stem/progenitor cells protein MDS032 isoform 4 | | XP_001113229.1 |
| 1894 | | Uncharacterized metal-binding lipoprotein CT_067 | | sp|Q9S529|Y067_CHLTR |
| 1895 | | UNCoordinated family member (unc-44) | | XP_001101829.1 |
| 1896 | | uridine phosphorylase 1 | | XP_001084775.1 |
| 1897 | | urocanase domain containing 1 | | XP_001114316.1 |
| 1898 | | Uteroglobin precursor (Secretoglobin family 1A member 1) (UP1) | | XP_001116505.1 |
| 1899 | | uveal autoantigen with coiled-coil domains and ankyrin repeats isoform 1 isoform 2 | | XP_001088091.1 |
| 1900 | | V-1 reverse transcriptase (Fragment) | | tr|Q9ITU3|Q9ITU3_9HIV1 |
| 1901 | | vacuolar H+ ATPase C2 | | XP_001095057.1 |
| 1902 | | vacuolar protein sorting 26 | | XP_001083367.1 |
| 1903 | | Vacuolar protein sorting 29 (Vesicle protein sorting 29) isoform 1 | | XP_001107745.1 |
| 1904 | | vacuolar protein sorting 35 | | XP_001108828.1 |
| 1905 | | vacuolar protein sorting 39 (yeast) isoform 2 | | XP_001103143.1 |
| 1906 | | valyl-tRNA synthetase | | XP_001105797.1 |
| 1907 | | Vascular cell adhesion molecule-1 (Fragment) | | tr|Q58T13|Q58T13_MACFA |
| 1908 | | vascular endothelial growth factor C | | XP_001090231.1 |
| 1909 | | Vasodilator-stimulated phosphoprotein (VASP) | | XP_001106536.1 |
| 1910 | | vav 2 oncogene isoform 3 | | XP_001099413.1 |
| 1911 | | vesicle amine transport protein 1 | | XP_001113014.1 |
| 1912 | | vesicle docking protein p115 isoform 2 | | XP_001100598.1 |
| 1913 | | vesicle transport-related protein isoform a | | XP_001107671.1 |
| 1914 | | Vif protein (Fragment) | | tr|B2C4P6|B2C4P6_9HIV1 |
| 1915 | | villin 2 | | XP_001093089.1 |
| 1916 | | vimentin | | XP_001093658.1 |
| 1917 | | virilizer CG3496-PA isoform 3 | | XP_001089437.1 |
| 1918 | | Virion infectivity factor | | tr|Q73446|Q73446_9HIV1 |
| 1919 | | vitamin D-binding protein | | XP_001104833.1 |
| 1920 | | Vitamin K-dependent protein S | | sp|P07225|PROS_HUMAN |
| 1921 | | vitamin K-dependent protein S precursor | | NP_001038191.1 |
| 1922 | | vitrin isoform 3 | | XP_001107747.1 |
| 1923 | | vitronectin | | XP_001106884.1 |
| 1924 | | v-Ki-ras2 Kirsten rat sarcoma viral oncogene homolog | | XP_001101840.1 |
| 1925 | | voltage gated channel like 1 | | XP_001095394.1 |
| 1926 | | von Willebrand factor A domain containing 2 isoform 3 | | XP_001091779.1 |
| 1927 | | von Willebrand factor type A and cache domain containing 1 | | XP_001089910.1 |
| 1928 | | Vpr | | sp|P19509|VPR_SIVSP |
| 1929 | | vpr, Truncated | | tr|Q3S5K6|Q3S5K6_9HIV1 |
| 1930 | | VPS10 domain receptor protein SORCS 3 | | XP_001082205.1 |
| 1931 | | Vpu protein | | tr|Q202K8|Q202K8_9HIV1 |
| 1932 | | Vpx | | tr|O90319|O90319_SIVCZ |
| 1933 | | v-ral simian leukemia viral oncogene homolog A (ras related) isoform 2 | | XP_001099021.1 |
| 1934 | | WD repeat domain 44 protein isoform 3 | | XP_001104919.1 |
| 1935 | | WD repeat domain 54 isoform 3 | | XP_001108927.1 |
| 1936 | | WD repeat domain 58 isoform 3 | | XP_001090442.1 |
| 1937 | | WD repeat domain 61 | | XP_001107597.1 |
| 1938 | | WD repeat protein 61-like protein (Fragment) | | tr|A6MKY0|A6MKY0_CALJA |
| 1939 | | WD repeat-containing protein 1 isoform 6 | | XP_001097937.1 |
| 1940 | | wingless-type MMTV integration site family, member 6 | | XP_001095541.1 |
| 1941 | | Wins2 protein | | XP_001083049.1 |
| 1942 | | WW domain binding protein 2 isoform 3 | | XP_001100942.1 |
| 1943 | | xenotropic and polytropic retrovirus receptor isoform 2 | | XP_001115004.1 |
| 1944 | | X-prolyl aminopeptidase (aminopeptidase P) 1, soluble | | XP_001085192.1 |
| 1945 | | xylulokinase homolog isoform 2 | | XP_001086867.1 |
| 1946 | | Y54G11A.7 | | XP_001110757.1 |
| 1947 | | YIPF3-like protein (Fragment) | | tr|A6MKB6|A6MKB6_CALJA |
| 1948 | | zinc binding alcohol dehydrogenase, domain containing 1 | | XP_001091894.1 |
| 1949 | | zinc finger and BTB domain containing 40 isoform 4 | | XP_001101372.1 |
| 1950 | | zinc finger and BTB domain containing 9 | | XP_001116313.1 |
| 1951 | | zinc finger CCCH type containing 12A | | XP_001104883.1 |
| 1952 | | zinc finger protein 142 | | XP_001090487.1 |
| 1953 | | zinc finger protein 161 homolog | | XP_001082120.1 |
| 1954 | | zinc finger protein 222 isoform 2 | | XP_001108117.1 |
| 1955 | | zinc finger protein 224 | | XP_001102371.1 |
| 1956 | | zinc finger protein 225 isoform 3 | | XP_001108548.1 |
| 1957 | | zinc finger protein 25 isoform 2 | | XP_001096234.1 |
| 1958 | | zinc finger protein 261 isoform 6 | | XP_001090142.1 |
| 1959 | | zinc finger protein 285 isoform 1 | | XP_001108851.1 |
| 1960 | | zinc finger protein 292 | | XP_001088554.1 |
| 1961 | | zinc finger protein 3 isoform 2 | | XP_001105241.1 |
| 1962 | | zinc finger protein 306 isoform 2 | | XP_001100771.1 |
| 1963 | | zinc finger protein 365 isoform A isoform 2 | | XP_001093064.1 |
| 1964 | | zinc finger protein 383 | | XP_001113276.1 |
| 1965 | | zinc finger protein 415 isoform 3 | | XP_001116739.1 |
| 1966 | | Zinc finger protein 479 (Zinc finger protein Kr19) (HKr19) | | XP_001094948.1 |
| 1967 | | zinc finger protein 500 | | XP_001098209.1 |
| 1968 | | zinc finger protein 503 isoform 4 | | XP_001095797.1 |
| 1969 | | zinc finger protein 528 | | XP_001115320.1 |
| 1970 | | zinc finger protein 533 | | XP_001108755.1 |
| 1971 | | zinc finger protein 564 | | XP_001109129.1 |
| 1972 | | zinc finger protein 629 | | XP_001102645.1 |
| 1973 | | zinc finger protein 652 | | XP_001092787.1 |
| 1974 | | zinc finger protein 696 | | XP_001096275.1 |
| 1975 | | zinc finger protein 8, partial | | XP_001118943.1 |
| 1976 | | zinc finger protein ZFP isoform 1 | | XP_001105795.1 |
| 1977 | | zinc finger, CCHC domain containing 11 isoform b isoform 5 | | XP_001111993.1 |
| 1978 | | zinc finger, ZZ type with EF hand domain 1 | | XP_001117691.1 |
| 1979 | | Zinc phosphodiesterase ELAC protein 2 | | sp|Q8CGS5|RNZ2_RAT |
