# Supplementary material for: Distinct host cell proteins incorporated by SIV replicating in CD4+ T Cells from natural disease resistant versus non-natural disease susceptible hosts
Source: Retrovirology. 2010 Dec 16;7:107. doi: 10.1186/1742-4690-7-107 (PMC3012658; doi:10.1186/1742-4690-7-107)
Supplement: Additional file 2 — A list of host proteins common to virus preparations from rhesus macaques and sooty mangabeys. A list of 202 host proteins that were found in virus preparations of CD4+ T cells from both rhesus macaques and sooty mangabeys. [file 1742-4690-7-107-S2.DOC]

**Additional 2**

| **Host proteins found in all SIV samples analyzed** | **Reference**  **Number** | **Category** |
| --- | --- | --- |
| 14-3-3 protein beta/alpha | sp|A4K2U9|1433B_PONAB | immune function associated |
| 60S acidic ribosomal protein P0 (L10E), partial | XP_001115939.1 | Ribosome |
| 60S acidic ribosomal protein P2 (NY-REN-44 antigen) | XP_001094442.1 | Ribosome |
| 60S ribosomal protein L14 (CAG-ISL 7), partial | XP_001115863.1 | Ribosome |
| 60S ribosomal protein L7a | XP_001118392.1 | Ribosome |
| Actin beta subunit | Q6UIS2_PANTR | membrane / cytoskeleton |
| Actin related protein 2/3 complex subunit 2 | XP_001088889.1 | membrane / cytoskeleton |
| Actin, alpha cardiac (Alpha-cardiac actin) isoform 3 | XP_001088409.1 | membrane / cytoskeleton |
| Actin, cytoplasmic 2 (Gamma-actin) isoform 1 | XP_001110688.1 | membrane / cytoskeleton |
| Actinin alpha 4 isoform 5 | XP_001083825.1 | membrane / cytoskeleton |
| Adenine phosphoribosyltransferase isoform 2 | XP_001089867.1 | metabolic |
| Adenylyl cyclase-associated protein | XP_001082428.1 | membrane / cytoskeleton |
| ADP-ribosylation factor 3 | XP_001104802.1 | multiple biological function (other) |
| Albumin | XP_001103956.1 | Serum protein |
| Alpha 2 globin | XP_001094404.1 | hematological |
| Alpha-2-HS-glycoprotein isoform 2 | XP_001091623.1 | membrane / cytoskeleton |
| Alpha-2-macroglobulin isoform 3 | XP_001114328.1 | hematological |
| Annexin A11 | XP_001096044.1 | membrane / cytoskeleton |
| Annexin VI | XP_001100437.1 | membrane / cytoskeleton |
| Apolipoprotein A-I precursor (Apo-AI)(ApoA-I) | XP_001090535.1 | metabolic |
| Apolipoprotein B | Q866M8_CALMO | metabolic |
| Apolipoprotein B isoform 2 | XP_001097500.1 | metabolic |
| Apolipoprotein E | XP_001104482.1 | metabolic |
| Aspartyl-tRNA synthetase | XP_001095858.1 | metabolic |
| ATP-dependent DNA helicase II, 70 kDa subunit | XP_001105684.1 | metabolic |
| ATP-dependent DNA helicase II | XP_001084586.1 | metabolic |
| Beta globin | XP_001111709.1 | hematological |
| Biglycan | XP_001086112.1 | extracellular matrix |
| Bromodomain containing 9 isoform 2 isoform 4 | XP_001096110.1 | nuclear protein |
| C1q and tumor necrosis factor related protein 3 isoform b | XP_001090621.1 | immune function associated |
| Calreticulin isoform 2 | XP_001110217.1 | immune function associated |
| Chondroitin 6 sulfotransferase 3 | XP_001106464.1 | extracellular matrix |
| Cartilage oligomeric matrix protein precursor | XP_001115565.1 | extracellular matrix |
| CD109 isoform 2 | XP_001112930.1 | immune function associated |
| CD44 molecule (Indian blood group)isoform 10 | XP_001115390.1 | immune function associated |
| cDNA sequence BC048546 | XP_001118458.1 | ? (other) |
| chaperonin containing TCP1, subunit 2 | XP_001108460.1 | protein folding |
| Chaperonin containing TCP1, subunit 3 isoform a isoform 4 | XP_001116562.1 | protein folding |
| Chaperonin containing TCP1, subunit 4 (delta) | XP_001114200.1 | protein folding |
| Chaperonin containing TCP1, subunit 5 (epsilon) isoform 3 | XP_001085610.1 | protein folding |
| Chaperonin containing TCP1, subunit 6A isoform a isoform 3 | XP_001090218.1 | protein folding |
| Chaperonin containing TCP1, subunit 8 (theta) isoform 5 | XP_001101969.1 | protein folding |
| Clathrin heavy chain 1 isoform 5 | XP_001108702.1 | Membrane/  cytoskeleton |
| Coagulation factor II | XP_001101815.1 | coagulation |
| Coagulation factor V precursor | XP_001093072.1 | coagulation |
| Coagulation factor X | NP_001098054.1 | coagulation |
| Coagulation factor XIII A1 subunit isoform 2 | XP_001096779.1 | coagulation |
| Cofilin-1 (Cofilin, non-muscle isoform) (18 kDa phosphoprotein) (p18) | XP_001118077.1 | membrane / cytoskeleton |
| Collagen alpha-1(VI) chain precursor | XP_001118050.1 | extracellular matrix |
| Complement component 3, partial | XP_001091921.1 | immune function associated |
| Complement component 4A preproprotein, partial | XP_001117272.1 | immune function associated |
| Complement component 4B preproprotein, partial | XP_001119226.1 | immune function associated |
| Complement component 9 | XP_001084671.1 | immune function associated |
| Coronin, actin binding protein, 1A | XP_001099485.1 | membrane / cytoskeleton |
| C-type lectin domain family 11, member A isoform 3 | XP_001116136.1 | membrane / cytoskeleton |
| Actin - beta isoform 2 cytoplasmic | XP_001110809.1 | membrane / cytoskeleton |
| Dermcidin preproprotein | XP_001092492.1 | extracellular matrix |
| Desmoplakin isoform I isoform 2 | XP_001085012.1 | membrane / cytoskeleton |
| Elongin A isoform 1 | XP_001103322.1 | nuclear protein |
| Enolase 1 isoform 8 | XP_001098883.1 | metabolic |
| Envelope glycoprotein | Q6J3P9_SIVCZ | viral protein |
| Envelope glycoprotein gp120 | Q72951_9HIV1 | viral protein |
| Envelope glycoprotein gp160 | B3GV32_9HIV1 | viral protein |
| Eukaryotic translation elongation factor 1 | Q3YAP9_MACMU | ribosome |
| Eukaryotic translation elongation factor 2 | XP_001118006.1 | ribosome |
| Eukaryotic translation initiation factor 3 subunit 6 interacting protein isoform 2 | XP_001090065.1 | ribosome |
| Eukaryotic translation initiation factor 3, subunit 10 theta, 150/170kDa | XP_001102472.1 | ribosome |
| Eukaryotic translation initiation factor 3, subunit 5 epsilon, 47kDa isoform 3 | XP_001105893.1 | ribosome |
| Eukaryotic translation initiation factor 3, subunit 9 eta, 116kDa | XP_001086876.1 | ribosome |
| Eukaryotic translation initiation factor 4A, isoform 1 isoform 3 | XP_001109752.1 | ribosome |
| F-actin capping protein alpha-1 subunit | XP_001107677.1 | membrane / cytoskeleton |
| Fetuin B isoform 3 | XP_001091143.1 | membrane / cytoskeleton |
| Fibromodulin precursor | XP_001102658.1 | extracellular matrix |
| Fibronectin 1 isoform 1 preproprotein | XP_001083548.1 | extracellular matrix |
| Fibulin 1 isoform 2 | XP_001109966.1 | extracellular matrix |
| Filaggrin 2 | XP_001109011.1 | membrane / cytoskeleton |
| Filamin 1 (actin-binding protein-280) isoform 5 | XP_001091203.1 | membrane / cytoskeleton |
| Gag polyprotein | Q5IK81_9HIV1 | viral protein |
| Gag-Pol polyprotein | sp|P17283|POL_SIVCZ | viral protein |
| GAPDH (Fragment) | Q6IUG3_MACMU | metabolic |
| GAPDH | XP_001105471.1 | metabolic |
| Gelsolin isoform 19 | XP_001093567.1 | membrane / cytoskeleton |
| Glutathione S-transferase P | NP_001036141.1 | metabolic |
| Guanine nucleotide-binding protein beta subunit 2-like 1 (RACK1) isoform 4 | XP_001105066.1 | intracellular signaling |
| Haptoglobin | Q5VAN2_CERTO | hematological |
| Heat shock 70kDa protein 1-like isoform 2 | XP_001113329.1 | protein folding |
| Heat shock protein 90 protein 1 beta | Q3YAN8_MACMU | protein folding |
| Heat shock protein 90 protein 1 alpha | Q3YAP6_MACMU | protein folding |
| Heat shock protein 90 protein 1beta isoform 10 | XP_001099439.1 | protein folding |
| Hemoglobin | sp|P01933|HBA_CERTO | metabolic |
| Heparan sulfate proteoglycan 2 | XP_001099299.1 | membrane / cytoskeleton |
| Heterogeneous nuclear ribonucleoprotein K isoform a isoform 13 | XP_001105897.1 | nuclear protein |
| Histone family H2A, member Z isoform 2 | XP_001108128.1 | nuclear protein |
| Histone H3, family 3B | XP_001104869.1 | nuclear protein |
| Hyaluronan binding protein 2 | XP_001090138.1 | immune function associated |
| Hypothetical protein isoform 3 | XP_001091507.1 | ? (other) |
| Hypothetical protein | XP_001107765.1 | ? (other) |
| Integrin alpha L | XP_001100800.1 | immune function associated |
| Inter-alpha (globulin) inhibitor H1 | XP_001084712.1 | trypsin inhibitor |
| Inter-alpha (globulin) inhibitor H3 | XP_001085463.1 | trypsin inhibitor |
| Inter-alpha globulin inhibitor H2 polypeptide | XP_001107718.1 | trypsin inhibitor |
| Interferon induced transmembrane protein 1 (9-27) | XP_001085444.1 | Intracellular signaling |
| Interleukin-12 | sp|P46661|IL12A_CERTO | immune function associated |
| Junction plakoglobin | XP_001107394.1 | membrane / cytoskeleton |
| Karyopherin beta 1 | XP_001082833.1 | intracellular trafficking |
| Katanin p60 subunit A 1 | XP_001085922.1 | nuclear protein |
| Kininogen 1 isoform 4 | XP_001102055.1 | coagulation |
| Lactate dehydrogenase A | XP_001086967.1 | metabolic |
| Lactate dehydrogenase B | XP_001117178.1 | metabolic |
| Laminin, beta 1 | XP_001090393.1 | membrane / cytoskeleton |
| Leucine aminopeptidase isoform 2 | XP_001102787.1 | metabolic |
| L-lactate dehydrogenase B chain-like protein | A6MK87_CALJA | metabolic |
| Low density lipoprotein-related protein 1, partial | XP_001099678.1 | extracellular matrix |
| Lumican isoform 2 | XP_001102753.1 | extracellular matrix |
| Lysozyme C | sp|P61630|LYSC_CERTO | enzyme |
| MHC class I antigen | A5YWB6_MACFA | immune function associated |
| MHC class II antigen | Q9TP91_9PRIM | immune function associated |
| Mitogen-activated protein kinase kinase 3 isoform B isoform 3 | XP_001104193.1 | intracellular signaling |
| Myosin regulatory light chain 2, nonsarcomeric (Myosin RLC) isoform 2 | XP_001084519.1 | membrane / cytoskeleton |
| Myosin, heavy polypeptide 9, non-muscle | XP_001083662.1 | membrane / cytoskeleton |
| Myosin, light polypeptide 6, alkali, smooth muscle and non-muscle isoform 7 | XP_001114459.1 | membrane / cytoskeleton |
| Myosin-9 (Myosin heavy chain, nonmuscle IIa), partial | XP_001116722.1 | membrane / cytoskeleton |
| Nef protein | A0EUQ8_9HIV1 | viral protein |
| Neuropilin 1 isoform 13 | XP_001087374.1 | membrane / cytoskeleton |
| Neuropilin 2 isoform 6 precursor | XP_001104807.1 | membrane / cytoskeleton |
| Nucleophosmin 1 isoform 2 | XP_001095288.1 | ribosome |
| Nucleosome assembly protein 1-like1isoform 8 | XP_001117640.1 | nuclear protein |
| Pentraxin-related gene, rapidly induced by IL-1 beta | XP_001103515.1 | immune function associated |
| Phosphoglycerate kinase 1 | Q3YAQ9_MACMU | metabolic |
| Phosphoglycerate kinase 1 isoform 4 | XP_001100787.1 | metabolic |
| Pol polypeptide | Q5TYK3_SIVCZ | viral protein |
| Protease | A8JSS8_9HIV1 | viral protein |
| Protease, serine, 1 (trypsin 1) | XP_001088295.1 | putative trypsin-6 |
| 26S proteasome (prosome, macropain) subunit, non-ATPase, 14 | XP_001096177.1 | proteasome |
| Proteasome (prosome, macropain) subunit alpha, type 7 isoform 1, partial | XP_001114742.1 | proteasome |
| Proteasome (prosome, macropain) subunit, beta, type 1 isoform 2 | XP_001084784.1 | proteasome |
| Proteasome (prosome, macropain) subunit, beta, type 3 isoform 2 | XP_001084269.1 | proteasome |
| Proteasome (prosome, macropain) subunit, beta, type 6 isoform 2 | XP_001096870.1 | proteasome |
| Proteasome (prosome, macropain) subunit beta, type 2 isoform 2 | XP_001109596.1 | proteasome |
| 26S proteasome non-ATPase regulatory subunit 11 | XP_001110049.1 | proteasome |
| 26S proteasome non-ATPase regulatory subunit 2 | XP_001093407.1 | proteasome |
| Proteasome subunit beta type 4 like isoform 3 | XP_001108305.1 | proteasome |
| Proteasome subunit beta type 5 | XP_001105158.1 | proteasome |
| Proteasome subunit alpha type 1 (Proteasome component C2) (PROS-30) | XP_001089332.1 | proteasome |
| Proteasome subunit alpha type 1-like protein (Fragment) | A6MJW2_CALJA | proteasome |
| Proteasome subunit alpha type 4 (Proteasome component C9) isoform 3 | XP_001108132.1 | proteasome |
| Proteasome subunit alpha type 5 (Proteasome zeta chain) | XP_001090512.1 | proteasome |
| Proteasome subunit alpha type 6 (Proteasome beta chain) isoform 1 | XP_001095366.1 | proteasome |
| Protein SET (Phosphatase 2A inhibitor I2PP2A) (I-2PP2A) isoform 2 | XP_001110579.1 | intracellular signaling |
| Protein tyrosine phosphatase, receptor-type, zeta1 | XP_001083149.1 | intracellular signaling |
| Prothrombin precursor (Coagulation factor II), partial | XP_001111947.1 | coagulation |
| PRP19/PSO4 pre-mRNA processing factor 19 homolog | XP_001084826.1 | ribosome |
| Pyruvate kinase 3 isoform 9 | XP_001091427.1 | metabolic |
| RAB11a, member RAS oncogene family | XP_001103732.1 | intracellular trafficking |
| RAB5B, member RAS oncogene family | XP_001096821.1 | intracellular signaling |
| RAB6A, member RAS oncogene family isoform a | XP_001115437.1 | intracellular signaling |
| RAN binding protein 5 isoform 4 | XP_001089501.1 | intracellular trafficking |
| RAN, member RAS oncogene family | XP_001104422.1 | intracellular signaling |
| Retinoblastoma binding protein 4 isoform 6 | XP_001104415.1 | nuclear protein |
| Rev | Q1A254_SIVCZ | viral protein |
| Reverse transcriptase | Q7SL19_9HIV1 | viral protein |
| Ribosomal protein L18 | XP_001092745.1 | ribosome |
| Ribosomal protein P1 | Q6V8L6_MACRA | ribosome |
| Ribosomal protein S18 isoform 3 | XP_001099240.1 | ribosome |
| Ribosomal protein S8 isoform 1 | XP_001098975.1 | ribosome |
| Ribosomal protein SA | Q3YAI0_MACMU | ribosome |
| S-adenosylhomocysteine hydrolase | XP_001104495.1 | metabolic |
| Septin 2 | XP_001109436.1 | membrane / cytoskeleton |
| Septin-9 (MLL septin-like fusion protein), partial | XP_001116653.1 | membrane / cytoskeleton |
| Serine (or cysteine) proteinase inhibitor, clade A, member 7 | XP_001088790.1 | metabolic |
| Serine (or cysteine) proteinase inhibitor, clade C (antithrombin), member 1 | NP_001098053.1 | metabolic |
| SET translocation | Q3YAR7_MACMU | endoplasmic reticulum (other) |
| SNF2 histone linker PHD RING helicase | XP_001086641.1 | nuclear protein |
| SPARC-like 1 isoform 3 | XP_001097743.1 | intracellular signaling |
| Stomatin isoform 1 | XP_001090536.1 | membrane / cytoskeleton |
| Talin 1 | XP_001084941.1 | membrane / cytoskeleton |
| T-complex protein 1 isoform 6 | XP_001098115.1 | intracellular trafficking |
| T-complex protein 1 subunit delta-like protein | A6MKY3_CALJA | intracellular trafficking |
| Thrombospondin 1 precursor | XP_001093770.1 | immune function associated |
| Thrombospondin 4 | Q6UIM3_MACMU | immune function associated |
| Thrombospondin 4 precursor | XP_001109898.1 | immune function associated |
| TIP120 protein | XP_001106780.1 | other |
| TNF receptor-associated factor 6 | B6CJY4_CERTO | immune function associated |
| TNF receptor-associated protein 1 | XP_001094589.1 | immune function associated |
| Toll-like receptor 1 | XP_001088852.1 | immune function associated |
| TRIM22 | B0F4M6_CERTO | Ubiquitin ligase |
| Tryptophanyl-tRNA synthetase isoform 8 | XP_001105653.1 | membrane / cytoskeleton |
| Tubulin | Q9UQM3_HUMAN | membrane / cytoskeleton |
| Tubulin, alpha 1 isoform 3 | XP_001108924.1 | membrane / cytoskeleton |
| Tubulin, beta 2 | XP_001119010.1 | membrane / cytoskeleton |
| Tubulin, beta 4 | XP_001091329.1 | membrane / cytoskeleton |
| Tyrosine 3/tryptophan 5 -monooxygenase activation protein, zeta polypeptide | XP_001098275.1 | metabolic |
| Ubiquitin A-52 residue ribosomal protein fusion product 1 | XP_001092853.1 | proteasome |
| Ubiquitin-activating enzyme E1 isoform 4 | XP_001092484.1 | proteasome |
| UNC-112 related protein 2 short form | XP_001118313.1 | proteasome |
| Vimentin | XP_001093658.1 | membrane / cytoskeleton |
| Virion infectivity factor | Q73446_9HIV1 | viral protein |
| Vitronectin | XP_001106884.1 | membrane / cytoskeleton |
| Vpu protein | Q202K8_9HIV1 | viral protein |
| WD repeat-containing protein 1 isoform 6 | XP_001097937.1 | membrane / cytoskeleton |
| Zinc finger protein 500 | XP_001098209.1 | other |
| Zinc finger, CCHC domain containing 11 isoform b isoform 5 | XP_001111993.1 | other |
|  |  |  |
