# Supplementary material for: Distinct host cell proteins incorporated by SIV replicating in CD4+ T Cells from natural disease resistant versus non-natural disease susceptible hosts
Source: Retrovirology. 2010 Dec 16;7:107. doi: 10.1186/1742-4690-7-107 (PMC3012658; doi:10.1186/1742-4690-7-107)
Supplement: Additional file 3 — A list of proteins found in common between our database and those from Chertova et al. [19]. A list of host proteins that were identified in virus preparations from rhesus macaques and sooty mangabeys and also by the studies of Chertova et al. [19]. [file 1742-4690-7-107-S3.DOC]

**Additional file 2: Proteins in Common with Chertova, E. et al J. Virol. 80:9039,2006**

|  | **Protein Name** | **Reference** |
| --- | --- | --- |
| 1 | 14-3-3 protein zeta/delta (Protein kinase C inhibitor protein 1) (KCIP-1) | XP_001111077.1 |
| 2 | 2',3'-cyclic nucleotide 3' phosphodiesterase | NP_001030605.1 |
| 3 | 40S ribosomal protein S16 | XP_001096607.1 |
| 4 | 78 kDa glucose-regulated protein precursor (GRP 78), partial | XP_001117657.1 |
| 5 | actin related protein 2/3 complex subunit 1B | XP_001111652.1 |
| 6 | actinin alpha 4 isoform 5 | XP_001083825.1 |
| 7 | adenylyl cyclase-associated protein | XP_001082428.1 |
| 8 | ADP-ribosylation factor 1 isoform 2 | XP_001106772.1 |
| 9 | annexin A11 | XP_001096044.1 |
| 10 | annexin A2 isoform 1 | XP_001094593.1 |
| 11 | apolipoprotein E | XP_001104482.1 |
| 12 | ATP synthase, H+ transporting, mitochondrial F1 complex, beta subunit | XP_001091520.1 |
| 13 | B-cell receptor-associated protein 31 | XP_001085171.1 |
| 14 | Beta 2-microglobulin | tr|Q9TS09|Q9TS09_PAPHA |
| 15 | biliverdin reductase A | XP_001095668.1 |
| 16 | catalase isoform 2 | XP_001115625.1 |
| 17 | CD14 antigen | XP_001087242.1 |
| 18 | CD2 antigen (p50), sheep red blood cell receptor | XP_001112881.1 |
| 19 | CD44 antigen | sp|P15379|CD44_MOUSE |
| 20 | CD53 antigen isoform 2 | XP_001102109.1 |
| 21 | CD81 molecule | XP_001093228.1 |
| 22 | CD82 molecule isoform 2 | XP_001113618.1 |
| 23 | CD9 molecule isoform 2 | XP_001102751.1 |
| 24 | clathrin heavy chain 1 isoform 1 | XP_001108373.1 |
| 25 | Complement C3 precursor | XP_001104310.1 |
| 26 | complement component 4B preproprotein, partial | XP_001119226.1 |
| 27 | coronin, actin binding protein, 1A | XP_001099485.1 |
| 28 | cyclophilin A | NP_001027981.1 |
| 29 | desmoplakin isoform I isoform 2 | XP_001085012.1 |
| 30 | DnaJ (Hsp40) homolog, subfamily C, member 6 isoform 4 | XP_001090170.1 |
| 31 | enolase 1 isoform 8 | XP_001098883.1 |
| 32 | eukaryotic translation elongation factor 1 alpha 1 isoform 1 | XP_001112479.1 |
| 33 | F-actin capping protein alpha-1 subunit | XP_001107677.1 |
| 34 | fibulin 1 isoform 2 | XP_001109966.1 |
| 35 | FMR1 interacting protein 1 isoform a - cytoplasmic | XP_001113740.1 |
| 36 | GDP dissociation inhibitor 2 isoform 5 | XP_001105854.1 |
| 37 | gelsolin isoform 19 | XP_001093567.1 |
| 38 | glutathione S-transferase P | NP_001036141.1 |
| 39 | glypican 4 | XP_001097252.1 |
| 40 | heat shock protein 60 (mitochondrial)-like protein | tr|A6MK13|A6MK13_CALJA |
| 41 | histone family H2B, member E | XP_001096102.1 |
| 42 | histone H3, family 3B | XP_001104869.1 |
| 43 | HLA class II histocompatibility antigen, DR alpha chain precursor (MHC class II antigen DRA) | XP_001119594.1 |
| 44 | integrin beta chain, beta 3 precursor isoform 2 | XP_001116013.1 |
| 45 | inter-alpha globulin inhibitor H2 polypeptide | XP_001107718.1 |
| 46 | junction plakoglobin | XP_001107394.1 |
| 47 | kinesin family member C3 | XP_001100393.1 |
| 48 | lectin, galactoside-binding, soluble, 1 (galectin 1) isoform 2 | XP_001083427.1 |
| 49 | L-plastin isoform 5 | XP_001098697.1 |
| 50 | Lysozyme C | sp|P61630|LYSC_CERTO |
| 51 | Macrophage capping protein-like protein | tr|A6MKP8|A6MKP8_CALJA |
| 52 | Malate dehydrogenase | tr|A6ML54|A6ML54_CALJA |
| 53 | MHC (Fragment) | tr|Q30740|Q30740_MACNE |
| 54 | moesin | XP_001100546.1 |
| 55 | N-Acetylglucosamine kinase isoform 3 | XP_001102037.1 |
| 56 | periostin, osteoblast specific factor isoform 4 | XP_001085920.1 |
| 57 | Peroxiredoxin 1 | tr|Q9BGI4|Q9BGI4_BOVIN |
| 58 | peroxiredoxin 6 | XP_001101473.1 |
| 59 | Phosphoglycerate kinase 1 | tr|Q3YAQ9|Q3YAQ9_MACMU |
| 60 | Phosphoglycerate mutase 1 | tr|Q3YAR2|Q3YAR2_MACMU |
| 61 | Pigment epithelium-derived factor precursor (PEDF) (EPC-1) | XP_001117361.1 |
| 62 | proteasome (prosome, macropain) 26S subunit, non-ATPase, 14 | XP_001096177.1 |
| 63 | proteasome (prosome, macropain) activator subunit 1 (PA28 alpha) | XP_001104073.1 |
| 64 | quiescin Q6 isoform a | XP_001111489.1 |
| 65 | rab11-family interacting protein 3 | XP_001118464.1 |
| 66 | radixin | XP_001104955.1 |
| 67 | RAP1A, member of RAS oncogene family (Fragment) | tr|Q3YAJ2|Q3YAJ2_MACMU |
| 68 | RAP1B, member of RAS oncogene family | XP_001082451.1 |
| 69 | ras-related C3 botulinum toxin substrate 2 (rho family, small GTP binding protein Rac2) | XP_001086228.1 |
| 70 | retinoic acid receptor, beta isoform 2 | XP_001092452.1 |
| 71 | Rho GTPase activating protein 1 | XP_001101907.1 |
| 72 | S100 calcium binding protein A8 (calgranulin A) isoform 2 | XP_001110492.1 |
| 73 | talin 1 | XP_001084941.1 |
| 74 | tenascin C (hexabrachion) | XP_001099317.1 |
| 75 | triosephosphate isomerase 1 | XP_001110758.1 |
| 76 | tubulin, alpha 1 isoform 3 | XP_001108924.1 |
| 77 | Tyrosine protein phosphatase non-receptor type 6-like protein | tr|A6MKG8|A6MKG8_CALJA |
| 78 | Vacuolar protein sorting 29 (Vesicle protein sorting 29) isoform 1 | XP_001107745.1 |
| 79 | vimentin | XP_001093658.1 |
