# Supplementary material for: Distinct host cell proteins incorporated by SIV replicating in CD4+ T Cells from natural disease resistant versus non-natural disease susceptible hosts
Source: Retrovirology. 2010 Dec 16;7:107. doi: 10.1186/1742-4690-7-107 (PMC3012658; doi:10.1186/1742-4690-7-107)
Supplement: Additional file 4 — A list of proteins found in common between our database and those from Brass et al. [26]. A list of host proteins that were identified in virus preparations from rhesus macaques and sooty mangabeys and also by the studies of Brass et al [26]. [file 1742-4690-7-107-S4.DOC]

**Additional file 4: Proteins in common with Brass, A. L. et al Science 319:921, 2008**

|  | **Protein** | **Reference** |
| --- | --- | --- |
| 1 | ADAM metallopeptidase domain 10 | XP_001097016.1 |
| 2 | adaptor-related protein complex 2, mu 1 subunit | XP_001102601.1 |
| 3 | ADP-ribosylation factor 1 | XP_001106772.1 |
| 4 | ATPase, H+ transporting, lysosomal V0 subunit a1 | XP_001091627.1 |
| 5 | CD4 molecule | tr|Q95NE9|Q95NE9_CERPY |
| 6 | chemokine (C-X-C motif) receptor 4 | sp|O62747|CXCR4_CERTO |
| 7 | DEAD (Asp-Glu-Ala-Asp) box polypeptide 53 | XP_001087519.1 |
| 8 | DEAD (Asp-Glu-Ala-Asp) box polypeptide 55 | XP_001098453.1 |
| 9 | epidermal growth factor receptor pathway substrate 8 | XP_001086805.1 |
| 10 | eukaryotic translation initiation factor 2C, 3 | XP_001100725.1 |
| 11 | eukaryotic translation initiation factor 3, subunit H | XP_001090244.1 |
| 12 | eukaryotic translation termination factor 1 | XP_001114680.1 |
| 13 | family with sequence similarity 76, member B | XP_001111954.1 |
| 14 | F-box protein 21 | XP_001110935.1 |
| 15 | F-box protein, helicase, 18 | XP_001107854.1 |
| 16 | flightless I homolog (Drosophila) | XP_001094751.1 |
| 17 | GABA(A) receptor-associated protein-like 2 | XP_001109173.1 |
| 18 | HECT, UBA and WWE domain containing 1 | XP_001088987.1 |
| 19 | inositol 1,4,5-trisphosphate 3-kinase A | XP_001099176.1 |
| 20 | isocitrate dehydrogenase 1 (NADP+), soluble | XP_001107627.1 |
| 21 | leucyl-tRNA synthetase | XP_001095167.1 |
| 22 | major histocompatibility complex, class I-related | NP_001073139.2 |
| 23 | neurofibromin 2 (bilateral acoustic neuroma) | XP_001106489.1 |
| 24 | N-myristoyltransferase 1 | XP_001115181.1 |
| 25 | pleckstrin homology domain containing, family A (phosphoinositide binding specific) member 3 | XP_001103375.1 |
| 26 | proteasome (prosome, macropain) activator subunit 2 (PA28 beta) | XP_001109541.1 |
| 27 | RAB1B, member RAS oncogene family | XP_001118047.1 |
| 28 | RAB28, member RAS oncogene family | XP_001096730.1 |
| 29 | RAB6A, member RAS oncogene family | XP_001115437.1 |
| 30 | Rab9 effector protein with kelch motifs | XP_001099777.1 |
| 31 | RAP1B, member of RAS oncogene family | XP_001082451.1 |
| 32 | Rho GTPase-activating protein | XP_001101907.1 |
| 33 | Rho guanine nucleotide exchange factor (GEF) 19 | XP_001092728.1 |
| 34 | septin 8 | XP_001104760.1 |
| 35 | spastin | XP_001115847.1 |
| 36 | spectrin, beta, non-erythrocytic 1 | XP_001114804.1 |
| 37 | tubulin, alpha-like 3 | XP_001108924.1 |
