# Supplementary material for: Distinct host cell proteins incorporated by SIV replicating in CD4+ T Cells from natural disease resistant versus non-natural disease susceptible hosts
Source: Retrovirology. 2010 Dec 16;7:107. doi: 10.1186/1742-4690-7-107 (PMC3012658; doi:10.1186/1742-4690-7-107)
Supplement: Additional file 5 — A list of proteins found in common between our database and those from Gautier et al.[30]. A list of host proteins that were identified in virus preparations from rhesus macaques and sooty mangabeys and also by the studies of Gautier et al [30]. [file 1742-4690-7-107-S5.DOC]

**Additional file 5: Proteins in common with Gauthier, V. W. et al Retrovirology 6:47, 2009**

|  | **Protein** | **Reference Number** |
| --- | --- | --- |
| 1 | actin-like 6A | NP_001098029.1 |
| 2 | activating signal cointegrator 1 complex subunit 3-like 1 isoform 2 | XP_001098299.1 |
| 3 | AT rich interactive domain 1A | O14497 |
| 4 | ATP-dependent DNA helicase II, 70 kDa subunit | XP_001105684.1 |
| 5 | basic leucine zipper and W2 domains 2 | XP_001104484.1 |
| 6 | bromodomain adjacent to zinc finger domain, 1B isoform 2 | XP_001111145.1 |
| 7 | chromatin-specific transcription elongation factor large subunit | XP_001096507.1 |
| 8 | chromobox homolog 3 | XP_001095397.1 |
| 9 | Chromobox protein homolog 5 (Heterochromatin protein 1 homolog alpha) (HP1 alpha) (Antigen p25) | XP_001091785.1 |
| 10 | DEAD/H (Asp-Glu-Ala-Asp/His) box polypeptide 3 | XP_001095294.1 |
| 11 | DEAH (Asp-Glu-Ala-His) box polypeptide 15 isoform 5 | XP_001106067.1 |
| 12 | DNA (cytosine-5-)-methyltransferase 1 | XP_001104704.1 |
| 13 | DNA-directed RNA polymerase II largest subunit (RPB1) | XP_001118060.1 |
| 14 | Eukaryotic translation elongation factor 1 | Q3YAP9_MACMU |
| 15 | eukaryotic translation elongation factor 1 delta (guanine nucleotide exchange protein) | XP_001097290.1 |
| 16 | eukaryotic translation initiation factor 3, subunit 5 epsilon, 47kDa isoform 3 | XP_001105893.1 |
| 17 | eukaryotic translation initiation factor 3, subunit 6 interacting protein, partial | XP_001116978.1 |
| 18 | eukaryotic translation initiation factor 3, subunit 9 eta, 116kDa | XP_001086876.1 |
| 19 | eukaryotic translation initiation factor 3, subunit A | XP_001102472.1 |
| 20 | fibrillarin | XP_001088664.1 |
| 21 | Guanine nucleotide-binding protein beta subunit 2-like 1 (RACK1) isoform 4 | XP_001105066.1 |
| 22 | heterogeneous nuclear ribonucleoprotein H1 isoform 8 | XP_001100049.1 |
| 23 | integrase interactor 1 | Q12824 |
| 24 | interleukin enhancer binding factor 3 isoform a | XP_001102411.1 |
| 25 | laminin, beta 1 | XP_001090393.1 |
| 26 | metastasis associated 1 family, member 3 isoform 4 | XP_001110956.1 |
| 27 | metastasis-associated protein 2 | XP_001116490.1 |
| 28 | methyl-CpG binding domain protein 3 | XP_001095979.1 |
| 29 | minichromosome maintenance protein 3 isoform 3 | XP_001106966.1 |
| 30 | minichromosome maintenance protein 6 isoform 1 | XP_001096068.1 |
| 31 | mRNA decapping enzyme | XP_001109581.1 |
| 32 | myosin, heavy polypeptide 9, non-muscle | XP_001083662.1 |
| 33 | NIMA (never in mitosis gene a)-related kinase 7 | XP_001110532.1 |
| 34 | nuclear mitotic apparatus protein 1 isoform 13 | XP_001114209.1 |
| 35 | nucleolar protein 5A | XP_001110561.1 |
| 36 | nucleolar protein NOP5/NOP58 | XP_001110561.1 |
| 37 | nucleolin | XP_001116949.1 |
| 38 | nucleophosmin 1 isoform 1 isoform 1 | XP_001099639.1 |
| 39 | p400 SWI2/SNF2-related protein | AY044869.1 |
| 40 | poly(A) binding protein, cytoplasmic 1 isoform 2 | XP_001098239.1 |
| 41 | Protein BRG-1 | [P51532](http://www.uniprot.org/uniprot/P51532) |
| 42 | retinoblastoma binding protein 4 isoform 6 | XP_001104415.1 |
| 43 | retinoblastoma binding protein 7 isoform 4 | XP_001103800.1 |
| 44 | ribonuclease P | XP_001104161.1 |
| 45 | RNA polymerase I subunit | XP_001096103.1 |
| 46 | RuvB-like 2-like protein | tr|A6MK39|A6MK39_CALJA |
| 47 | sarcoma antigen NY-SAR-41, partial | XP_001102806.1 |
| 48 | Septin-9 (MLL septin-like fusion protein), partial | XP_001116653.1 |
| 49 | SMC1 structural maintenance of chromosomes 1-like 1 | XP_001091228.1 |
| 50 | splicing factor, arginine/serine-rich 1 (ASF/SF2) | XP_001103473.1 |
| 51 | SWI/SNF complex 170 kDa subunit | Q8TAQ2 |
| 52 | SWI/SNF-related matrix-associated actin-dependent regulator of chromatin a5 isoform 2 | XP_001093597.2 |
| 53 | TIP120 protein | XP_001106780.1 |
| 54 | transcription factor ELYS | XP_001088135.1 |
