# Supplementary material for: Distinct host cell proteins incorporated by SIV replicating in CD4+ T Cells from natural disease resistant versus non-natural disease susceptible hosts
Source: Retrovirology. 2010 Dec 16;7:107. doi: 10.1186/1742-4690-7-107 (PMC3012658; doi:10.1186/1742-4690-7-107)
Supplement: Additional file 6 — A list of proteins found in common between our database and those from Yeung et al. A list of host proteins that were identified in virus preparations from rhesus macaques and sooty mangabeys and also by the studies of Yeung et al. [29]. [file 1742-4690-7-107-S6.DOC]

**Additional file 6: Proteins in common with Yeung, M. L. et al J. Biol. Chem. 284**:19463. 2009

|  | **Protein Name** | **Reference** |
| --- | --- | --- |
| 1 | actin-like 6A | NP_001098029.1 |
| 2 | adipose differentiation-related protein isoform 2 | XP_001110125.1 |
| 3 | bromodomain and WD repeat domain containing 1 isoform 1 | XP_001108655.1 |
| 4 | BTB/POZ KELCH domain protein | XP_001086777.1 |
| 5 | calcium activated chloride channel 4 | XP_001109489.1 |
| 6 | CD3e molecule, epsilon (CD3-TCR complex) | XP_001097204.1 |
| 7 | dynein, cytoplasmic, heavy polypeptide 1 | XP_001112455.1 |
| 8 | erythrocyte membrane protein band 4.1 like 5 | XP_001088764.1 |
| 9 | eukaryotic translation elongation factor 1 delta (guanine nucleotide exchange protein) | XP_001097290.1 |
| 10 | GDP-mannose 4,6-dehydratase | XP_001089757.1 |
| 11 | glyoxalase I | XP_001117098.1 |
| 12 | GTP-binding protein PTD004 isoform 6 | XP_001088356.1 |
| 13 | heat shock 70 protein 1B | XP_001115060.1 |
| 14 | leucine zipper protein 1 | XP_001111566.1 |
| 15 | methionine-tRNA synthetase isoform 4 | XP_001116063.1 |
| 16 | moesin | XP_001100546.1 |
| 17 | myosin, light polypeptide 6, alkali, smooth muscle and non-muscle isoform 7 | XP_001114459.1 |
| 18 | neuropilin 1 isoform 12 | XP_001087258.1 |
| 19 | neuropilin 2 isoform 6 precursor | XP_001104807.1 |
| 20 | Olfactory receptor | tr|Q6SN75|Q6SN75_CERAG |
| 21 | poly(A) binding protein, cytoplasmic 1 isoform 2 | XP_001098239.1 |
| 22 | proteasome (prosome, macropain) subunit, alpha type, 3 isoform 3 | XP_001091430.1 |
| 23 | protein kinase, cAMP-dependent, catalytic, beta isoform 10 | XP_001106097.1 |
| 24 | protein tyrosine phosphatase, non-receptor type 1 isoform 3 | XP_001096290.1 |
| 25 | RAB11a, member RAS oncogene family | XP_001103732.1 |
| 26 | Rho GTPase activating protein 1 | XP_001101907.1 |
| 27 | ribonuclease P | XP_001104161.1 |
| 28 | ribosomal protein L10 | XP_001086567.1 |
| 29 | ribosomal protein L3 isoform a | XP_001095608.1 |
| 30 | ribosomal protein S27 | XP_001084710.1 |
| 31 | Semenogelin I isoform a preproprotein | tr|A4K2R7|A4K2R7_GORGO |
| 32 | semenogelin II | XP_001109304.1 |
| 33 | serine/threonine protein kinase MASK | XP_001096161.1 |
| 34 | SH3 multiple domains 1 | XP_001113943.1 |
| 35 | small inducible cytokine subfamily E, member 1 | XP_001083775.1 |
| 36 | tubulin, alpha 1 isoform 3 | XP_001108924.1 |
| 37 | tubulin, alpha, ubiquitous isoform 19 | XP_001108104.1 |
| 38 | Tyrosine protein phosphatase non-receptor type 6-like protein | tr|A6MKG8|A6MKG8_CALJA |
| 39 | vacuolar H+ ATPase C2 | XP_001095057.1 |
| 40 | X-prolyl aminopeptidase (aminopeptidase P) 1, soluble | XP_001085192.1 |
