# Supplementary figures and images for: Distinct host cell proteins incorporated by SIV replicating in CD4+ T Cells from natural disease resistant versus non-natural disease susceptible hosts
Source: Retrovirology. 2010 Dec 16;7:107. doi: 10.1186/1742-4690-7-107 (PMC3012658; doi:10.1186/1742-4690-7-107)

## Slide 1
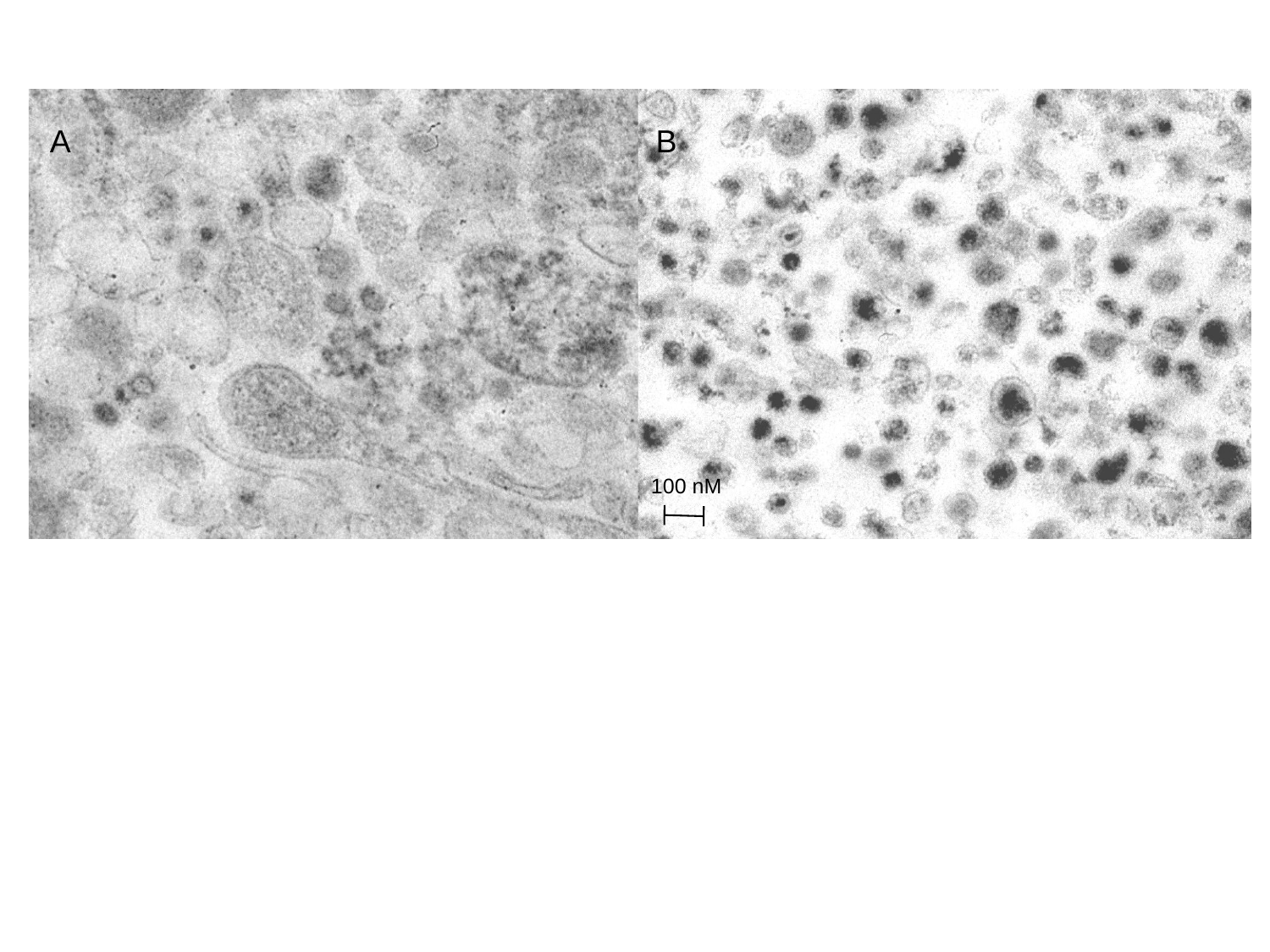

A
 B
100 nM

Supplement: Additional file 7 — Representative Electron micrographs of virus preparations prior to and post purification. Aliquots of virus preparations prior to enrichment and following purification/enrichment were pelleted and the pellets fixed in glutaraldehyde and prepared for thin section electron micrography. Particles were visualized at 90,000 × and a representative micrograph prior to (A) and post enrichment (B) is displayed. [file 1742-4690-7-107-S7.PPT]
